# Lipoprotein(a) and calcific aortic valve disease initiation and progression: a systematic review and meta-analysis

**DOI:** 10.1093/cvr/cvad062

**Published:** 2023-04-20

**Authors:** Panteleimon Pantelidis, Evangelos Oikonomou, Stamatios Lampsas, Georgios E Zakynthinos, Antonios Lysandrou, Konstantinos Kalogeras, Efstratios Katsianos, Panagiotis Theofilis, Gerasimos Siasos, Michael Andrew Vavuranakis, Alexios S Antonopoulos, Dimitris Tousoulis, Manolis Vavouranakis

**Affiliations:** 3rd Department of Cardiology, National and Kapodistrian University of Athens, Medical School, Sotiria Chest Disease Hospital, 152 Mesogeion St, Athens 11527, Greece; 3rd Department of Cardiology, National and Kapodistrian University of Athens, Medical School, Sotiria Chest Disease Hospital, 152 Mesogeion St, Athens 11527, Greece; 3rd Department of Cardiology, National and Kapodistrian University of Athens, Medical School, Sotiria Chest Disease Hospital, 152 Mesogeion St, Athens 11527, Greece; 3rd Department of Cardiology, National and Kapodistrian University of Athens, Medical School, Sotiria Chest Disease Hospital, 152 Mesogeion St, Athens 11527, Greece; 3rd Department of Cardiology, National and Kapodistrian University of Athens, Medical School, Sotiria Chest Disease Hospital, 152 Mesogeion St, Athens 11527, Greece; 3rd Department of Cardiology, National and Kapodistrian University of Athens, Medical School, Sotiria Chest Disease Hospital, 152 Mesogeion St, Athens 11527, Greece; 3rd Department of Cardiology, National and Kapodistrian University of Athens, Medical School, Sotiria Chest Disease Hospital, 152 Mesogeion St, Athens 11527, Greece; 3rd Department of Cardiology, National and Kapodistrian University of Athens, Medical School, Sotiria Chest Disease Hospital, 152 Mesogeion St, Athens 11527, Greece; 3rd Department of Cardiology, National and Kapodistrian University of Athens, Medical School, Sotiria Chest Disease Hospital, 152 Mesogeion St, Athens 11527, Greece; Cardiovascular Division, Brigham and Women's Hospital, Harvard Medical School, 75 Francis St, Boston, MA 02115, USA; 3rd Department of Cardiology, National and Kapodistrian University of Athens, Medical School, Sotiria Chest Disease Hospital, 152 Mesogeion St, Athens 11527, Greece; Clinical, Experimental Surgery & Translational Research Center, Biomedical Research Foundation of the Academy of Athens, 4 Soranou Ephessiou St, Athens 11527, Greece; 1st Department of Cardiology, National and Kapodistrian University of Athens, Medical School, Ippokrateio Hospital, 114 Vasilissis Sofias St, Athina 11527, Greece; 3rd Department of Cardiology, National and Kapodistrian University of Athens, Medical School, Sotiria Chest Disease Hospital, 152 Mesogeion St, Athens 11527, Greece

**Keywords:** Lipoprotein(a), Lp(a), Calcific aortic valve disease, Aortic valve stenosis, Meta-analysis

## Abstract

Although evidence indicates the association of lipoprotein(a) [Lp(a)] with atherosclerosis, the link with calcific aortic valve disease (CAVD) is unclear. This systematic review and meta-analysis explores the connection between Lp(a) and aortic valve calcification and stenosis (AVS). We included all relevant studies, indexed in eight databases, up to February 2023. A total of 44 studies (163 139 subjects) were included, with 16 of them being further meta-analysed. Despite considerable heterogeneity, most studies support the relationship between Lp(a) and CAVD, especially in younger populations, with evidence of early aortic valve micro-calcification in elevated-Lp(a) populations. The quantitative synthesis showed higher Lp(a) levels, by 22.63 nmol/L (95% CI: 9.98–35.27), for patients with AVS, while meta-regressing the data revealed smaller Lp(a) differences for older populations with a higher proportion of females. The meta-analysis of eight studies providing genetic data, revealed that the minor alleles of both rs10455872 and rs3798220 *LPA* gene loci were associated with higher risk for AVS (pooled odds ratio 1.42; 95% CI: 1.34–1.50 and 1.27; 95% CI: 1.09–1.48, respectively). Importantly, high-Lp(a) individuals displayed not only faster AVS progression, by a mean difference of 0.09 m/s/year (95% CI: 0.09–0.09), but also a higher risk of serious adverse outcomes, including death (pooled hazard ratio 1.39; 95% CI: 1.01–1.90). These summary findings highlight the effect of Lp(a) on CAVD initiation, progression and outcomes, and support the early onset of Lp(a)-related subclinical lesions before clinical evidence.

## Graphical Abstract

**Graphical Abstract:**
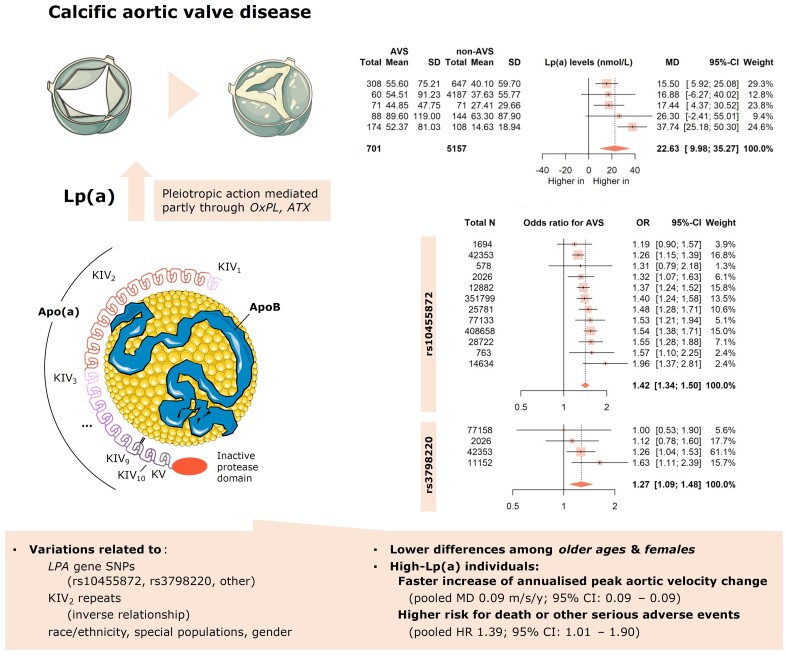

## 1. Introduction

Calcific aortic valve stenosis (AVS) is present in approximately 0.4% of the general population and 2% of individuals over 65 years, constituting one of the most common age-related valvulopathies.^1,2^ Beyond traditional risk factors, lipoprotein(a) [Lp(a)] has also emerged as a new risk factor for calcific aortic valve disease (CAVD), mediating aortic valve calcification (AVC) and AVS.^3,4^ The distinctive ‘footprint’ of the lipo-proteinic Lp(a) molecule, apolipoprotein(a) [apo(a)], involves two kringles (10 subtypes of KIV, with subtype KIV_2_ appearing with a variable number of copies, and a single copy of KV) and an inactive protease domain, which grants its multiple atherogenic and proinflammatory actions.^5^ The levels of Lp(a) are mainly genetically determined, mostly by the *LPA* gene, which dictates the size of apo(a) and concentration of Lp(a).^6^ Although evidence exists to link CAVD with Lp(a) levels, the level of awareness among physicians is still low, with Lp(a) being measured at rates lower than 5% for populations at risk,^7^ even in the light of recent guidelines that proclaim its value.^8^ Moreover, the detailed picture of this connection, particularly with regard to demographic, genetic and other interfering factors, is still obscure, a fact reflected by the absence of concrete summary risk estimates regarding the Lp(a)-related outcomes.

This systematic review and meta-analysis summarises the existing evidence regarding the role of elevated Lp(a) in AVC and AVS onset and progression, and aims to highlight the heterogeneity of related data, providing an up-to-date, comprehensive view of this relationship.

## 2. Methods

### 2.1 Search strategy

This systematic review was conducted following the Preferred Reporting Items for Systematic Review and Meta-Analysis Protocols statement. The protocol has been prospectively registered in PROSPERO (ID: CRD42022311283). We systematically searched PubMed, Embase, Scopus, Web of Science, ScienceDirect, Cochrane Library, OpenGrey, and LILACS, for articles examining the effect of lipoprotein(a) and relevant genetic factors on AVC and AVS, published from inception until February 2023. The full list of queries per database can be found in Supplementary material online, *Appendix S1*. Two researchers independently assessed the articles for eligibility, based on predefined selection criteria. Any discrepancies were resolved through repeated reviewing and consensus among the authors.

### 2.2 Selection criteria and data extraction

All studies meeting the following criteria were included in the qualitative synthesis: All types of observational studies (cohort, registry-based cohort, case-control and cross-sectional) in English language, reporting original data published in peer-reviewed journals, aligning with the following PECO framework: (i) Participants—General population or specific population groups; (ii) Exposure—High soluble plasma levels of Lp(a); (iii) Comparator—Normal/low Lp(a) levels; and (iv) Outcomes—AVC or AVS. We also retrieved data from studies exploring the association between relevant genetic risk factors [*LPA* single-nucleotide polymorphisms (SNPs) and KIV_2_ repeats] and AVC or AVS. First author’s name, study type and setup, sample size, demographics and other characteristics, Lp(a) levels and method/unit of measurement, relevant *LPA* SNPs and KIV_2_ repeats with their distribution among groups, the ascertainment method of reported outcomes, risk estimates [risk (RR), odds (OR), or hazard (HR) ratio] for AVC/AVS and related outcomes or Lp(a) level differences between compared groups (depending on study design) and, finally, information regarding the risk of bias, were extracted (see Supplementary material online, *Appendix S2*). The Newcastle–Ottawa Scale (NOS) tool was used to evaluate the risk of bias (see Supplementary material online, *Appendix S3*).

### 2.3 Statistical analysis

Meta-analysis was performed to pool the standardised mean difference in Lp(a) exposure levels (measured in nmol/L or mg/dL, on a continuous scale), between AVS and non-AVS patient groups. A sensitivity analysis was performed by excluding studies reporting Lp(a) in mg/dL. We also pooled the Lp(a) level differences between patients with severe/requiring intervention AVS and those with milder disease. The pooled mean difference in annualised peak aortic velocity change (measured in m/s/year), between high- and low-Lp(a) individuals, was calculated, along with the pooled risk of serious adverse outcomes, including death, aortic valve replacement and AVS-related hospitalisation. We also calculated the pooled OR for certain *LPA* SNPs (rs10455872 and rs3798220) between AVS and non-AVS subjects. Finally, we performed meta-regression to investigate the effect of cofactors on pooled effects, using maximum-likelihood as τ^2^ estimator. The significance threshold was set to 0.05. All statistical analyses were performed in R (version 4.2.0). A detailed description of the methodology can be found in Supplementary material online, *Appendix S2*.

## 3. Results

### 3.1 Search results and study characteristics

**Figure 1:**
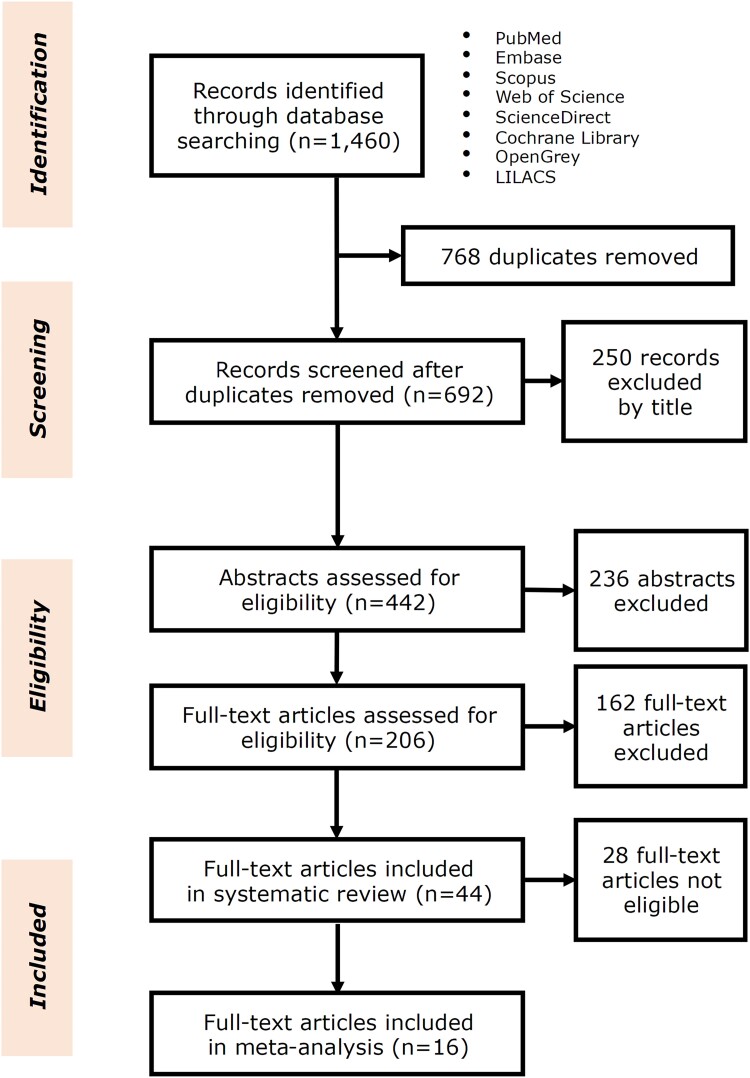
PRISMA flowchart for study selection.

From the initial 1460 titles, 44 of them were finally included in the systematic review,^7,9–51^ with 16 being eligible for meta-analysis (*Figure 1*).^7,12,15,18–21,24,35–37,41,45,49–51^ A total sample size of *n* = 163 139 subjects, with data on CAVD and Lp(a) levels, was considered (accounting for duplicate cohorts leveraged in more than one study), with a mean/median age from 45 to 80.5 years and a sample-weighted average female-to-male ratio of 1.13/1 (range: 0.16–2.03/1). Most studies (43%) were case-control, while 25% adopted a cohort and 32% a cross-sectional design. In total, 31 (70.5%) studies contained data related to stenosis and 19 (43.2%) to calcification of the aortic valve. While most projects were based in the USA the majority of included subjects originated from Denmark (approximately 47.6% of the total sample size), mostly belonging to the Copenhagen General Population Study. Samples from this cohort, along with subjects from the European Prospective Investigation into Cancer-Norfolk study, the Cardiovascular Health Study, the Aortic Stenosis Progression Observation: Measuring Effects of Rosuvastatin trial, the Multi-Ethnic Study of Atherosclerosis (MESA), the Copenhagen City Heart Study and the Atherosclerosis Risk in Communities (ARIC) study, were included in more than one studies. Only 10 (22.7%) studies used Lp(a) measurement kits with molar quantification (reporting in nmol/L), while the remaining 34 measured Lp(a) solely in mg/dL or quantified it in different ways (e.g. the cholesterol content mass). An additional total of 17 studies provided data on Lp(a)-related genetic risk and CAVD,^9,20,23,29,31,32,41,44,52–60^ with eight of them being further meta-analysed.^9,41,52–57^ A detailed description of all study characteristics and findings can be found in *Table 1*, with an extended version in Supplementary material online, *Appendix S4*.

**Table 1:** Characteristics and findings of the included studies

Study	Sample size	Age (yrs)	Sex (female)	Compared groups	Key Findings
Arsenault *et al.*^9^	17 553	59.1 (n/a)	56%	AVS vs. non-AVS	• Higher Lp(a) levels in AVS pts [16.2 (6.3–44.5) vs. 11.6 (6.2–27.6) mg/dL; *P* = 0.025]. • 2-fold risk for AVS in pts with Lp(a) ≥ 50 mg/dL (adjusted for sex, age, smoking and LDLc).
Boakye *et al.*^10^	2283	80.5 ± 4.3	61.4%	AVC vs. non-AVC	• AVC present in 44.8% of participants, with higher rates among older, White males (58.2%). • Lp(a) independently associated with AVC [adjusted prevalence ratio: 1.09; 95% CI: 1.04–1.15; *P* = 0.001].
Bortnick *et al.*^11^	3426	72 ± 5	63%	AVC vs. non-AVC	• Higher risk of AVC for elevated Lp(a) (aRR: 1.05 per 1-SD increase; 95% CI: 1.02–1.08).
Bourgeois *et al.*^12^	232	64.8 ± 9	42.7%	CAVS vs. non-CAVS	• Borderline difference in Lp(a) between CAVS and non-CAVS (89.6 ± 119.0 vs. 63.3 ± 87.9 nmol/L; *P* = 0.055). • Lp(a)-bound ATX higher in CAVS pts (aOR: 2.80; 95% CI: 1.39–5.66; *P* = 0.003).
Bozbas *et al.*^13^	285	70.2 ± 7.3	67.3%	AVC vs. non-AVC	• Higher Lp(a) in AVC pts [27.4 (range: 13.0–47.5) vs. 19.9 (range: 10.7–36.1) mg/dL; *P* = 0.033).
Cao *et al.*^14^	4593	Range for age medians: 61–62	Range: 51.4–61.2%	Multiethnic sample grouped by Lp(a) mass, molarity and cholesterol content	• CAVD positively associated with Lp(a) mass, molarity and cholesterol content, either with their upper 25th or 15th percentile as cut-off (*P* < 0.001, for all associations). • Blacks had higher Lp(a) levels, while Whites and Hispanics showed higher AVC prevalence.
Capoulade *et al.*^15^	220	58 ± 13	40%	Mild/moderate AVS—Association of Lp(a) and OxPL with AVS progression	• AVS progression faster for top Lp(a) tertiles (*V*_peak_ change: + 0.26 ± 0.26 vs. + 0.17 ± 0.21 m/s/year; *P* = 0.005) and OxPL-apoB tertiles (+0.26 ± 0.26 m/s/year vs. + 0.17 ± 0.21 m/s/year; *P* = 0.01), during 3.5 ± 1.2 yrs of follow-up. • Pts in the top tertiles of Lp(a) or OxPL-apoB were at increased risk of AV replacement and cardiac death.
Capoulade *et al.*^16^	220	58 ± 13	40%	Mild/moderate AVS pts—Association of Lp(a) with the outcomes	• CAVS progression associated with Lp(a) levels (OR: 1.10 per 10-mg/dL increase; 95% CI: 1.03–1.19; *P* = 0.006), and OxPL (∼3.5 yrs of follow-up). • Stronger association for younger ages (OR: 1.19 per 10-mg/dL increase; 95% CI: 1.07–1.33; *P* = 0.002).
Capoulade *et al.*^17^	218	58 ± 13	40%	Mild/moderate AVS pts, grouped by Lp(a) levels	• Elevated Lp(a) associated with faster progression rate of AVS (*P* < 0.001 for *V*_peak_ change, and *P* = 0.03 for risk of AV replacement/cardiac death), over a median of 3.5 yrs.
Chen *et al.*^18^	3067	61.1 ± 7.5	53.4%	CAVS vs. non-CAVS	• Lp(a) was higher in CAVS pts [9.1 (3.7–24.8) vs. 6.1 (3.2–14.3) mg/dL; *P* < 0.001], with a multivariably-adjusted aOR: 1.002 (95% CI: 1.001–1.002; *P* < 0.001).
Despres *et al.*^19^	496	68.2 (n/a)	38.36%	CAVS vs. non-CAVS	• Higher Lp(a) in CAVS pts [28.7 (8.2–116.6) vs. 10.9 (3.6–28.8) nmol/L; *P* < 0.0001). Similarly, CAVS pts had higher OxPL (*P* < 0.001). • Higher TBR_mean_ (^18^FNaF PET uptake) for pts with no CAVS, but elevated Lp(a).
Dong *et al.*^20^	219	63.72 (n/a)	53.5%	CAVD vs. CAD vs. non-CAVD/CAD	• Higher Lp(a) levels in CAVD [37.2 (16.5–79.6) nmol/L] and CAD [46.7 (21.5–104.6) nmol/L] pts, as compared to controls [23.6 (9.4–48.6) nmol/L; *P* < 0.001]. • Rs6415084, rs3798221 and rs7770628 affected Lp(a) levels.
Glader *et al.*^21^	202	71.3 ± 8.5	40.6%	Significant AVS vs. non-AVS matched controls	• High Lp(a) (≥48 mg/dL) pts in greater risk for AVS requiring intervention (aOR: 3.4; 95% CI: 1.1–11.2).
Gotoh *et al.*^22^	784	62 ± 11	55.7%	High (≥30 mg/dL) vs. low (<30 mg/dL) Lp(a)	• Higher rates of AVC for Lp(a) ≥ 30 mg/dL (36.1% vs. 12.7%; *P* < 0.001). • Lp(a) levels higher in women (*P* < 0.01) and not related with age. Difference in Lp(a), between AVC and non-AVC groups, higher in female subpopulation.
Gudbjartsson *et al.*^23^	12 137 with Lp(a) levels	n/a	n/a	Association of Lp(a) levels and KIV-2 repeats with AVS	• AVS associated with higher Lp(a), with aOR: 1.17 per 50 nmol/L increase (95% CI: 1.12–1.22; *P* < 0.0001). • Fewer KIV-2 repeats related to higher Lp(a) molarity and larger apo(a) isoform size. Carriers of G4925A (KIV-2 mutation) had small apo(a) and lower Lp(a). • KIV-2 repeats associated with CVD, only before adjusting for Lp(a) molarity.
Hojo *et al.*^24^	861	73 (66–78)	20.9%	Pts with PAD, grouped by AVS presence and other valvulopathies	• Higher Lp(a) in AVS pts [34.0 (16.7–50.0) vs. 20.0 (11.0–35.0) mg/dL; *P* = 0.002]. • Lp(a) associated with LDLc and high-sensitivity CRP levels (*P* < 0.05).
Hovland *et al.*^25^	78	49 ± 15 for FH pts; 44 ± 12 for controls	55% for FH pts; 57% for controls	Heterozygous FH pts, grouped by Lp(a) levels and controls	• Lp(a) levels similar for FH and controls, measured either in mass or molarity. • AVA and *V*_peak_ did not differ between high (>75 nmol/L) and low Lp(a) FH pts (*P* = 0.3 and *P* = 0.65, respectively). • Higher *V*_peak_ and smaller AVA for FH pts [1.2 (1.1–1.5) vs. 1.0 (1.0–1.1) m/s; *P* = 0.02, and 2.5 ± 0.6 vs. 2.8 ± 0.6 cm^2^; *P* = 0.04, respectively].
Kaiser *et al.*^26^	58	66.4 ± 5.6	15.4%	Mild/moderate AVS pts, divided by Lp(a) levels (cut-off: 50 mg/dL)	• No difference between Lp(a) groups, in AV calcium score [1388 (450–2424) vs. 1173 (927–1628) AU, respectively; *P* = 0.839] or ^18^FNaF PET uptake (TBR_mean_: 3.02 ± 1.26 vs. 3.05 ± 0.96, respectively; *P* = 0.902).
Kaiser *et al.*^27^	3271	63.3 ± 13.3	53%	Association of AVC with Lp(a)	• Dose-dependent relationship between Lp(a) and AVC, with aOR: 1.89 (95% CI: 1.48–2.42; *P* < 0.001) for 80th-94th and aOR: 2.84 (95% CI: 1.96–4.10; *P* < 0.001) for >95th percentiles.
Kaiser *et al.*^28^	922	66 ± 4.2	52.3%	Three groups according to AVC status at baseline and follow-up	• Lp(a) levels associated with baseline and new-onset AVC [aOR: 1.43; 95% CI: 1.15–1.79 and aOR: 1.30; 95% CI: 1.02–1.65, respectively, for each 50 mg/dL increase in Lp(a), but not with AVC progression.
Kaltoft *et al.*^29^	12 006 with CT/85 884 in total	59.2 (51.1–67) for the sub-cohort with CT	57%	Association of Lp(a) and *LPA* genotypes with AVC and AVS	• Elevated Lp(a) increased AVC and AVS risk (aOR: 1.62; 95% CI: 1.48–1.77, and aHR: 1.54; 95% CI: 1.38–1.71, respectively, for 10-fold increase in Lp(a)). • KIV-2 repeats and rs10455872 associated with increased AVC (aOR: 2.23; 95% CI: 1.81–2.76, and aOR: 1.86; 95% CI: 1.64–2.13, respectively.
Kaltoft *et al.*^30^	69 988	60 (range: 20–100)	54%	Grouped by Lp(a) levels: Low, ≤ 9 vs. moderate, 10–68 vs. high, ≥ 69 mg/dL	• Moderate and high Lp(a) associated with increased CAVD risk, over ∼7.4 yrs (aHR: 1.28; 95% CI: 1.13–1.44, and 1.86; 95% CI: 1.57–2.21, respectively). • Men and pts with increased BMI had a higher 10-year CAVD risk.
Kamstrup *et al.*^31^	77 680	58 (47–67)	56%	Risk of AVS for different Lp(a) percentiles and *LPA* genotypes	• Dose-dependent relationship between Lp(a) and AVS risk, with aHR: 1.2 (95% CI: 0.8–1.7) for 22nd-66th, 1.6 (95% CI: 1.1–2.4) for 67th-89th, 2.0 (95% CI: 1.2–3.4) for 90th- 95th and 2.9 (95% CI: 1.8–4.9) for >95th Lp(a) percentiles. • Higher Lp(a) levels and AVS risk associated with rs10455872 and rs3798220 minor alleles, and low KIV-2 repeats (*P* < 0.05).
Kamstrup *et al.*^32^	2138	74 (67–79)	37%	CAVD vs. non-CAVD	• Lp(a) higher in CAVD group [12 (4–48) vs. 8 (4–24) mg/dL; *P* < 0.001]. • For every 10 mg/dL-increase in Lp(a) aOR: 1.10 (95% CI: 1.06–1.13) for CAVD. • OxPL-apoB and OxPL-apo(a) correlated with Lp(a) and associated with CAVD.
Langsted *et al.*^33^	52 652 with Lp(a) levels	58 (48–68)	54.3%	Association of Lp(a) and *LPA* genotypes with AVS	• Lp(a) associated with higher risk for AVS (aHR: 1.23 for every 1-SD increase; 95% CI: 1.06–1.41). • *LPA* SNPs (rs10455872, rs3798220) and KIV-2 repeats also associated with higher AVS risk.
Littmann *et al.*^34^	1860	48 ± 16	44%	Pts with type 1 diabetes mellitus grouped by Lp(a) levels	• Elevated Lp(a) associated with higher risk for CAVD (aRR = 2.03; *P* < 0.05, for Lp(a) > 120 nmol/L).
Liu *et al.*^35^	652 at baseline/359 with follow-up	62 ± 17	41.9%	Mild/moderate CAVS pts, grouped by Lp(a) levels (cut-off: 38.15 mg/dL)	• Higher baseline *V*_peak_ (3.70 ± 1.12 vs. 3.43 ± 1.14 m/s; *P* = 0.012) and higher rates of severe AVS (aOR: 1.78; 95% CI: 1.18–2.66; *P* = 0.006) for pts with elevated Lp(a). • After 3.16 ± 2.74 yrs of follow-up (excluding severe AVS cases), only age was a significant predictor of AV-related surgery or death.
Ljungberg *et al.*^36^	955 with Lp(a) levels	56.7	48%	AVS pts requiring surgery vs. non-AVS controls*	• Higher Lp(a) in AVS pts [55.6 (47.2–64.0) vs. 40.1 (35.6–44.7) nmol/L; *P* = 0.005] • Severe AVS [52.9 (44.4–61.4) nmol/L] corresponded to lower Lp(a) than mild/moderate [77.8 (47.7–107.0)].
Mahabadi *et al.*^37^	968	80 ± 5	48%	Severe AVS vs. non-AVS	• Median Lp(a) did not differ between severe-AVS and non-AVS pts [17 (8–56) vs. 18.5 (8.5–57) mg/dL; *P* = 0.56). Outcome retained in risk factor-adjusted model (aOR: 0.98; 95% CI: 0.90–1.06; *P* = 0.57).
Makshood *et al.*^38^	5366	Range for age mean: 59.3–62.4	Range: 51.4–57.0%	Grouped by race/ethnicity	• Higher Lp(a) levels for South Asians (17.0 mg/dL) than all other groups (12.9–13.1 mg/dL), except Blacks (35.1 mg/dL). • Lower AVC rates in Chinese (6.6%), with higher in Whites (14.6%) and Hispanics (13.2%). • Lp(a) positively associated with AVC only in Blacks and Whites.
Nsaibia *et al.*^39^	300	71 ± 9	35%	CAVS & CAD vs. only CAD	• Higher levels of Lp(a) when CAVS was present (32.5 ± 36.2 vs. 23.7 ± 29.5 mg/dL; *P* = 0.003). • Lp(a) levels ≥50 mg/dL increased the risk of CAVS, only through ATX mass and activity.
Obisesan *et al.*^40^	2083	59.2 ± 4.3	62.2%	Low (≤50) vs. high (>50 mg/dL) Lp(a) levels	• Lp(a) levels >50 mg/dL independently increased the risk for AVC [aOR: 1.79; 95% CI: 1.32–2.43]. • Race or sex did not significantly affect this relationship.
Ozkan *et al.*^41^	152	72.23 (n/a)	50.7%	CAVD vs. non-CAVD	• Higher Lp(a) in CAVD pts [68.67 (67.17–70.16) vs. 27.05 (25.86–28.23) mg/dL; *P* < 0.001]. • Milder AVS corresponded to higher Lp(a) (non-significant outcome) [mild: 70.28 (68.34–72.23) vs. moderate: 69.10 (67.30–70.90) vs. severe: 67.44 (64.26–70.62); *P* = 0.388]. • Rs1055872 and rs3798220 associated with CAVD.
Simony *et al.*^42^	70 042	60 (50–69)	53.6%	Risk of AVS/other CVD for high Lp(a) levels	• In women, Lp(a) levels increased by 27% after menopause and decreased by 12% with hormone replacement therapy. • Lp(a) > 40 mg/dL independently associated with AVS, MI, CAD for both sexes.
Stewart *et al.*^43^	5114	72.7 (n/a)	57.9%	CAVD (sclerosis or stenosis) vs. non-CAVD	• Higher Lp(a) levels for CAVD pts (62.3 ± 71.4 vs. 50.7 ± 48.5 mg/dL; *P* < 0.001). • Lp(a) strongly associated with CAVD (aOR: 1.23; 95% CI: 1.14–1.32; *P* < 0.001). • Age and male sex double the risk for CAVD.
Sticchi *et al.*^44^	69	45 (30–53)	20.3%	Bicuspid aortic valve pts, grouped by AVC and AVS status	• Higher Lp(a) levels associated AVC (*P* = 0.008) and AVS status (*P* = 0.043).
Vassiliou *et al.*^45^	165	75.3 (n/a)	29.7%	AVS (severe or mild/moderate) vs. non-AVS	• Higher Lp(a) in AVS pts [30.9 (7.5–68.8) vs. 10.0 (4.1–26.6) mg/dL; *P* < 0.001]. • Severe AVS related to lower median Lp(a) levels than mild/moderate (non-significant outcome) [24.2 (7.2–70.0) vs. 38.4 (9.1–65.6) mg/dL; *P* = 0.64].
Vongpromek *et al.*^46^	129	51 ± 8	37.2%	Pts with heterozygous FH—Association of AVC with Lp(a)	• AVC present in 38.8% of pts (Ca-Score > 0). • Elevated Lp(a) increased AVC risk (aOR: 1.11 per 10-mg/dL increase; 95% CI 1.01–1.20; *P* = 0.03).
Wang *et al.*^47^	152	70	43.4%	Symptomatic pts grouped by AVC status	• Lp(a) associated with calcification grade (1.21 ± 0.30 in heavy, 1.41 ± 0.32 in moderate, 1.61 ± 0.34 in mild and 1.63 ± 0.38 in absent AVC; *P* < 0.01, log-transformed data in nmol/L). • Lp(a) associated with AVC in multifactorial model (aOR: 1.04; 95% CI: 1.01–1.06; *P* = 0.005), along with PCSK9 and age.
Wang *et al.*^48^	410	58.6 ± 10.8	14.1%	AVC vs. non-AVC	• Lp(a) was higher in AVC pts with new-onset MI [23.2 (11.1–42.5) vs. 15.4 (6.8–30.8) mg/dL; *P* < 0.001]. • Lp(a) levels were independently associated with CAVS in a non-linear fashion.
Wilkinson *et al.*^7^	4079	75 (n/a)	47%	CAVS vs. non-CAVS	• Low rates (<5%) of Lp(a) measurement. • Non-significant difference in Lp(a) levels for CAVS and non-CAVS [14 (6–48) vs. 15.5 (6.5–63) mg/dL; *P* = 0.734].
Wodaje *et al.*^49^	23 298	55.5 ± 17.1	52%	CAVS vs. non-CAVS	• Higher Lp(a) levels for CAVS pts [20.2 (7.6–63.7) vs. 17 (6.6–43.6) mg/dL; *P* = 0.009; *n* = 19 151], for both sexes. • Lp(a) levels >90th percentile increased the risk for AVS (aHR: 1.53; 95% CI: 1.08–2.15; *P* = 0.016; age, sex adjusted).
Zheng *et al.*^50^	17 745	59.2 ± 9.1	55.1%	AVS vs. non-AVS	• Higher levels of Lp(a) for AVS pts [15.3 (7.0–41.7) vs. 11.7 (6.3–27.7) mg/dL; *P* < 0.001]. • Lp(a) > 50 mg/dL independent risk factor for AVS (aHR: 1.70; 95% CI: 1.33–2.19; *P* < 0.001; age, sex, LDLc, CAD adjusted).
Zheng *et al.*^51^	145	70.3 ± 9.9	31.7%	Pts with AVS, grouped by Lp(a) levels (cut-off: 35 mg/dL)	• At baseline, top Lp(a) tertile pts (>35 mg/dL) showed increased ^18^FNaF PET uptake (TBR_mean_: 2.16 vs. 1.97; *P* = 0.043), but did not differ in *V*_peak_ (*P* = 0.150) or Ca-Score (*P* = 0.429). Same for OxPL. • High-Lp(a) pts showed increased Ca-Score progression [309 (142–483) vs. 93 (56–296) AU/year; *P* = 0.015], faster hemodynamic progression on echo (0.23 ± 0.20 vs. 0.14 ± 0.20 m/s/year; *P* = 0.019), and increased risk for aortic valve replacement and death (HR: 1.87; 95% CI: 1.13–3.08; *P* = 0.014), during follow-up.

All values in mean ± SD or median (IQR); *, Information obtained after contacting the authors; ^18^FNaF PET, 18F-Sodium Fluoride Positron Emission Tomography; 95% CI, 95% Confidence interval; aHR, Adjusted hazard ratio; aOR, Adjusted odds ratio; ARIC, Atherosclerosis Risk In Communities study; aRR, Adjusted relative risk; ATX, Autotaxin; AU, Agatston units; AV, Aortic valve; AVA, Aortic valve area; AVC, Aortic valve calcification; AVS, Aortic valve stenosis; Ca-Score, Calcium score; CAD, Coronary artery disease; CAVD, Calcific aortic valve disease; CAVS, Calcific aortic valve stenosis; CRP, C-reactive protein; CT, Computed tomography; CVD, Cardiovascular disease; Echo, Echocardiography; FH, Familial hypercholesterolemia; KIV-2, Kringle IV-2 repeat; LDLc, Low density lipoprotein cholesterol; Lp(a), Lipoprotein(a); LPA, Lipoprotein(a) protein coding gene; n, Number; n/a, Not available; OxPL-apo(a)/-apoB, Oxidized phospholipids bound to apolipoprotein(a)/apolipoprotein B; PAD; Peripheral arterial disease; PRECISE, PolyvasculaR Evaluation for Cognitive Impairment and vaScular Events study; Pts; Patients; SD, Standard deviation; SNPs, Single nucleotide polymorphisms; TBR_mean_, Mean tissue-to-background ratio; *V*_peak_; peak aortic jet velocity; yrs, Years

### 3.2 Quality assessment

The overall quality was found high for 33 studies (75%) and moderate for the remaining 11 (25%). Cohort studies displayed an average score of 7.2 out of 9, with nine (82%) being of high quality. The same figure was 7.4/9 for case-control and 7.2/10 for cross-sectional studies (*n* = 15, 79% and *n* = 9, 64% of high quality, respectively). Supplementary material online, *Appendix S3* includes a detailed report of the NOS quality assessment results.

### 3.3 Qualitative synthesis

#### 3.3.1 Lp(a) and aortic valve stenosis

Despite the considerable heterogeneity in the design and findings of included studies, an overall trend toward increased risk of calcific AVS was observed for higher-Lp(a) groups. Several studies reported significantly higher Lp(a) levels for AVS patient groups, as compared to subjects without stenosis, with differences reaching 41.62 mg/dL.^9,24,32,41,43,45,49,50^ Elevated Lp(a) was found to raise the risk for AVS, ranging from 1.70 (95% CI: 1.33–2.19; 17 745 patients),^50^ to 3.4 (95% CI: 1.1–11.2; 202 patients),^21^ with two analyses suggesting a dose-dependent relationship.^30,31^ In most studies, AVS was associated mainly with two SNPs, rs10455872 and rs3798220,^9,54,55,57,58^ also in a dose-dependent fashion since homozygotes for minor alleles (G and C, respectively) presented with a 2- to nearly 3-fold higher risk, as compared with heterozygotes.^9,57^ Furthermore, an inverse relationship was widely found between KIV_2_ repeats and AVS,^23,31^ with KIV2 number inversely affecting Lp(a) levels.^44^ Seven studies did not find Lp(a) to associate with AVS, including one concerning patients with familial hypercholesterolemia (FH) of younger age (∼49 years),^25^ another investigating an older population with a mean age of 80 years^37^ and two more studies suggesting only an indirect relationship through autotaxin (ATX).^12,39^

#### 3.3.2 Lp(a) and aortic valve calcification

The majority of studies showed a clear association between Lp(a) and AVC, with the adjusted risk of AVC ranging from 1.05 (95% CI: 1.02–1.08) per 1-SD of Lp(a) increase^11^ to 1.79 (95% CI: 1.32–2.43) for Lp(a) levels above 50 mg/dL.^40^ AVC groups displayed higher Lp(a) levels (by 5.7–7.8 mg/dL^11,13,48^), as well as an association with the minor alleles of certain *LPA* SNPs (rs10455872^29,58,59^ and rs3798220^29^) and fewer KIV2 repeats.^29,44^ Three studies also investigated microcalcification differences between high- and low-Lp(a) groups, by quantifying 18F-sodium fluoride (^18^FNaF) uptake with positron emission tomography (PET) scanning. Zheng *et al.* found that patients with elevated Lp(a) (>35 mg/dL) displayed significantly increased valve micro-calcification, despite their similar peak aortic velocity and calcium score.^51^ After a 2- to 3-year follow-up, high-Lp(a) patients of the same study were found with increased calcium score progression and higher annualised peak aortic velocity change. Another study showed that patients with no visible AVC, yet elevated Lp(a), presented with significantly increased valve microcalcification.^19^ On the contrary, Kaiser *et al.* found no difference between high- and low-Lp(a) in patients with non-severe AVS, both in terms of calcium score and ^18^FNaF uptake,^26^ while in another study, increased Lp(a) was associated with the new-onset of AVC, but not with its progression.^28^ Noteworthy is evidence indicating a dose-dependent relationship also for calcification, with higher Lp(a) values multiplying the risk of AVC,^14,27,47^ yet in a nonlinear fashion.^48^

#### 3.3.3 Race/ethnicity heterogeneity and special populations

While most studies leverage samples of single origin or race/ethnicity, evidence from three large, multi-race/ethnic cohorts with more than 7500 subjects, provides head-to-head comparisons among such subpopulations.^10,14,38,40^ Leveraging the MESA and ‘Mediators of Atherosclerosis in South Asians Living in America’ cohorts, Makshood *et al.* suggest the contribution of race/ethnicity to Lp(a) levels, with Blacks and South Asians presenting with significantly higher median Lp(a) values than Whites, Hispanics and Chinese Americans.^38^ Moreover, race/ethnicity was found to mediate the Lp(a) effect on AVC. AVC prevalence was higher in Whites (14.6%) and Hispanics (13.2%), as compared to South Asians (10.7%), Blacks (11.7%) and Chinese (6.6%), with only Whites and Blacks demonstrating a significant association between AVC and Lp(a) levels. Two additional analyses of the ARIC cohort revealed the same trend, since AVC was more prevalent in White participants, as compared to Black, although the latter displayed higher median Lp(a) levels.^10,40^

The effect of Lp(a) on CAVD was also confirmed for patients with type I diabetes mellitus (T1DM), as shown by a study with 1860 T1DM patients (median age: 48 years), which reported a significantly increased risk of AVC for elevated-Lp(a) patients (adjusted RR: 2.03; 95% CI: 1.03–4.03, when Lp(a) > 120 mg/dL).^34^ Among subjects with bicuspid aortic valve (BAV) of younger median age (48 years), Lp(a) levels were also found significantly elevated in both the AVC and stenosis subgroups.^44^ Finally, this relationship also holds for heterozygous FH patients, as shown by Vongpromek *et al.* who found elevated Lp(a) to increase the risk of AVC (adjusted OR per 10-mg/dL increase: 1.11; 95% CI 1.01–1.20; *P* = 0.03) in a sample of 129 FH subjects with a median age of 51 years.^46^ On the contrary, Hovland *et al.* showed no association of Lp(a) with AVS in a smaller sample of 64 FH patients.^25^

### 3.4 Quantitative synthesis

**Figure 2:**
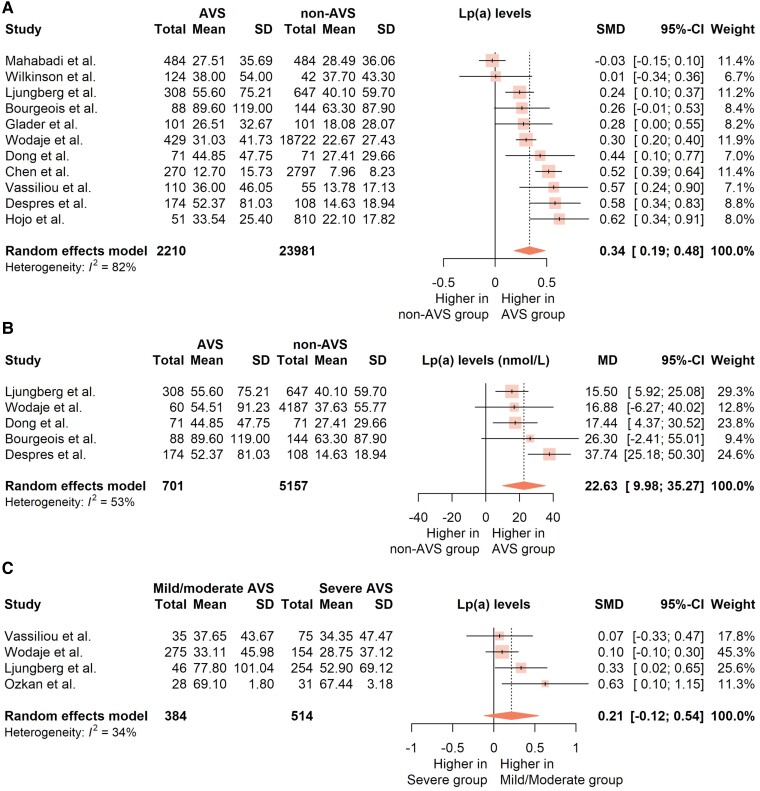
(*A*–*C*) Forest plots showing the pooled (*A*) standardised mean difference (MD) in lipoprotein(a) between patients with aortic valve stenosis and those without, (*B*) MD only for studies reporting in nmol/L (only the subcohort of individuals with measurements in nmol/L was used from the study by Wodaje *et al.*) and (*C*) standardised MD for patients with severe against those with milder stenosis. Random effects model was applied with the size of each marker corresponding to its relative study weight. AVS, Aortic valve stenosis; Lp(a), Lipoprotein(a); SD, Standard deviation; CI, Confidence interval; MD, Mean difference (SMD, Standardised MD); I^2^, Higgins’ and Thompson’s I^2^ statistic.

**Figure 3:**
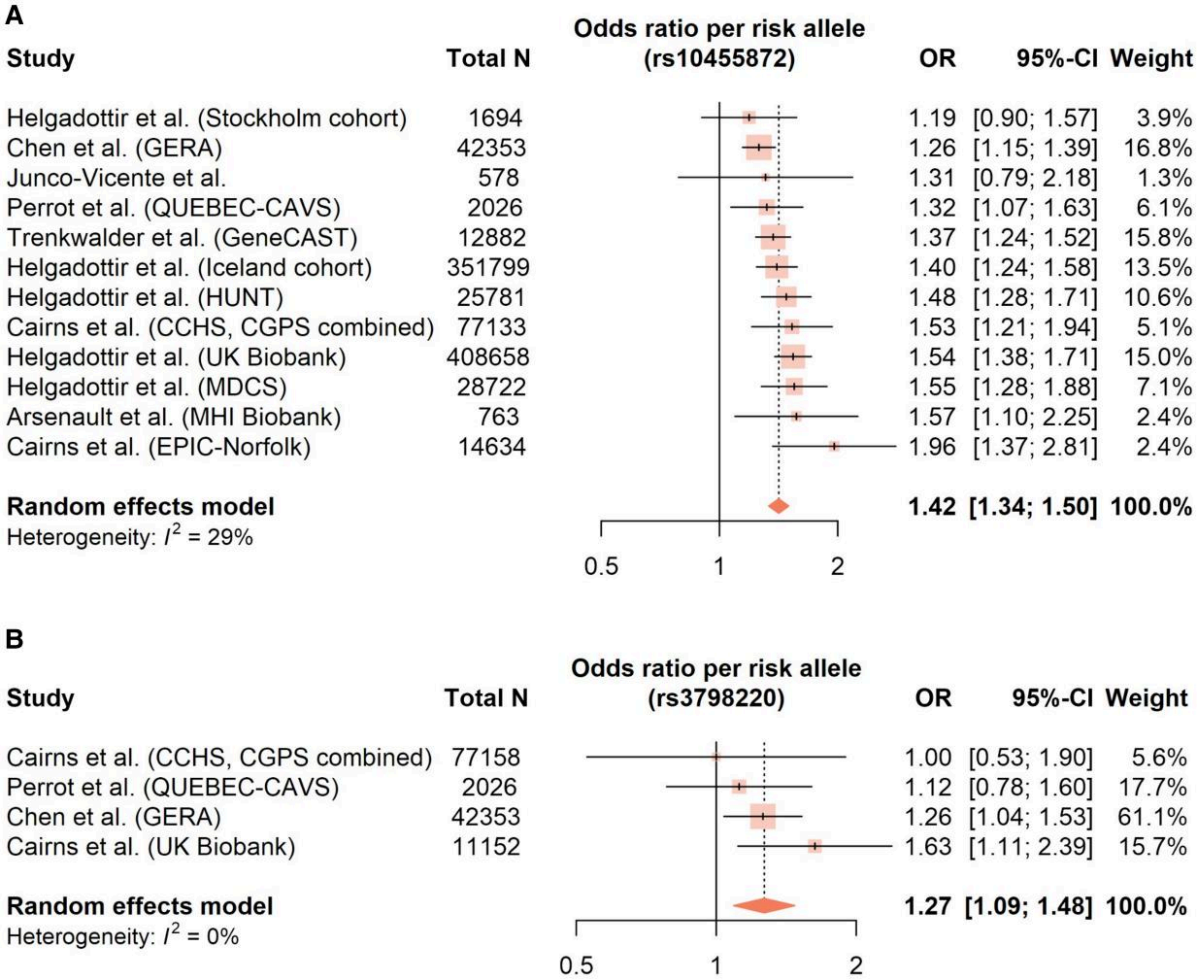
(*A* and *B*) Forest plots showing the pooled odds ratio of AVS for two *LPA* SNPs: (*A*) rs10455872 minor allele G and (*B*) rs3798220 minor allele C. Random effects model was used with the size of each marker corresponding to its relative study weight. OR, Odds ratio; CI, Confidence interval; *N*, Number of participants; I^2^, Higgins’ and Thompson’s I^2^ statistic.

After excluding outlying and influential studies, the meta-analysis of 11 studies^7,12,18–21,24,36,37,45,49^ with 26 191 subjects, showed significantly higher Lp(a) levels for AVS patients, by a standardised mean difference of 0.34 (95% CI: 0.19–0.48; *P* < 0.001; *Figure 2A*), with a low risk of publication bias. The sensitivity analysis of five studies (5858 subjects), reporting Lp(a) levels in nmol/L,^12,19,20,36,49^ confirmed the results, showing higher Lp(a) levels for AVS patients by a pooled mean difference of 22.63 nmol/L (95% CI: 9.98–35.27; *P* = 0.008; *Figure 2B*). No significant difference in Lp(a) levels was observed between severe and milder AVS cases (standardised mean difference 0.21; 95% CI: -0.12 to 0.54; *P* = 0.130; *Figure 2C*). Meta-regression identified both age and sex as significant predictors of the Lp(a) difference between AVS and non-AVS patients, with lower Lp(a) differences associated with older age (β -0.02; 95% CI: -0.035 to -0.006; *P* = 0.012) and higher percentages of female subjects (β -0.017; 95% CI: -0.03 to -0.004; *P* = 0.017). Both *LPA* SNPs were significantly associated with the risk of AVS, with pooled odds ratios 1.42 (95% CI: 1.34–1.50; *P* < 0.001; *Figure 3A*) for rs10455872 (minor allele G) and 1.27 (95% CI: 1.09–1.48; *P* = 0.002; *Figure 3B*) for rs3798220 (minor allele C). While age was inversely associated with the effect size of rs10455872 (β -0.013; 95% CI: -0.021 to -0.005; *P* = 0.008), no similar association was found for rs3798220, or sex regarding both SNPs (*P* > 0.05).

**Figure 4:**
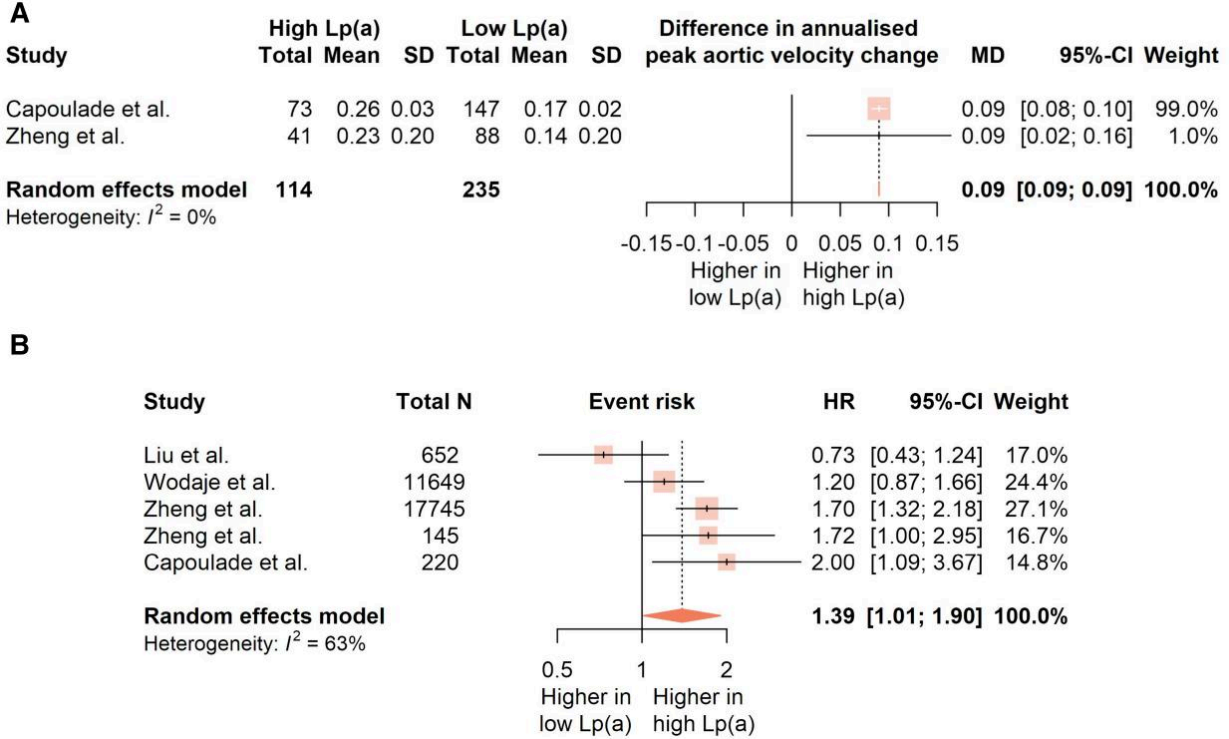
(*A* and *B*) Forest plots showing the (*A*) pooled mean difference (MD) in annualised peak aortic velocity change (measured in m/s/year), and (*B*) the pooled hazard ratio for serious adverse events (death, aortic valve replacement or stenosis-related hospitalisation), between patients with low and those with high lipoprotein(a). Random effects model was applied, with the size of each marker corresponding to its relative study weight. Lp(a), Lipoprotein(a); SD, Standard deviation; CI, Confidence interval; MD, Mean difference; HR, Hazard ratio; *N*, Number of participants; I^2^, Higgins’ and Thompson’s I^2^ statistic.

High-Lp(a) patients were found to progress faster than their low-Lp(a) counterparts to AVS, displaying a higher annualised peak aortic velocity change by 0.09 m/s/year (95% CI: 0.09–0.09; *P* < 0.001; *Figure 4A*).^15,51^ Moreover, the risk of serious adverse outcomes, including death, was higher for individuals with elevated Lp(a) (pooled hazard ratio 1.39; 95% CI: 1.01–1.90; *P* = 0.042; *Figure 4B*),^15,35,49–51^ an outcome that was retained after excluding influential studies (pooled HR: 1.56; 95% CI: 1.11–2.18; *P* = 0.025). No association was found between the adverse event risk and age (*P* = 0.84) or sex (*P* = 0.86), across studies. Supplementary material online, *Appendix S5* offers a detailed view of the meta-analysis results.

## 4. Discussion

### 4.1 Summary of findings in clinical context

Elevated Lp(a) has already been characterised as a potential causal risk factor for atherosclerotic burden and cardiovascular disease, with the most recent European Society of Cardiology (ESC)/European Atherosclerosis Society guidelines on dyslipidaemias suggesting its measurement at least once in the lifetime.^8^ However, despite the sporadic data linking Lp(a) with CAVD, there is still a lack of systematic approaches that summarise and quantify this relationship,^8^ with no definite thresholds to direct mitigation strategies, as reflected in the recent 2021 ESC/European Association of Cardio-Thoracic Surgery guidelines for the management of valvular diseases.^5,61^ Extending previous attempts to summarise existing data,^62–66^ this work focuses directly on the effect of Lp(a) circulating levels and Lp(a)-associated gene SNPs on CAVD, encompassing the most recent evidence. Moreover, it provides new insights that complement previous metanalytic approaches,^67^ by systematically standardising Lp(a) differences between CAVD patients and healthy controls and by offering additional collective evidence regarding the Lp(a)-related AVS progression acceleration and risk of serious adverse events.

Most studies confirm a link between elevated Lp(a) and increased incidence of aortic valve disease.^21,50^ There are also data supporting a dose-dependent effect.^14,27,30,31,47,48^ Genetic factors may contribute to this relationship, with the *LPA* SNPs rs10455872 (allele G) and rs3798220 (allele C) displaying the most prominent effects. Interestingly, such genetic substrates seem to increase the risk of AVS, not only by affecting the Lp(a) levels but also independently and, in fact, in a dose-dependent manner.^9^ The multifactorially determined number of KIV_2_ repeats is also associated with AVS risk, but only through determining the size of apo(a) and, consequently, the levels of Lp(a).^23,44^ Furthermore, pooling the results from available studies showed that Lp(a) not only impacts CAVD onset, but it also accelerates hemodynamic deterioration and results in significantly more deleterious outcomes, including death.^15,50,51^ This association seems to be mediated by the Lp(a)-driven AVC, since more vivid micro-calcification measured with ^18^FNaF uptake in PET scanning was observed for patients with no visible AVC but elevated Lp(a).^19,51^ Interestingly, those patients progressed faster to visible calcification and stenosis.^51^ In contrast to such evidence supporting the acceleration of CAVD progression by Lp(a), Kaiser *et al.* did not find a similar relationship in a sample of 922 individuals, yet with a considerable drop-out rate of about two-thirds.^28^ Given the impact of Lp(a) on CAVD onset and, possibly, progression, future treatment strategies might not only have a place early in life, when Lp(a) displays its original insult, but also later in order to diminish its possible effect on progression. However, more evidence is needed to determine the clinical benefit of such policies, along with the time of intervention and the extent of level reduction that is required.

As implied by our meta-regression analyses, older populations present lesser differences in Lp(a) levels between stenosis cases and controls. Although diagnostic bias, due to early atherosclerotic manifestations in high-Lp(a) subjects leading to faster diagnoses, cannot be excluded, this risk is limited since most studies, spanning across the whole range of ages, are age-matched or do not display significant age differences,^7,21,36,37^ and, furthermore, the within-study compared groups of most attempts, show similar percentages of comorbidities or predisposing risk factors.^21,36,37^ However, this paradox of wider Lp(a) differences between CAVD and healthy subjects at younger ages, might also be explained by the hypothesis that elevated Lp(a) assumes its role and serves as an initiating insult early enough, while other age-related degenerative mechanisms also come into play in older ages, diminishing the already ‘exhausted’ role of Lp(a) and finally accounting for the majority of CAVD cases.^16,27^

Similarly, Lp(a) seems to play a more limited role in populations with a higher proportion of females. Τhis variation might, in part, be attributed to other sex-related factors, such as the BAV incidence which is typically higher in males.^68^ Although some studies appear with similar or considerably low BAV rates,^7,21^ or even display an insignificant impact of BAV in sensitivity analysis,^45^ for those not reporting the BAV prevalence and distribution between compared groups, this factor could be confounding and, thus, the results should be interpreted with caution. Additionally, this variation might also be explained by the described sex-related pathophysiological differences in the development of AVS. For the same degree of hemodynamic stenosis, male subjects appear with a higher degree of aortic cusp calcification, contrary to females presenting with increased fibrosis.^69,70^ This tendency toward a more calcific, than fibrotic, pattern in men might pathophysiologically involve the action of Lp(a),^71^ resulting in its more frequent appearance in elevated-Lp(a) male subgroups. However, calcification in the cardiovascular system is not only a male feature and its attribution solely, or even predominantly, to Lp(a) is not a solid assumption, as other sex-related factors, like testosterone, are known to affect calcification in clinical and experimental animal studies.^72,73^ Such contradictory evidence calls for a deeper investigation into whether and how gender interferes with the effect of Lp(a) on CAVD.

### 4.2 Heterogeneity in study design

The considerable heterogeneity observed in the design of studies offers valuable insights regarding potential sources of variations in outcomes. At first, only 10 studies reported the molar concentration of Lp(a),^12,19,20,23,25,34,36,42,47,49^ while most of the rest quantified its mass. Since the mass of Lp(a) is heavily influenced by the highly variable apo(a) isoform size,^5,74^ mass concentrations are not linearly correlated with their molar counterparts among different individuals or populations. Therefore, direct comparisons between studies of different measurement types, or even between studies employing the same apo(a)-sensitive estimating method on heterogeneous populations, are prone to bias.^75^ Substantial variations are also encountered in the definition of outcomes, concerning both AVC, which is estimated with computed tomography scanning,^46^ echo testing,^22^ relevant International Classification of Diseases (ICD) codes on medical records^29^ or PET scan for micro-calcification,^19,26^ and AVS, assessed mostly with echo,^7,12,16,18,24,25,37,39,44^ but also with cardiac magnetic resonance imaging,^45^ medical records with relevant ICD codes, and clinical events associated with AVS.^9,30,32,49,50^ Finally, variations exist in Lp(a) level reporting (arithmetic, log-transformed^47^ or even geometric^36^ means or medians), but also in the adopted Lp(a) cut-offs for risk assessment, defined as 1-SD increase,^11,33^ 10-mg/dL increase^16^ or in a percentile-based manner,^27,31^ apart from the more typical 30 or 50 mg/dL thresholds.

### 4.3 Underlying molecular mechanisms

A central role in controlling the circulatory levels of Lp(a) plays its size, determined by its apo(a) isoform with a highly variable number of KIV_2_ repeats.^5^ Fewer KIV_2_ repeats reduce the apo(a) size and lead to higher Lp(a) concentrations.^23,31,32^ Furthermore, many genetic factors and, most importantly, SNPs in the *LPA* locus, such as rs10455872 and rs3798220, affect Lp(a) concentration and increase Lp(a)-related CAVD risk.^20,29,31–33,41^ Ancestry- and race/ethnicity-based studies have revealed, not only the highly variable pattern of SNPs and KIV_2_ repeats among different subgroups, but also that such variations can have diverging effects in different populations.^74,76^ Although our understanding of the Lp(a) detrimental effect on the aortic valve is not complete, converging data indicate a pleiotropic mechanism of action.^3^ Apart from its typical atherogenic property, originating from its lipid-carrying nature, Lp(a) seems to exert its primary effect through the delivery of oxidised phospholipids (OxPL) directly to valve leaflets.^62,74^ When stress is induced to valvular endothelium, hydrophilic Lp(a) molecules infiltrate endothelial cells, attract and act on monocytes, smooth muscle and interstitial cells, and trigger pro-inflammatory and pro-calcifying reactions, mainly through OxPL, which is converted to lysophosphatidic acid through ATX.^12,15,16,19,32,39,51,62^

### 4.4 Treatment strategies

Several treatment alternatives for lowering circulating Lp(a) levels have been proposed. While statins seem to slightly raise Lp(a) levels,^8,74^ niacin, cholesteryl ester transfer protein inhibitors (i.e. Anacetrapid) and mipomersen have been shown to reduce Lp(a) levels by 20–30%.^77^ The same figure for monoclonal antibodies against the PCSK9 seems even higher. In the FOURIER (Further Cardiovascular Outcomes Research with PCSK9 Inhibition in Subjects with Elevated Risk) trial, evolocumab significantly reduced Lp(a) levels and reduced the atherosclerotic cardiovascular risk.^78^ Similar data were obtained from the ODYSSEY OUTCOMES trial for alirocumab.^79^ Recent evidence suggests an apo(a) size-related manner in achieving Lp(a) level reduction, with larger molecules leading to higher reductions (and further 3% reduction for each additional kringle).^80^ Despite their Lp(a) lowering effect, none of these approaches have proved their clinical benefit specifically for CAVD, so far.^81,82^ Elucidating answers are expected from the ongoing prospective, double-blind, randomised phase II clinical trial ‘Effect of PCSK9 InhibitorS On Calcific Aortic Valve DiseasE’ (NCT04968509), the results of which have not been published so far. Other approaches shift the interest from lowering Lp(a) to targeting its CAVD-inducing mechanism. An OxPL neutralising antibody, E06-single chain variable fragment, has also been proposed, showing a significant reduction in aortic valve pressure gradient in mice, yet with no clinical data from human studies.^62^

Finally, the recently developed concept of inhibiting apo(a) messenger RNA production with antisense oligonucleotides comes with promising preliminary results. Pelacarsen (TQJ230), the first developed drug of this category, achieved up to 80% reduction of Lp(a), when used weekly.^83^ The ongoing HORIZON clinical trial (NCT04023552), which explores the effect of pelacarsen on cardiovascular events, will further enlighten this field. Furthermore, the GalNAc-conjugated siRNAs olpasiran and SLN360, which reduce Lp(a) by directly targeting the *LPA* gene, have also denoted optimistic data.^84^ Both drugs demonstrated a safe profile,^85,86^ while large prospective, double-blind, clinical trials (NCT04270760, NCT05537571) have already been approved and are expected to yield results by 2024.

### 4.5 Study limitations

The findings of our work can be better understood within its limitations. Diagnostic bias between groups of individual studies, mainly owed to incomplete reporting of hosted data, sporadically encountered in some of them, cannot be excluded. Although this risk seems limited due to the design and data balance of most included studies, the outcomes of this analysis should be interpreted with caution minding this factor. Although the multi-level heterogeneity imposed challenges in summarising the individual study outcomes and, primarily, in pooling estimates, we opted for wider inclusion criteria and included even diverging study designs, so as to provide a holistic view of the available evidence. Accordingly, pooling a standardised mean difference to account for different measurement units and other variations, produced a result that strongly captures the direction of association, but can hardly be physically interpreted. To address this, we repeated the analysis for the more coherent subdivision of studies that reported Lp(a) levels in nmol/L, and obtained a more meaningful mean difference of 22.63 nmol/L for between-group Lp(a) levels, yet based on a smaller sample than the original pool of more than 25 000 subjects.

## 5. Conclusions

The meta-analysis of existing evidence implies an active role for Lp(a) in the initiation and progression of CAVD, with increased mortality and risk for serious adverse outcomes associated with higher Lp(a) levels. Systematic review of the literature revealed that Lp(a) induces additional risk for T1DM, BAV and heterozygous FH populations, while its levels and effect vary across different race/ethnic groups. Moreover, the quantitative analysis showed a more potent association for male-prevalent populations, as well as for younger individuals, suggesting an early and distinct role of Lp(a) in the initiation of the disease, when other risk factors and degenerative lesions are absent. Of note, genetic variations of *LPA* gene loci are also implicated in the risk of AVS. Further research could shed light upon populations at risk and pave the way for acknowledging more actively the role of Lp(a) in the disease, along with establishing treatment strategies.

## Supplementary Material

cvad062_Supplementary_Data
