# Supplementary material for: Lipoprotein(a) and calcific aortic valve disease initiation and progression: a systematic review and meta-analysis
Source: Cardiovasc Res. 2023 Apr 20;119(8):1641–55. doi: 10.1093/cvr/cvad062 (PMC10702855; doi:10.1093/cvr/cvad062)
Supplement: cvad062_Supplementary_Data [file cvad062_supplementary_data.pdf]

Lipoprotein(a) and calcific aortic valve disease initiation and progression: a systematic review and meta-analysis

Supplementary material online

Authors

Panteleimon Pantelidis*[1], Evangelos Oikonomou*[1], Stamatios Lampsas*[1], Georgios E. Zakynthinos[1], Antonios Lysandrou[1], Konstantinos Kalogeras[1], Efstratios Katsianos[1], Panagiotis Theofilis[1], Gerasimos Siasos[1,2], Michael Andrew Vavuranakis[1], Alexios S. Antonopoulos[3], Dimitris Tousoulis[4], Manolis Vavouranakis[1]

* *Equally contributed*

Affiliations

1: 3rd Department of Cardiology, National and Kapodistrian University of Athens, Medical School, Sotiria Chest Disease Hospital, Athens, Greece.

2: Cardiovascular Division, Brigham and Women's Hospital, Harvard Medical School, Boston, MA, USA.

3: Clinical, Experimental Surgery & Translational Research Center, Biomedical Research Foundation of the Academy of Athens, Athens, Greece.

4: National and Kapodistrian University of Athens, Medical School, Athens, Greece

Corresponding author

Dr Evangelos Oikonomou MD, MSc, PhD

3rd Department of Cardiology, Athens Chest Hospital "Sotiria",

National and Kapodistrian University of Athens, Medical School, Athens, Greece

e-mail: boikono@gmail.com; tel: +306947701299

Appendix **S1**. Search queries

Search query regarding the association between Lp(a) levels and CAVD:

Database	*Query*	*Results*
PubMed	("Lipoprotein(a)"[Mesh:NoExp] OR (lipoprotein a) OR "lpa") AND ("Aortic Valve Stenosis"[Mesh:NoExp] OR (aortic valve stenosis) OR "AV stenosis" OR (aortic valve calcification) OR "AV calcification" OR (aortic valve disease) OR "AV disease" OR (calcific aortic valve) OR (degenerative aortic valve))	277
Embase	('lipoprotein a'/exp OR 'lipoprotein a' OR 'lpa') AND ('aortic valve stenosis'/syn OR 'aortic valve stenosis' OR 'av stenosis' OR 'aortic valve calcification' OR 'av calcification' OR 'aortic valve disease' OR 'av disease' OR 'calcific aortic valve' OR 'degenerative aortic valve')	425
Scopus	(TITLE-ABS-KEY): (("lipoprotein a" OR "lipoprotein-a" OR "lipoprotein(a)" OR lpa) AND ("aortic valve stenosis" OR "av stenosis" OR "aortic valve calcification" OR "av calcification" OR "aortic valve disease" OR "av disease" OR "calcific aortic valve" OR "degenerative aortic valve"))	331
Web of Science	("lipoprotein a" OR "lipoprotein-a" OR "lipoprotein(a)" OR lpa) AND ("aortic valve stenosis" OR "av stenosis" OR "aortic valve calcification" OR "av calcification" OR "aortic valve disease" OR "av disease" OR "calcific aortic valve" OR "degenerative aortic valve")	276
ScienceDirect	Title, abstract, keywords: ("lipoprotein a" OR lpa) AND ("aortic valve stenosis" OR "aortic valve calcification" OR "aortic valve disease" OR "av disease" OR "calcific aortic valve" OR "degenerative aortic valve")	91
Cochrane Library	("lipoprotein a" OR "lipoprotein-a" OR "lipoprotein(a)" OR lpa) AND ("aortic valve stenosis" OR "av stenosis" OR "aortic valve calcification" OR "av calcification" OR "aortic valve disease" OR "av disease" OR "calcific aortic valve" OR "degenerative aortic valve") in Title Abstract Keyword	25
OpenGrey	"lipoprotein a" (due to sparsity of the topic in grey literature)	31
LILACS	("lipoprotein a" OR "lipoprotein a" OR "lipoprotein a" OR lpa) AND ("aortic valve stenosis" OR "av stenosis" OR "aortic valve calcification" OR "av calcification" OR "aortic valve disease" OR "av disease")	4

Search query regarding the association between Lp(a)-related genetic risk factors and CAVD:

Database	*Query*	*Results*
PubMed	("Genetic Variation"[Mesh:NoExp] OR "genetic variation" OR "gene?" OR "genetic" OR "SNP?" OR "polymorphism?" OR "allele?") AND ("Lipoprotein(a)"[Mesh:NoExp] OR (lipoprotein a) OR "lpa") AND ("Aortic Valve Stenosis"[Mesh:NoExp] OR (aortic valve stenosis) OR "AV stenosis" OR (aortic valve calcification) OR "AV calcification" OR (aortic valve disease) OR "AV disease" OR (calcific aortic valve) OR (degenerative aortic valve))	107
Embase	('genetic variation' OR 'gene' OR 'SNP') AND ('lipoprotein a'/exp OR 'lipoprotein a' OR 'lpa') AND ('aortic valve stenosis'/syn OR 'aortic valve stenosis' OR 'aortic valve calcification' OR 'aortic valve disease')	168
Scopus	TITLE-ABS-KEY (("genetic variation" OR "gene?" OR "genetic" OR "SNP?" OR "polymorphism?" OR "allele?") AND ("lipoprotein a" OR "lipoprotein-a" OR "lipoprotein(a)" OR lpa) AND ("aortic valve stenosis" OR "av stenosis" OR "aortic valve calcification" OR "av calcification" OR "aortic valve disease" OR "av disease" OR "calcific aortic valve" OR "degenerative aortic valve"))	133
Web of Science	("genetic variation" OR "gene?" OR "genetic" OR "SNP?" OR "polymorphism?" OR "allele?") AND ("lipoprotein a" OR "lipoprotein-a" OR "lipoprotein(a)" OR lpa) AND ("aortic valve stenosis" OR "av stenosis" OR "aortic valve calcification" OR "av calcification" OR "aortic valve disease" OR "av disease" OR "calcific aortic valve" OR "degenerative aortic valve")	97
ScienceDirect	Title, abstract, keywords: ("gene?" OR "genetic" OR "polymorphism?" OR "allele?") AND ("lipoprotein a" OR lpa) AND ("aortic valve stenosis" OR "aortic valve calcification" OR "aortic valve disease")	37
Cochrane Library	("genetic variation" OR "gene?" OR "genetic" OR "SNP?" OR "polymorphism?" OR "allele?") AND ("lipoprotein a" OR "lipoprotein-a" OR "lipoprotein(a)" OR lpa) AND ("aortic valve stenosis" OR "av stenosis" OR "aortic valve calcification" OR "av calcification" OR "aortic valve disease" OR "av disease" OR "calcific aortic valve" OR "degenerative aortic valve") in Title Abstract Keyword	17

Appendix **S2**. Detailed description of methods

Objectives

Primary objectives

Qualitative synthesis:

i. To identify, summarise and report evidence found in literature, concerning the association between lipoprotein(a) [Lp(a)] levels and aortic valve calcification (AVC) and stenosis (AVS).

Quantitative synthesis:

ii. To test the null hypothesis of no difference in Lp(a) levels between AVS and non-AVS subjects.

iii. To test the null hypothesis of no difference in Lp(a) levels between AVS and non-AVS subjects, only for studies reporting the molar concentration of Lp(a) in nmol/L (sensitivity analysis).

iv. To test the null hypothesis of no difference in AVS progression rate (estimated with annualised peak aortic velocity change, in m/s/year) between individuals with low and those with high Lp(a) levels.

v. To test the null hypothesis of no increased risk of AVS-related, serious adverse outcomes (including death, aortic valve replacement and AVS-related hospitalisation) between individuals with low and those with high Lp(a) levels.

Secondary objectives

Qualitative synthesis:

vi. To identify and report variations of the above relationship in special populations (with comorbidities/ chronic illnesses) and different races/ethnicities.

vii. To identify, summarise and report evidence found in literature, concerning the association between Lp(a)-related genetic risk factors and AVC/AVS.

Quantitative synthesis:

viii. To test whether there is a significant difference in Lp(a) levels with respect to AVS severity.

ix. To test whether age and sex affect the difference in Lp(a) levels between AVS and non-AVS subjects (meta-regression analysis).
x. To test whether age and sex affect the risk of AVS-related, serious adverse events between individuals with low and those with high Lp(a) levels (meta-regression analysis).
xi. To test the null hypothesis of no increased risk of AVS for certain *LPA* single nucleotide polymorphisms (SNPs, rs10455872 and rs3798220), and also test whether this association is affected by age and sex (meta-regression analysis).

Search strategy and selection criteria

Indexing databases searched and corresponding search queries: See Appendix S1

Selection criteria for qualitative synthesis:

- Types of studies: Observational studies (cohort, registry-based cohort, case-control and cross-sectional), with original data published in peer-reviewed journals
- Language: English
- PECO requirements:
  - Participants: General population or special population groups (with comorbidities / chronic illnesses)
  - Exposure: High soluble plasma levels of Lp(a)
  - Comparator: Normal/low soluble plasma levels of Lp(a)
  - Outcomes: AVC or AVS and related outcomes
- Additional data from studies exploring the association between relevant genetic risk factors (*LPA* SNPs and KIV_2 repeats) and CAVD, were also retrieved.

Selection criteria for quantitative synthesis:

All applied to qualitative synthesis, plus:

- Only case-control or cross-sectional studies (for objectives ii, iii, viii, ix, xi) and only cohort studies (for objectives iv, v, x, xi).
- Exclusion of studies concerning only special populations (with comorbidities).
- Exclusion of studies not reporting soluble plasma Lp(a) concentrations as continuous distributions (for objectives ii, iii, viii, ix)
- Exclusion of studies only reporting outcomes other than AVS (e.g. solely AVC) (for objectives ii, iii, viii, ix, xi), studies not reporting the AVS progression rate

differences in terms of annualised peak aortic velocity change (for objective iv), and studies not reporting risk measures for AVS-related, serious adverse outcomes, including death (for objectives v, x).

- Exclusion of not age-matched studies with considerable differences between the compared groups.

Data extraction

The following data were extracted:

- First author's name
- Year of publication and title
- Study type / design
- Sample size (overall and per group)
- Originating population (country, setting, race/ethnicity and any baseline characteristics / comorbidities)
- Age and sex for the whole sample and each subgroup included
- Lp(a) level differences for the compared groups, in case groups are formed according to AVC or AVS outcomes (mostly case-control and cross-sectional studies)
- Risk estimates [risk (RR), odds (OR) or hazard (HR) ratio] for AVC, AVS and related outcomes (crude or adjusted), in case groups are formed according to Lp(a) levels
- Risk estimates (RR, HR or OR) for AVC and AVS, with respect to Lp(a)-related genetic risk factors (rs10455872, rs3798220 and other SNPs, KIV_2 repeats)
- Kit and method used for measuring Lp(a), along with the measurement unit (mg/dL or nmol/L)
- The exact outcome at hand and its ascertainment method / diagnostic modality
- Follow-up time (depending on study design)
- Any reported gene variants related to Lp(a) concentration or contributing to the outcomes at hand
- Matched variables (if matching was performed)
- Information regarding the risk of bias (in line with the NOS criteria)

Quality assessment

The Newcastle-Ottawa Quality Assessment Scale (NOS) tool for evaluating the risk of bias in non-randomised studies, was used for cohort and case-control studies[1], with an adapted version for cross-sectional ones.[2] NOS assessment is based on three main domains: (i) The selection of participants, (ii) the comparability of the groups and (iii) the ascertainment of either the exposure or outcome of interest. The maximum score is 9 for cohort and case-control and 10 for cross-sectional studies. We assigned scores of 0–3, 4–6, and 7–9 (or 7–10 for cross-sectional design) for low, moderate and high quality, respectively. For overall and domain-specific score results, see Appendix S3.

Statistical analysis

Descriptive analysis and qualitative synthesis

Data were tabularised and summarised into evidence tables (see Appendix S4), with the use of spreadsheets. In case of missing data, we contacted the corresponding study authors with a request to provide further information. Any relevant differences were reported unchanged in their means ± standard deviations (SD) or medians [interquartile ranges (IQR)], depending on the original study. Any risk estimates, crude or adjusted RR, OR, HR, were reported as found. Data were summarised and descriptively reported for the sample size, the study type, demographics (age and sex), outcome of interest, country / cohort of origin and NOS score.

Quantitative synthesis

- The pooled standardised mean difference (SMD) of Lp(a) levels (measured in nmol/L or mg/dL, on a continuous scale), between AVS and non-AVS subjects, was calculated.
- A sensitivity analysis was performed, including only studies reporting the molar concentration of Lp(a) in nmol/L, and calculating the pooled mean difference (MD) of Lp(a) (measured in nmol/L) between AVS and non-AVS patients.
- The pooled SMD of Lp(a) levels was calculated (a) between severe- and mild/moderate-AVS patients, and (b) between severe- and mild-AVS ones.
- The pooled OR of AVS was calculated for carriers of minor (risk) alleles of the following *LPA* SNPs: rs10455872 allele G, rs3798220 allele C.

- The pooled MD of peak aortic velocity change (in m/s/year) was calculated between low- and high-Lp(a) individuals.
- The pooled HR of serious adverse outcomes (death, aortic valve replacement and AVS-related hospitalisation) was calculated for low- vs. high-Lp(a) individuals.

In case means and SDs were missing, they were estimated from their corresponding medians and IQRs, according to Luo et al.[3] and Wan et al.[4] ORs were approximated by RRs, under the "rare disease" assumption (objective xi).[5] Random-effects models were used, with the inverse variance method and the Knapp-Hartung adjustment[6] for calculating the 95% confidence interval (CI) around the pooled effect. Between-study heterogeneity was assessed with Higgins' and Thompson's I^2 statistic.[7] In case of high heterogeneity, outlying and highly influential studies were spotted (by leave-one-out method),[8] and the analysis was repeated after excluding them. Forest plots were provided for all outcomes. Publication bias was evaluated with Egger's test[9] and visualised with contour-enhanced funnel plots.

- Meta-regression was also performed to investigate the effect of mean age and sex on the Lp(a) level difference between AVS and non-AVS populations, on the risk of serious adverse events between high- and low-Lp(a) populations and on the risk of AVS between carriers and non-carriers of relevant *LPA* SNPs minor alleles. In case total sample values were missing, they were estimated from the corresponding subgroups as the weighted means. Maximum-likelihood was used as the τ^2 estimator and the Knapp-Hartung method for the regression coefficients. Bubble plots were generated for all predictors.

The significance threshold was set to 0.05. All statistical analyses were performed in R version 4.2.0,[10] with the use of *meta*, *metafor* and *dmetar* libraries.[11–13]

Appendix **S3**. Quality assessment results with the Newcastle-Ottawa Quality Assessment Scale (NOS) tool

Study	*Design*	*Selection*	*Comparability*	*Exposure/ Outcome*	*Score*	*Quality*
Arsenault et al.[14]	Case-control	***	**	*	6/9	Moderate
Boakye et al.[15]	Case-control	***	*	***	7/9	High
Bortnick et al.[16]	Cross-sectional	***	**	***	8/10	High
Bourgeois et al.[17]	Case-control	***	*	***	7/9	High
Bozbas et al.[18]	Cross-sectional	**	*	***	6/10	Moderate
Cao et al.[19]	Cross-sectional	****	**	***	9/10	High
Capoulade et al.[20]	Cohort	****	*	***	8/9	High
Capoulade et al.[21]	Cohort	***	**	***	8/9	High
Capoulade et al.[22]	Cohort	***	*	**	6/9	Moderate
Chen et al.[23]	Cross-sectional	***	*	***	7/10	High
Despres et al.[24]	Case-control	***	**	***	8/9	High
Dong et al.[25]	Case-control	**	*	***	6/9	Moderate
Glader et al.[26]	Case-control	****	*	***	8/9	High
Gotoh et al.[27]	Cross-sectional	***	*	***	7/10	High
Gudbjartsson et al.[28]	Case-control	****	**	***	9/9	High
Hojo et al.[29]	Case-control	****	**	**	8/9	High

Hovland et al.[30]	Cross-sectional	****	*	***	8/10	High
Kaiser et al.[31]	Case-control	***	**	**	7/9	High
Kaiser et al.[32]	Cross-sectional	***	**	***	8/10	High
Kaiser et al.[33]	Cohort	****	*	**	7/9	High
Kaltoft et al.[34]	Cohort	***	**	**	7/9	High
Kaltoft et al.[35]	Cohort	***	*	***	7/9	High
Kamstrup et al.[36]	Cohort	***	**	**	7/9	High
Kamstrup et al.[37]	Case-control	****	*	**	7/9	High
Langsted et al.[38]	Cross-sectional	**	**	**	6/10	Moderate
Littmann et al.[39]	Cross-sectional	***	*	**	6/10	Moderate
Liu et al.[40]	Cohort	**	*	***	6/9	Moderate
Ljungberg et al.[41]	Case-control	****	**	***	9/9	High
Mahabadi et al.[42]	Case-control	***	*	***	7/9	High
Makshood et al.[43]	Cross-sectional	***	**	**	7/10	High
Nsaibia et al.[44]	Case-control	***	**	***	8/9	High
Obisesan et al.[45]	Cohort	***	*	***	7/9	High
Ozkan et al.[46]	Case-control	***	*	*	5/9	Moderate
Simony et al.[47]	Case-control	****	**	***	9/9	High
Stewart et al.[48]	Case-control	***	**	*	6/9	Moderate
Sticchi et al.[49]	Cross-sectional	***	**	*	6/10	Moderate
Vassiliou et al.[50]	Case-control	****	*	**	7/9	High

Vongpromek et al.[51]	Cross-sectional	****	*	***	8/10	High
Wang et al.[52]	Cross-sectional	****	**	***	9/10	High
Wang et al.[53]	Cross-sectional	**	*	***	6/10	Moderate
Wilkinson et al.[54]	Case-control	****	**	***	9/9	High
Wodaje et al.[55]	Cohort	****	*	**	7/9	High
Zheng et al.[56]	Case-control	****	**	**	8/9	High
Zheng et al.[57]	Cohort	****	**	***	9/9	High

Case-Control
Selection (max 4 stars), Comparability (max 2 stars), Exposure (max 3 stars), Score (max score 9)

Cross Sectional
Selection (max 5 stars, Comparability (max 2 stars), Outcome (max 3 stars), Score (max score 10)

Cohort
Selection (max 4 stars), Comparability (max 2 stars), Outcome (max 3 stars), Score (max score 9)

Quality
low: 0-3, moderate: 4-6 and high: 7-9 (or 7-10)

Appendix **S4**. Included studies

Detailed flowchart for study selection

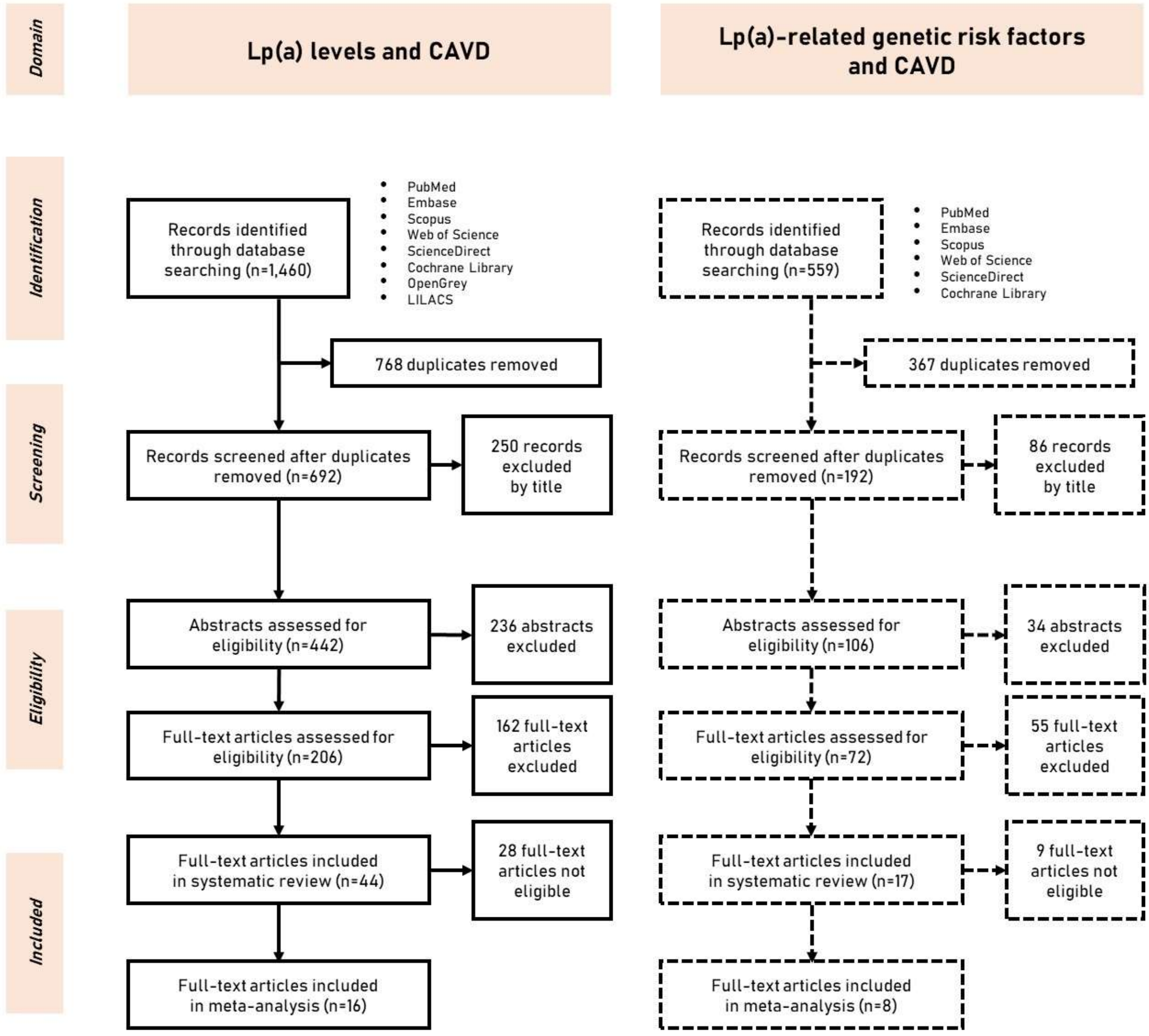

Geographical distribution of studies and study populations concerning Lp(a) levels and CAVD

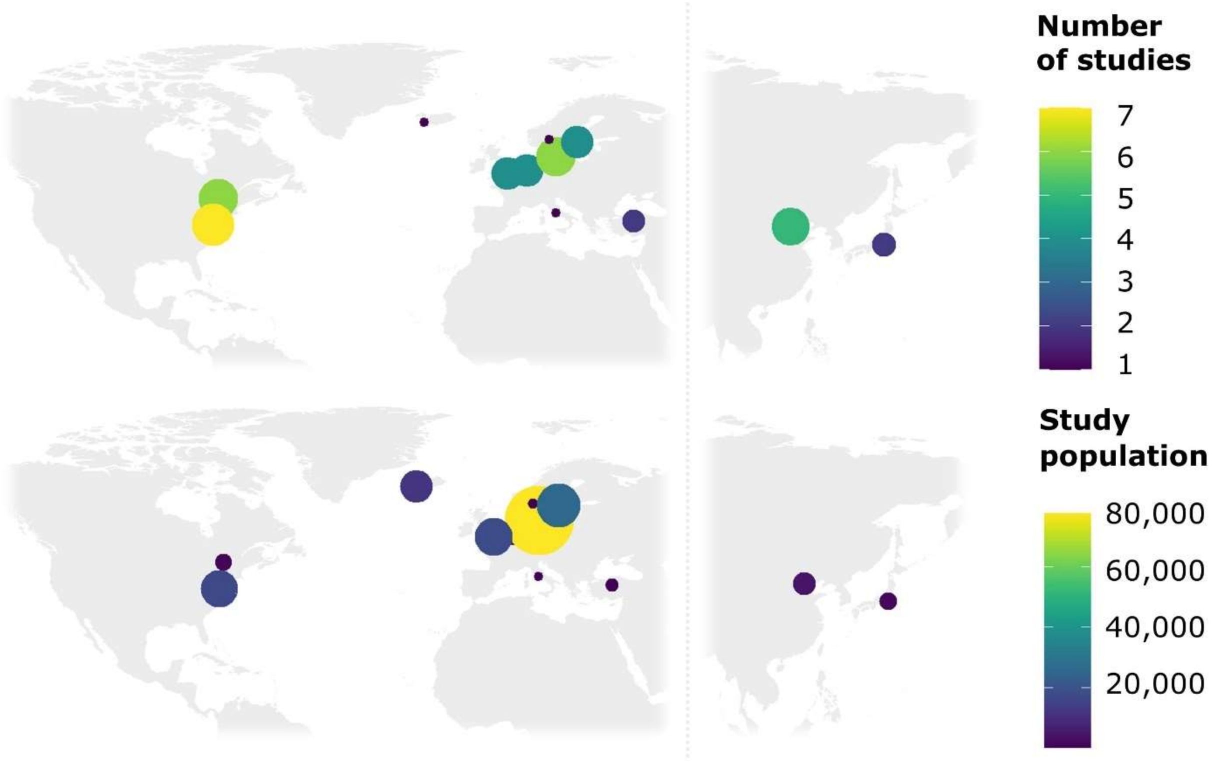

Country	*Number of studies*
USA	7
Canada	6
Denmark	6
China	5
UK	4
Sweden	4
Netherlands	4
Turkey	2
Japan	2
Iceland	1
Norway	1
Germany	1
Italy	1

Country	*Percent of population under study*
Denmark	47.62%
Sweden	16.13%
UK	11.07%
USA	10.32%
Iceland	7.44%
China	2.58%
Netherlands	2.12%
Japan	1.01%
Canada	<1%
Germany	<1%
Turkey	<1%
Norway	<1%
Italy	<1%

Evidence table of studies regarding Lp(a) levels and CAVD

Study	*Design*	*Country*	*Study population*	*Sample size*	*Age (yrs)*	*Sex (female)*	*Compared groups (extended)*	*Outcome ascertainment method*	*Lp(a) measurement unit and kit*	*Key Findings (extended)*
Arsenault et al.[14]	Case-control	UK	General population from the EPIC-Norfolk study	17,553	59.1 (n/a)	56%	AVS (n=118) vs. non-AVS (n=17,435)	Hospitalization or death due to AVS (ICD10: I35)	mg/dL, Denka Seiken (Coventry, UK)	▪ AVS pts display higher Lp(a) levels [16.2 (6.3-44.5) vs. 11.6 (6.2-27.6) mg/dL; p=0.025, unadjusted].<br>▪ Pts with Lp(a)≥50 mg/dL have a nearly 2-fold risk for AVS after adjusting for sex, age, smoking and LDLc.
Boakye et al.[15]	Case-control	USA	General population from the ARIC study	2,283	80.5±4.3	61.4%	AVC (n=1,022) vs. non-AVC (n=1,261)	CT	mg/dL, Denka Seiken (Tokyo, Japan)	▪ AVC was present in 44.8% of participants, with higher rates among older, White males (58.2%).<br>▪ Lp(a) was higher among Black participants [30.6; 95% CI: 16.2-53.1 vs. 9.5; 95% CI: 4.1-26.7; p<0.001].<br>▪ Lp(a) was independently associated with AVC [adjusted prevalence ratio: 1.09; 95% CI: 1.04-1.15; p=0.001], along with other risk factors.
Bortnick et al.[16]	Cross-sectional	USA	General population of the CHS	3,426	72±5	63%	AVC (n=2,027) vs. non-AVC (n=1,399)	Echo for calcification and stenosis (peak aortic velocity >2.0 m/s)	mg/dL, Genentech (San Francisco, California, USA)	▪ AVC pts had higher Lp(a) levels (55.5±52.1 mg/dL) than those without calcification (48.9±46.4 mg/dL), even after adjustment (aRR per 1-SD increase of Lp(a) levels: 1.05; 95% CI: 1.02-1.08).<br>▪ No association was found for aortic or mitral annulus calcification.
Bourgeois et al.[17]	Case-control	Canada	Pts with CAVS from the QHLI; plus controls	232	64.8±9	42.7 %	CAVS (n=88) vs. non-CAVS (n=144)	Echo (ACC/AHA criteria)	nmol/L, Tina-quant Lipoprotein(a) Gen. 2 system, Cobas integra 400/800, Roche (Mannheim, Germany)	▪ Borderline difference in Lp(a) between CAVS and non-CAVS (89.6±119.0 vs. 63.3±87.9 nmol/L; p=0.055).<br>▪ Circulating ATX, bound to Apo(a) particles of Lp(a), was found significantly increased in CAVS pts (aOR: 2.80; 95% CI: 1.39-5.66; p=0.003).
Bozbas et al.[18]	Cross-sectional	Turkey	Pts from the Department of Cardiology, Baskent University Hospital, Turkey	285	70.2±7.3	67.3%	AVC (n=112) vs. non-AVC (n=173)	Echo (AV sclerosis with peak aortic velocity ≤2.5 m/s)	mg/dL, Roche diagnostics (Mannheim, Germany)	▪ AVC associated with higher levels of Lp(a) (27.4; range: 13.0-47.5 vs. 19.9; range: 10.7-36.1 mg/dL; p=0.033).<br>▪ CRP levels were also higher in AVC pts. The associations with Lp(a) and CRP were retained in multifactorial analysis.
Cao et al.[19]	Cross-sectional	USA	Ethnic populations from the MESA (n=4,679) cohort (Whites, Hispanics, Chinese Americans, Blacks)	4,593	Range for age medians: 61 - 62, and IQR: 17 - 18	Range: 51.4 - 61.2%	Association of Lp(a) mass [Lp(a)-M], cholesterol content [Lp(a)-C], and particle concentration [Lp(a)-P] with CAVD	CT (Aortic valve calcium score)	mg/dL, Denka Seiken (Tokyo, Japan)	▪ CAVD was significantly associated with increased Lp(a)-M, -C and -P, either at their upper 25th cut-off (aRR range: 1.48-1.49; p<0.001, for all associations) or at their upper 15th cut-off (aRR range: 1.49-1.54; p<0.001, for all associations), after adjusting for age, sex, race/ethnicity, hypertension, smoking, LDLc and other factors.<br>▪ Blacks had higher Lp(a)-M, -C and -P levels than other populations, while Whites and Hispanics showed higher AVC rates.<br>▪ The same relationships hold for Lp(a) and CAD.
Capoulade et al.[20]	Cohort	Canada	Pts with mild/moderate AVS from the ASTRONOMER trial	220	58±13	40%	Association of Lp(a) and OxPL-apoB with AVS progression	Echo, endpoint of AV replacement / cardiac death	mg/dL, Kit used in ASTRONOMER trial	▪ AVS progression was faster for the top tertiles of Lp(a) (peak aortic velocity: +0.26±0.26 vs.+0.17±0.21 m/s/year; p=0.005) and OxPL-apoB (+0.26±0.26 m/s/year vs. +0.17±0.21

							within the cohort			▪ m/s/year; p=0.01), during 3.5±1.2 yrs of follow-up.<br>▪ Pts in the top tertiles of Lp(a) or OxPL-apoB were at increased risk of AV replacement and cardiac death, after adjusting for age, sex and baseline AVS severity.
Capoulade et al.[21]	Cohort	Canada	Pts with mild/moderate AVS from the ASTRONOMER trial	220	58±13	40%	Association of Lp(a) with the outcomes	Echo	mg/dL, Kit used in ASTRONOMER trial	▪ CAVS progression (+0.2m/s/year in peak aortic velocity) was associated with the levels of Lp(a) (OR per 10-mg/dL increase: 1.10; 95% CI 1.03-1.19; p=0.006), as well as with OxPL-apoB and OxPL-apo(a), after 3.5 (2.9-4.5) yrs of follow-up.<br>▪ For younger ages (≤57 yrs, n=108), this association was stronger (OR per 10-mg/dL increase: 1.19; 95% CI: 1.07-1.33; p=0.002).<br>▪ The association was also retained after adjusting for age, sex, hypertension, smoking history, metabolic syndrome, SBP, statin use, LDLc levels, and other factors.
Capoulade et al.[22]	Cohort	Canada	Pts with mild/moderate AVS from the ASTRONOMER trial	218	58±13	40%	Association of Lp(a) different Lp(a) levels (cut-off: top tertile) with the outcomes within the cohort	Echo, endpoint of AV replacement / cardiac death	mg/dL, Kit used in ASTRONOMER trial	▪ High Lp(a) pts had significantly higher progression rate of AVS (quantified with annualised peak aortic velocity change) and risk of AV replacement / cardiac death, over a median 3.5 (2.9-4.5) yrs of follow-up.
Chen et al.[23]	Cross-sectional	China	General population from the PRECISE study	3,067	61.1±7.5	53.4%	CAVS (n=270) vs. non-CAVS (n=2,797)	Echo	mg/dL, n/a	▪ Lp(a) was higher in CAVS pts [9.1 (3.7-24.8) vs. 6.1 (3.2-14.3) mg/dL; p<0.001], with a multivariably-adjusted aOR: 1.002 (95% CI: 1.001-1.002; p<0.001).
Despres et al.[24]	Case-control	Canada	Pts with CAVS; plus controls	496	68.2 (n/a)	38.36%	CAVS (n=388) vs. non-CAVS (n=108)	Echo to assess AVS severity; PET to assess microcalcification and CT (Ca-Score) for calcification	nmol/L, Tina-quant Lp(a) Gen. 2, Cobas integra 400/800, Roche diagnostics (Mannheim, Germany)	▪ Higher Lp(a) in CAVS pts [28.7 (8.2-116.6) vs. 10.9 (3.6-28.8) nmol/L; p<0.0001). Similarly, CAVS pts had higher OxPL-apoB and -apo(a) (p<0.0001 for both).<br>▪ Pts with no CAVS, but elevated Lp(a) appeared with 40% higher TBR_{mean} (^{18}FNaF PET uptake) than those with lower Lp(a) (1.25±0.23 vs. 1.15±0.11; p=0.02, Lp(a) cut-off: 75 nmol/L).
Dong et al.[25]	Case-control	China	Pts from the Department of Cardiology, Tianjin Chest Hospital, China	219	63.72 (n/a)	53.5%	CAVD (n=71) vs. CAD (n=77) vs. non-CAVD/CAD (n=78)	Echo (peak aortic velocity >2.5 m/s)	nmol/L, Roche diagnostics (Mannheim, Germany)	▪ Lp(a) levels were significantly higher in the CAVD [37.2 (16.5-79.6) nmol/L] and the CAD [46.7 (21.5-104.6) nmol/L] groups, as compared to controls [23.6 (9.4-48.6) nmol/L; p<0.001].<br>▪ Three *LPA* SNPs (rs6415084, rs3798221, rs7770628) affected Lp(a) levels.
Glader et al.[26]	Case-control	Sweden	Pts with significant AVS undergoing AV replacement from the University Hospital in Umea, Sweden; plus controls	202	71.3±8.5	40.6%	Significant AVS (n=101) vs. non-AVS matched controls (n=101)	Echo	mg/dL, Biopool AB (Umea, Sweden)	▪ High Lp(a) (≥48mg/dL) pts in greater risk for AVS requiring intervention (aOR: 3.4; 95% CI: 1.1-11.2, adjusted for several risk factors).<br>▪ Significant associations were also found with leptin, t-PA and a C. Pneumoniae - specific IgG titre, with a synergy in action between the latter and Lp(a).

Gotoh et al.[27]	Cross-sectional	Japan	General population from the JMS-CECS	784	62±11	55.7%	High (≥30 mg/dL, n=180) vs. low (<30 mg/dL, n=604) Lp(a)	Echo to assess AVC	mg/dL, Biopool (Umea, Sweden)	▪ Pts with Lp(a) ≥30mg/dL were at greater risk for AVC as compared to those with Lp(a) <30mg/dL (36.1% vs. 12.7%; p<0.001).<br>▪ Lp(a) levels were higher in women (p<0.01) and were not associated with age. The mean difference in Lp(a) between AVC and non-AVC pts, was higher in the female subpopulation (16.1mg/dL) than in the male one (13mg/dL).
Gudbjartsson et al.[28]	Case-control	Iceland	Pts medical records from Landspitali (National University Hospital of Iceland) or from death registries	143,087 in total / 12,137 with Lp(a) levels	n/a	n/a	Association of Lp(a) levels and KIV_2 genotypes with AVS within the cohort	Medical records with diagnostic codes ICD9 and ICD10	nmol/L, Tina-quant Lipoprotein(a) Gen. 2, Cobas c311, Roche (Basel, Switzerland)	▪ AVS is associated with higher Lp(a) molar concentrations (aOR: 1.17 per 50nmol/L increase in Lp(a); 95% CI: 1.12-1.22; p<0.0001).<br>▪ Fewer KIV_2 repeats lead to higher Lp(a) molar concentrations and larger apo(a) isoform size. Carriers of G4925A (KIV_2 mutation) have both small apo(a) and lower Lp(a) concentrations.<br>▪ KIV_2 repeats are associated with CVD, only before adjusting for Lp(a) molar concentration.
Hojo et al.[29]	Case-control	Japan	Pts with PAD from Kitakanto Cardiovascular Hospital, Japan	861	73 (66-78)	20.9%	Association of Lp(a) / other factors with AVS and/or other VHD within the cohort	Echo (ACC/AHA and ASE criteria)	mg/dL, n/a	▪ AVS associated with higher Lp(a) levels [34.0 (16.7-50.0) vs. 20.0 (11.0-35.0) mg/dL; p=0.002].<br>▪ Also, MVS and combined AVS and/or MVS pts had elevated Lp(a) levels [37.0 (21.5-77.3) vs. 21.0 (11.0-35.0); p=0.037 and 34.0 (17.3-50.0) vs. 20.0 (11.0-35.0); p=0.001, respectively].<br>▪ Lp(a) levels correlated with LDLc and high-sensitivity CRP levels (p<0.05).
Hovland et al.[30]	Cross-sectional	Norway	Pts with heterozygous FH, from the outpatient Lipid Clinic, Oslo University Hospital, Norway	78	49±15 for FH pts; 44±12 for controls	55% for FH pts; 57% for controls	FH pts (n=64; 29 with high and 35 with low Lp(a), cut-off: 75nmol/L) vs. controls (n=14)	Echo to assess AVS	nmol/L, Roche diagnostics; mg/dL, N latex Lp(a) on Behring Nephlometer-II, Dade Behring	▪ Lp(a) levels were similar for FH and control pts, measured both in mass (p=0.12) and molarity (p=0.10), along with age (p=0.21) and sex (p=1).<br>▪ Aortic valve area and peak aortic velocity did not differ between high- and low-Lp(a) FH pts (p=0.3 and p=0.65, respectively).<br>▪ FH pts had significantly higher peak aortic velocity and smaller aortic valve area than controls [1.2 (1.1-1.5) vs. 1.0 (1.0-1.1) m/s; p=0.02, and 2.5±0.6 vs. 2.8±0.6 cm^2; p=0.04].
Kaiser et al.[31]	Case-control	Netherlands	Pts with mild to moderate AVS (peak aortic velocity: 2-4m/s) from the Amsterdam University Medical Centers and Cardiologie Centra Nederland	58	66.4±5.6	15.4%	High (n=26) vs. low (n=26) Lp(a) groups (cut-off: 50 mg/dL)	^{18}FNaF PET to assess calcification	mg/dL, Randox Laboratories (UK)	▪ No significant difference between high- and low-Lp(a) groups, in AV calcium score [1,388 (450-2,424) vs. 1,173 (927-1,628) AU, respectively; p=0.839] or ^{18}FNaF PET uptake (TBR_{mean}: 3.02±1.26 vs. 3.05±0.96, respectively; p=0.902).
Kaiser et al.[32]	Cross-sectional	Netherlands	General population from the population-based Rotterdam Study & Amsterdam UMC study	3,271	63.3±13.3	53%	Association of AVC with Lp(a) and other factors within the cohort	CT	mg/dL, Cobas 8000, Randox Laboratories (UK)	▪ Lp(a) between 80th and 94th percentile (47.7-88.7mg/dL) almost doubles the risk for AVC (aOR: 1.89; 95% CI: 1.48-2.42; p<0.001), while values ≥95th percentile (>88.7mg/dL), almost triple it (aOR: 2.84; 95% CI: 1.96-4.10; p<0.001).<br>▪ This association holds for both the Rotterdam Study cohort and the Amsterdam UMC cohort with

										younger subjects (aOR: 1.53; 95% CI: 1.34-1.73 and aOR: 2.09; 95% CI: 1.23-3.55, for every 50mg/dL increase in Lp(a), respectively).
Kaiser et al.[33]	Cohort	Netherlands	General population from the Rotterdam Study	922	66±4.2	52.3%	Three groups according to AVC status at baseline and follow-up: -/- (n=287), -/+ (n=415), +/+ (n=220)	CT	mg/dL, Randox laboatories (UK)	▪ Lp(a) levels were associated with baseline and new-onset AVC [aOR: 1.43; 95% CI: 1.15–1.79 and aOR: 1.30; 95% CI: 1.02–1.65, respectively, for each 50 mg/dL increase in Lp(a).<br>▪ Lp(a) was not associated with AVC progression (β: −71 AU; 95% CI: −117 to 35; for each 50 mg/dL of Lp(a) increase).
Kaltoft et al.[34]	Cohort	Denmark	General population from the CGPS	12,006 with CT / 85,884 in total	59.2 (51.1-67) for the sub-cohort with CT	57%	Association of Lp(a) and *LPA* genotypes with AVC and AVS	CT for calcification and medical records with diagnostic codes ICD8: 424.10, 424.12, 424.18, 424.19, or ICD10: I35.0, I35.2	mg/dL, DiaSys, Diagnostic Systems or Denka Seiken	▪ Elevated Lp(a) increased the risk for AVC (aOR: 1.62; 95% CI: 1.48-1.77, for 10-fold increase in Lp(a) levels). The corresponding risk for AVS was aHR: 1.54; 95% CI: 1.38-1.71.<br>▪ KIV_2 repeats and rs10455872 were associated with increased calcification (aOR: 2.23; 95% CI: 1.81-2.76, and aOR: 1.86; 95% CI: 1.64-2.13, respectively).<br>▪ 31% (95% CI:16%, 76%) of the AVS effect was mediated through calcification.
Kaltoft et al.[35]	Cohort	Denmark	General population from the CGPS	69,988	60 (range: 20-100)	54%	Lp(a) levels-based (Low, ≤9 mg/dL, n= 34,989; moderate, 10-68 mg/dL, n=28,001; high, ≥69 mg/dL, n=6,998)	Medical records with diagnostic codes ICD8: 424.10, 424.12, 424.18, 424.19, or ICD10: I35.0, I35.2	mg/dL, Denka or assay from DiaSys Diagnostic Systems	▪ Moderate-Lp(a) and high-Lp(a) showed increased risk for CAVD, as compared to low-Lp(a) ones, over a ~7.4 yrs period (aHR: 1.28; 95% CI: 1.13-1.44 and aHR: 1.86; 95% CI: 1.57-2.21, respectively).<br>▪ For pts in the same Lp(a) percentile interval, men were at a higher 10-year risk for CAVD than women.<br>▪ Similarly; pts in the top 10% of BMI, had an aHR: 1.8; 95% CI: 1.5-2.1, for CAVD, while the corresponding risk for those in the 10% of both BMI and Lp(a), was aHR: 3.5; 95% CI: 2.5-5.1.
Kamstrup et al.[36]	Cohort	Denmark	General population from the CCHS and the CGPS	77,680	58 (47-67)	56%	Risk of AVS for different percentile intervals of Lp(a) and *LPA* genotypes	Medical records with diagnostic codes ICD8: 424.10, 424.12, 424.18, 424.19, or ICD10: I35.0, I35.2	mg/dL, Three assays, all converted to Denka Seiken	▪ Lp(a) was associated with increased risk for AVS. The adjusted HR for AVS, corresponding to each Lp(a) percentile interval, were: 1.2 (95% CI: 0.8-1.7) for 22nd to 66th (5-19 mg/dl); 1.6 (95% CI: 1.1-2.4) for 67th to 89th (20-64 mg/dl); 2.0 (95% CI: 1.2-3.4) for 90th to 95th (65-90 mg/dl); 2.9 (95% CI: 1.8-4.9) for >95th (>90 mg/dl); as compared to the risk, for levels <22nd percentile (<5 mg/dl; p<0.001).<br>▪ Higher Lp(a) levels and AVS risk were associated with rs10455872 and rs3798220 minor alleles, and low KIV_2 repeats number (p<0.05).
Kamstrup et al.[37]	Case-control	Denmark	General population from the CGPS	2,138	74 (67-79)	37%	CAVD (n=725) vs. non-CAVD (n=1,413)	Medical records with diagnostic codes ICD8: 424.10, 424.12, 424.18, 424.19, or ICD10: I35.0, I35.2	mg/dL, DiaSys Diagnostic Systems or Denka Seiken	▪ Lp(a) higher in CAVD group [12 (4-48) vs. 8 (4-24) mg/dL; p<0.001].<br>▪ For every 10mg/dL-increase in Lp(a) the multivariable-adjusted odds ratio for CAVD is 1.10 (95% CI: 1.06-1.13).<br>▪ OxPL-apoB and OxPL-apo(a) correlated strongly with Lp(a), among both cases and controls, and also, both raised the risk for CAVD.<br>▪ rs10455872 and KIV_2 repeats explained 39% of Lp(a) variation.
Langsted et al.[38]	Cross-sectional	Denmark	General population	100,578 in total;	58 (48-68)	54.3%	Association of Lp(a) and	Medical records with	Lp(a) cholesterol	▪ For every 1-SD increase in Lp(a) there was a multifactorially

			from the CCHS and the CGPS	52,652 with Lp(a) levels			*LPA* genotypes with AVS, MI and plasma levels of CRP within the cohort	diagnostic codes ICD8: 424.10, 424.12, 424.18, 424.19, or ICD10: I35.0, I35.2	content in mg/dL, Three assays, all converted to Denka Seiken	adjusted aHR: 1.23 (95% CI: 1.06-1.41) for AVS, and an aHR: 1.20 (95% CI: 1.10-1.31) for MI.<br>▪ *LPA* SNPs (rs10455872, rs3798220) and KIV_2 repeats were also significantly associated with both AVS and MI (aHR range: 1.18-1.38).<br>▪ CRP increased by 29% (95% CI: 23-34) for every 50-mg/dL increase in Lp(a) (or by 21%; 95% CI: 16-25, when adjusted for age, sex, smoking, hypertension, use of lipid-lowering therapy, and menopausal status). *LPA* SNPs and the KIV_2 repeats were not associated with higher CRP levels.
Littmann et al.[39]	Cross-sectional	Sweden	T1DM pts from outpatient Endocrinology Unit, Karolinska University Hospital	1,860	48±16	44%	Four pts groups according to Lp(a) levels (very low <10, n=621; low 10-30, n=502; intermediate 30-120, n=434; high >120 nmol/L, n=303)	CAVD defined as: a) Echo showing stenosis or sclerosis, or b) ICD10: I35.0 or I35.2 on medical record	nmol/L, Roche diagnostics	▪ CAVD prevalence range: 2.6% ("very low" group) to 7.3% ("high" group)<br>▪ T1DM pts with increased Lp(a) levels are in higher risk for developing CAVD (aRR=2.03; p<0.05, when Lp(a) >120 nmol/L)
Liu et al.[40]	Cohort	China	Chinese pts with CAVS (mild/moderate stenosis) from Fuwai hospital (Beijing, China)	652 at baseline (cross-sectional handling) / 359 with follow-up (after excluding severe AVS)	62±17	41.7%	Pts at lower 1st and 2nd tertiles of Lp(a) (≤ 38.15 mg/dL, n=417) vs. higher 3rd tertile (>38.15 mg/dL, n=235)	Echo for baseline assessment; Composite endpoint after follow-up: Unplanned AV replacement / death from AVS	mg/dL, LASAY Lp(a) auto, SHIMA laboratories (Tokyo, Japan)	▪ At baseline; pts with higher Lp(a) were older, with more comorbidities, and also demonstrated a higher peak aortic velocity (3.70±1.12 vs. 3.43±1.14 m/s; p=0.012) and higher rates of severe AVS (aOR: 1.78; 95% CI: 1.18-2.66; p=0.006).<br>▪ After 3.16±2.74 yrs of follow-up (excluding severe AVS cases), Lp(a) (aHR: 0.73; 95% CI: 0.43-1.24; p=0.239) and all other factors, except age (aHR: 1.4; 95% CI: 1.04-1.88; p=0.024) were not significant predictors of the endpoint.
Ljungberg et al.[41]	Case-control	Sweden	Pts undergoing surgery for AVS; previously enrolled to one of the following three cohorts: VIP, MONICA, MSP; plus controls	1,007 in total; 955 (95%) with Lp(a) levels	56.7 at baseline (when Lp(a) was measured); ~67 at the time of surgery (for the cases)	48%	AVS pts requiring surgery (n=308; 254 with severe AVS) vs. matched non-AVS controls (n=647)*	Echo	nmol/L, Lp(a) Gen. 2, 05852625190, Roche diagnostics (Basel, Switzerland)	▪ AVS pts showed higher Lp(a) levels [55.6 (47.2-64.0) vs. 40.1 (35.6-44.7) nmol/L; p=0.005]<br>▪ Severe AVS cases displayed lower Lp(a) values [52.9 (44.4-61.4) vs. 77.8 (47.7-107.0) for mild/moderate AVS].<br>▪ In subgroup analysis, with respect to CAD, this association was retained for pts diagnosed with CAD (p<0.001, n=569), but not for the CAD negative group (p=0.69, n=383).
Mahabadi et al.[42]	Case-control	Germany	Pts ≥70 yrs of age with severe AVS, admitted to the West German Heart and Vascular Center for TAVI; plus controls	968	80±5	48%	Severe AVS (n=484) vs. non-AVS (n=484)	Echo (Mean pressure gradient ≥40mmHg or aortic valve area <1,0cm²)	mg/dL, Lp(a)-antigen / Lp(a)-antibody reaction, Siemens Healthcare Advia system	▪ Median Lp(a) did not differ between severe-AVS and non-AVS pts [17 (8-56) vs. 18.5 (8.5-57) mg/dL; p=0.56). The outcome was retained in a risk factor-adjusted multifactorial model (aOR: 0.98; 95% CI: 0.90-1.06; p=0.57).
Makshood et al.[43]	Cross-sectional	USA	Ethnic populations from the MASALA (n=695) and MESA	5,366	Range for age mean: 59.3 - 62.4, and SD: 9.2 - 10.4	Range: 51.4 - 57.0%	Ethnic groups (South Asians, n=695 vs. Whites, n=1,705 vs.	CT (Aortic valve calcium score)	mg/dL, Denka Seiken (Tokyo, Japan)	▪ S. Asians have higher median Lp(a) levels (17.0mg/dL) than all other ethnic groups (12.9-13.1 mg/dL), except Blacks (35.1 mg/dL).<br>▪ AVC prevalence was lower in Chinese (6.6%), similar to Blacks (11.7%) and higher in Whites (14.6%)

			(n=4,671) cohorts				Hispanics, n=1,063 vs. Chinese Americans, n=558 vs. Blacks, n=1,345)			and Hispanics (13.2%), as compared to S. Asians (10.7%).<br>▪ No associations between Lp(a) and AVC in S. Asians or other groups, except Blacks and Whites (positive association).
Nsaibia et al.[44]	Case-control	Canada	CAD pts with or without CAVS	300	71±9	35%	CAVS & CAD (n=150) vs. only CAD pts (n=150)	Echo	mg/dL, Tina-quant Lipoprotein(a) Gen. 2, Cobas integra 400/800, Roche diagnostics (Laval, QC, Canada)	▪ Pts with CAD showed higher levels of Lp(a) when CAVS was present (32.5±36.2 vs. 23.7±29.5 mg/dL; p=0.003).<br>▪ Lp(a) levels ≥50 mg/dL increased the risk of CAVS only through ATX mass and activity [unadjusted OR: 2.06 (95% CI: 1.11-3.82); p=0.02; while aOR: 2.24 (95% CI: 0.64-7.76); p=0.20 in multifactorial model including ATX mass (p=0.001) and activity (p=0.007) as significant predictors].
Obisesan et al.[45]	Cohort	USA	General population from the ARIC study	2,083	59.2±4.3	62.2%	Low (≤50 mg/dL, n=1,691) vs. high (>50 mg/dL, n=392) Lp(a) levels	CT	mg/dL, Denka Seiken (Tokyo, Japan)	▪ Lp(a) levels >50mg/dL increased the risk for AVC [aOR: 1.79; 95% CI: 1.32-2.43, adjusted for age, race, sex, education level, smoking status, alcohol drinking status, cardiometabolic risk factors and lipi-lowering therapy].<br>▪ Race or sex did not significantly affect this relationship.
Ozkan et al.[46]	Case-control	Turkey	CAVD pts; plus controls (aged over 60)	152	72.2 (n/a)	50.7%	CAVD (n=75; 31 with severe, 28 with moderate, 16 with mild AVS) vs. non-CAVD (n=77)	Echo (ACC/AHA criteria) to identify CAVD and assess AVS severity	mg/dL, LS-F21752, LSBio LifeSpan BioSciences (USA)	▪ CAVD pts had higher Lp(a) levels [68.67 (67.17-70.16) vs. 27.05 (25.86-28.23) mg/dL; p<0.001].<br>▪ Milder AVS severity corresponded to higher Lp(a) levels, although with no statistical significance [mild: 70.28 (68.34-72.23) vs. moderate: 69.10 (67.30-70.90) vs. severe: 67.44 (64.26-70.62); p=0.388].<br>▪ Two *LPA* SNPs (rs1055872, rs3798220) were associated with CAVD.
Simony et al.[47]	Case-control	Denmark	General population from the CGPS	70,042	60 (50-69)	53.6%	Risk of AVS / other CVD for high Lp(a) levels (>40mg/dL or 83nmol/L) in 32,497 men and 37,545 women	Medical records with diagnostic codes ICD8: 424.10, 424.12, 424.18, 424.19, or ICD10: I35.0, I35.2	mg/dL and nmol/L, DiaSys, Denka Seiken (Tokyo, Japan) and Denka Seiken (Roche, Rotkreuz, Switzerland)	▪ In women, Lp(a) levels increased by 27% after menopause and decreased by 12% with hormone replacement therapy.<br>▪ In multivariably adjusted models, Lp(a) levels >40 mg/dL were independently associated with AVS, MI, CAD for both sexes (p<0.05 for all calculated risk measures) and heart failure only for men, but not with CVD and all-cause mortality.
Stewart et al.[48]	Case-control	USA	General population of the CHS, (≥65 yrs of age, from four USA communitie s)	5,114	72.7 (n/a)	57.9%	CAVD (sclerosis or stenosis, n=1,405) vs. non-CAVD (n=3,709)	Echo (sclerosis or peak aortic velocity ≥2.5 m/s)	mg/dL, n/a	▪ CAVD group had significantly higher Lp(a) levels (62.3±71.4 vs. 50.7±48.5 mg/dL; p<0.001, unadjusted).<br>▪ Lp(a) is strongly associated with CAVD (aOR: 1.23; 95% CI: 1.14-1.32; p<0.001).<br>▪ Age and male sex double the risk for CAVD, while positive associations have been found for hypertension, smoking and LDLc levels.
Sticchi et al.[49]	Cross-sectional	Italy	BAV pts from the CCD, Referring Center for Marfan syndrome or related disorders, AOU	69	45 (30-53)	20.3%	Group formation based on AV calcification (no, n=39; mild/moder ate, n=26; severe, n=4) and stenosis	Echo	mg/dL, Randox (Antrim, UK)	▪ In BAV pts, higher Lp(a) levels are associated with the degree of AV calcification (severe: 560, mild/moderate: 134, no: 78 mg/dL; p=0.008) and stenosis (stenosis: 214, no stenosis: 104 mg/dL; p=0.043).

			Careggi				(no, n=55; stenosis, n=14)			
Vassiliou et al.[50]	Case-control	UK	Subcohort of pts with AVS from study with identifier NCT00930735 (CT.gov); plus controls	165	75.3 (n/a)	29.7%	AVS (n=110; 75 with severe and 35 with mild/moderate) vs. non-AVS (n=55)	CMR to assess AVS severity (ACC/AHA criteria)	mg/dL, Lp(a) Ultra, Sentinel Diagnostics	▪ AVS pts had higher Lp(a) levels [30.9 (7.5-68.8) vs. 10.0 (4.1-26.6) mg/dL; p<0.001].<br>▪ Severe AVS pts had (non-significantly) lower Lp(a) levels than those with mild/moderate [24.2 (7.2-70.0) vs. 38.4 (9.1-65.6) mg/dL; p=0.64].<br>▪ Lp(a) was not associated with myocardial fibrosis in CMR.
Vongpromek et al.[51]	Cross-sectional	Netherlands	Pts with heterozygous FH, familial hypercholesterolaemia	129	51±8	37.2%	Association of AVC with Lp(a) and other factors within the cohort	CT	mg/dL, 171399910930, Diasys Diagnostic System (Germany)	▪ AVC present in 38.8% of pts (Ca-Score>0).<br>▪ Lp(a) concentration was a significant predictor of AVC (aOR per 10-mg/dL increase: 1.11; 95% CI 1.01-1.20; p=0.03).
Wang et al.[52]	Cross-sectional	China	Symptomatic pts (chest pain/tightness) admitted to Tianjin Chest Hospital, China	152	70	43.4%	AVC-based (no calcification, n=40; mildly calcified, n=32; moderately calcified, n=48; heavily calcified, n=32)	Echo and CT to assess calcification	nmol/L, Roche diagnostics (Mannheim, Germany)	▪ Lp(a) is associated with the grade of calcification (1.21±0.30, heavily-; 1.41±0.32, moderately-; 1.61±0.34, mildly-; 1.63±0.38, no-calcified; p<0.01, log-transformed data in nmol/L).<br>▪ In multifactorial analysis, Lp(a) was significantly associated with AVC (aOR: 1.04; 95% CI: 1.01-1.06; p=0.005), along with PCSK9 (aOR: 1.01) and age (aOR: 1.12).
Wang et al.[53]	Cross-sectional	China	Pts with newly-diagnosed MI, at Zhongda Hospital, China	410	58.6±10.8	14.1%	AVC (n=182) vs. non-AVC (n=228)	Echo	mg/dL, n/a	▪ Lp(a) was higher in AVC pts [23.2 (11.1-42.5) vs. 15.4 (6.8-30.8) mg/dL; p<0.001] with new-onset MI.<br>▪ This relationship was independent from age, sex, diabetes and hypertension.<br>▪ Lp(a) levels were associated with CAVS in a non-linear fashion.
Wilkinson et al.[54]	Case-control	USA	General population from echo lab at University of California, San Diego Medical Center	4,079	75 (n/a)	47%	CAVS (n=2,710) vs. non-CAVS (n=1,369)	Echo (ACC/AHA and ASE criteria)	mg/dL, ARUP laboratories (Salt Lake City, Utah)	▪ Low rates of Lp(a) measurement (4.6% for the CAVS and 3.1% for the non-CAVS group).<br>▪ Similar Lp(a) levels for the two subgroups (with measurements), with CAVS: 14 (6-48) vs. non-CAVS: 15.5 (6.5-63) mg/dL; p=0.734.
Wodaje et al.[55]	Cohort	Sweden	General population from the Stockholm County cohort	23,298	55.5±17.1	52%	CAVS (n=489) vs. non-CAVS (n=22,909)	Medical records with diagnostic codes ICD10: I35.0, I35.2	nmol/L and mg/dL, Tina-quant Lipoprotein(a) Gen. 1 and 2 (Roche)	▪ CAVS pts had higher Lp(a) levels [20.2 (7.6-63.7) vs. 17 (6.6-43.6) mg/dL; p=0.009; n=19,151 or 22.4 (7.8-127.9) vs. 19.5 (7.8-83) nmol/L; p=0.23; n=4,247].<br>▪ This difference was retained for both sexes.<br>▪ Lp(a) levels >90th percentile increased the risk for AVS (aHR: 1.53; 95% CI: 1.08-2.15; p=0.016; age, sex adjusted), as compared with levels between 0-50th percentiles.<br>▪ The age/sex-adjusted risk for intervention was aHR: 1.42 (95% CI: 0.73-2.79; p=0.304).
Zheng et al.[56]	Case-control	UK	General population from the EPIC-Norfolk study	17,745	59.2±9.1	55.1%	AVS (n=403) vs. non-AVS (n=17,342)	Hospitalization or death due to AVS (ICD10: I35)	mg/dL, Denka Seiken (Coventry, UK)	▪ AVS pts presented significantly higher levels of Lp(a) [15.3 (7.0-41.7) vs. 11.7 (6.3-27.7) mg/dL; p<0.001, unadjusted].<br>▪ Lp(a) >50 mg/dL is an independent risk factor for AVS (aHR: 1.70; 95% CI: 1.33-2.19; p<0.001; age, sex, LDLc, CAD adjusted).

Zheng et al.[57]	Cohort	UK	Pts with AVS (peak aortic velocity >2 m/s) from the Ring of Fire study and the SALTIRE study	145	70.3±9.9	31.7%	Low (≤35 mg/dL, n=96) vs. high (>35 mg/dL, n=49) Lp(a) levels	PET at baseline; Echo, CT, documented medical events at baseline and during follow-up	mg/dL, n/a	▪ At baseline, pts at the top Lp(a) tertile (>35 mg/dL) had increased calcification, measured with ^{18}FNaF PET uptake (TBR_{mean}: 2.16 vs. 1.97; p=0.043), but did not differ in peak aortic velocity (p=0.150) or Ca-Score (p=0.429). This also holds for OxPL-apoB.<br>▪ During follow-up (CT after 2 yrs, echo after 1-3 yrs, clinical outcome after 5 yrs), high-Lp(a) showed increased Ca-Score progression [309 (142-483) vs. 93 (56-296) AU/year; p=0.015], faster hemodynamic progression on echo (0.23±0.20 vs. 0.14±0.20 m/s/year; p=0.019), and increased risk for aortic valve replacement and death (HR: 1.87; 95% CI: 1.13-3.08; p=0.014).

All values in mean±SD or median (IQR); *, Information obtained after contacting the authors; ^{18}FNaF PET, 18F-Sodium Fluoride Positron Emission Tomography; 95% CI, 95% Confidence interval; ACC/AHA, American College of Cardiology/American Heart Association; aHR, Adjusted hazard ratio; aOR, Adjusted odds ratio; ApoCIII, Apolipoprotein C-III; ARIC, Atherosclerosis Risk In Communities study; aRR, Adjusted relative risk; ASE, American Society of Echocardiography; ASTRONOMER, Aortic Stenosis Progression Observation: Measuring Effects of Rosuvastatin trial; ATX, Autotaxin; AU, Agatston units; AV, Aortic valve; AVC, Aortic valve calcification; AVS, Aortic valve stenosis; BAV, Bicuspid aortic valve; Ca-Score, Calcium score; CAD, Coronary artery disease; CAVD, Calcific aortic valve disease; CAVS, Calcific aortic valve stenosis; CCD, Center for Cardiovascular Diagnosis; CCHS, Copenhagen City Heart Study; CGPS, Copenhagen General Population Study; CHS, Cardiovascular Health Study; CMR, Cardiac magnetic resonance; CRP, C-reactive protein; CT, Computed tomography; CVD, Cardiovascular disease; Echo, Echocardiography; ELISA, Enzyme-linked immunosorbent assay; EPIC, European Prospective Investigation into Cancer study; FH, Familial hypercholesterolemia; ICD8/9/10, International Classification of Diseases - 8th/9th/10th Revision; JMS-CECS, Jichi Medical School-Cardiac Echo and Cohort Study; KIV_2, Kringle IV-2 repeat; LDLc, Low density lipoprotein cholesterol; Lp(a), Lipoprotein(a); LPA, Lipoprotein(a) protein coding gene; MASALA, Mediators of Atherosclerosis in South Asians Living in America study; MESA, Multi-Ethnic Study of Atherosclerosis; MI, Myocardial infarction; MONICA, Northern Sweden Monitoring of Trends and Determinants in Cardiovascular Disease; MSP, Mammary Screening Program; MVS, Mitral valve stenosis; n, Number; n/a, Not available; OxPL-apo(a)/-apoB, Oxidized phospholipids bound to apolipoprotein(a) / apolipoprotein B; PAD; Peripheral arterial disease; PRECISE, PolyvasculaR Evaluation for Cognitive Impairment and vaScular Events study; Pts; Patients; QHLI, Quebec Heart and Lung Institute; SALTIRE, Scottish Aortic Stenosis and Lipid Lowering Trial, Impact on Regression; SBP, Systolic blood pressure; SD, Standard deviation; SNPs, Single nucleotide polymorphisms; T1DM, Type 1 diabetes mellitus; TAVI, Transcatheter aortic valve implantation; TBR_{mean}, Mean tissue-to-background ratio; UK, United Kingdom; USA, United States of America; VHD, Valvular heart disease; VIP, Vasterbotten Intervention Programme; yrs, Years

Evidence table of studies regarding Lp(a)-related genetic risk factors and CAVD

Study	*Population**	*LPA SNPs***	*Key Findings*
Arsenault et al.[14]	EPIC-Norfolk (14,735), MHI Biobank (763)	rs10455872 (+)	▪ In EPIC-Norfolk, pts heterozygous or homozygous for rs10455872 minor allele (G) had an increased, dose-dependent risk for AVS (HR: 1.78; 95% CI: 1.11-2.87 and HR: 4.83; 95% CI: 1.77-13.20, respectively).<br>▪ A similar trend was observed when adjusting for Lp(a) levels (HR: 1.56; 95% CI: 0.90-2.70 for heterozygous and HR: 3.83; 95% CI: 1.26-11.65 for homozygous).<br>▪ The result was replicated in MHI Biobank (OR: 1.57; 95% CI: 1.10-2.26 for rs10455872).
Cairns et al.[58]	CCHS, CGPS, EPIC-Norfolk, UK Biobank, MDCS, MHI Biobank (combined: 133,729 for rs10455872; 89,293 for rs3798220)	rs10455872 (+), rs3798220 (+)	▪ Both SNPs raise the risk for AVS (RR: 1.66; 95% CI: 1.48-1.86; $p=2\times10^{-18}$ and RR: 1.43; 95% CI: 1.04-1.99; $p=3\times10^{-2}$; for rs10455872 and rs3798220, respectively).
Cardoso-Saldaña et al.[59]	Mexican cohort (1,265)	rs10455872 (+), rs7765803 (+), rs6907156 (+), rs1321195 (-), rs12212807 (-), rs6919346 (-)	▪ Only rs10455872 was associated with AVC (p=0.013)<br>▪ Three SNPs were associated with higher Lp(a) levels [rs10455872(G) with p=0.013, rs6907156(T) with p=0.021 and rs7765803(G) p=0.001].
Chen et al.[60]	GERA (44,703)	rs10455872 (+), rs3798220 (+)	▪ Greater risk of AVS for rs10455872 (OR: 1.34; 95% CI: 1.23-1.47; $p=1.7\times10^{-10}$) and rs3798220 (OR: 1.31; 95% CI: 1.09-1.58; $p=3.6\times10^{-3}$).<br>▪ Dose-dependent relationship (2-fold risk for homozygotes in any of the two SNPs risk alleles).<br>▪ For rs10455872 the OR declined with increasing age (p=0.03)
Dong et al.[25]	Han Chinese cohort (142)	rs10455872 (-), rs3798221 (-), rs6415084 (-), rs7770628 (-)	▪ All SNPs were associated with higher Lp(a) levels (p<0.01, for all comparisons), however no significant association with CAVD was observed.
Emdin et al.[61]	UK Biobank (112,338)	rs10455872, rs3798220, rs41272114, rs143431368: combined genetic risk (+)	▪ Genetically lowered Lp(a) levels associated with lower risk for AVS (OR: 0.63; 95% CI: 0.47-0.83; p=0.001, per 1-SD genetically determined decrease in Lp(a) levels).
Gudbjartsson et al.[28]	Icelandic cohort (143,454)	KIV_2 repeats (+)	▪ KIV_2 repeats increase the risk for AVS (OR: 1.09; 95% CI: 1.04-1.14; p<0.001), but not independently from Lp(a) levels (OR: 0.98; 95% CI: 0.93-1.04; p=0.47, when adjusted to Lp(a) levels).
Helgadottir et al.[62]	Icelandic cohort, UK Biobank, MDCS, HUNT, CHIP, cohort from Stockholm (combined: 806,325)	rs10455872 (+)	▪ rs10455872 raises the risk for AVS (OR: 1.46; 95% CI: 1.37-1.56; $p=1.9\times10^{-31}$).
Junco-Vicente et al.[63]	Spanish cohort (653)	rs10455872 (-)	▪ No significant increase in AVS risk for rs10455872 (OR: 1.31; 95% CI: 0.8-2.22), with similar results for tricuspid and bicuspid AV pts.
Kaltoft et al.[34]	CGPS (12,006)	rs10455872 (+), rs3798220 (+), KIV_2 repeats (+)	▪ Higher AVC risk associated with rs10455872 (OR: 1.86; 95% CI: 1.64-2.13), rs3798220 (OR: 1.52; 95% CI: 1.13-2.04) and fewer KIV_2 repeats (OR: 2.23; 95% CI: 1.81-2.76, for ≤23 vs. ≥36 repeats).<br>▪ Genetically determined Lp(a) levels associated with greater risk for AVS (HR: 2.13; 95% CI: 1.85-2.46).
Kamstrup et al.[36]	CCHS, CGPS (combined: 77,680)	rs10455872 (+), rs3798220 (-), KIV_2 repeats (+)	▪ Increase in AVS risk for rs10455872 (HR: 1.6; 95% CI: 1.2-2.0), but not for rs3798220 (HR: 1.0; 95% CI: 0.5-1.8).<br>▪ Fewer KIV_2 repeats increase the risk for AVS in a dose-dependent way.<br>▪ Minor alleles and fewer KIV_2 repeats are not independent, however they independently increase Lp(a) levels (rs10455872 explained 28% of Lp(a) level variation, rs3798220 5% and KIV_2 repeats 24%; combined: 41%).<br>▪ Genetically determined Lp(a) levels increase AVS risk (HR: 1.6; 95% CI: 1.2-2.0).
Kamstrup et al.[37]	CGPS (2,138)	rs10455872 (+), KIV_2 repeats (+)	▪ Genetically determined Lp(a) associated with higher CAVD risk, with RR: 1.11; 95% CI: 1.06-1.15 for rs10455872 and RR: 1.08; 95% CI: 1.02-1.13 for KIV_2 repeats (RR: 1.09; 95% CI: 1.05-1.14 when combined – age- and sex-matched CAVD pts and controls at 1:2).
Ozkan et al.[64]	Turkish cohort (152)	rs10455872 (-), rs3798220 (-)	▪ Minor alleles of both SNPs significantly less frequent in CAVD patients (1.33% vs. 68.2% for G allele of rs10455872; 0% vs. 59% for C allele of rs3798220).
Perrot et al.[65]	QUEBEC-CAVS, EPIC-Norfolk, UK Biobank, CAVS-France 1, CAVS-France 2, CAVS-France 3, GERA (combined: 438,181)	rs10455872, rs3798220, rs41272114: combined genetic risk (+)	▪ Genetically elevated Lp(a) associated with higher risk of CAVS (OR: 1.49; 95% CI: 1.32-1.68; p<0.001)<br>▪ Similar risk for CAD and non-CAD pts.<br>▪ First-degree relatives found with higher risk for AV microcalcification.

Sticchi et al.[49]	Cohorts of pts with bicuspid aortic valve and controls (138)	KIV_2 repeats (-)	▪ KIV_2 repeat number inversely correlated with Lp(a) levels (r=−0.219; p=0.01).<br>▪ Fewer KIV_2 repeats for higher degrees of AVC, although not statistically significant associated [no calcification: median: 19 (IQR: 13-30); mild/moderate: 14 (11-33); severe: 12.5 (12-13); p=0.102].
Thanassoulis et al.[66]	FHS, AGES-RS, MESA (combined: 6,942); CCHS, MDCS, HNR (combined: 7,687)	rs10455872 (+)	▪ rs10455872 (OR: 2.05; $p=9\times10^{-10}$) and genetically predicted Lp(a) levels were associated with AVC.<br>▪ *LPA* genotype increased risk for AVS (HR: 1.68; 95% CI: 1.32-2.15) and AV replacement (HR: 1.54; 95% CI: 1.05-2.27).
Trenkwalder et al.[67]	GeneCAST (12,882)	rs10455872 (+)	▪ rs10455872 increased the risk of AVS (OR: 1.37; 95% CI: 1.24-1.52; $p=6.9\times10^{-10}$), larger effect size in non-CAD pts

* Included cohorts and their corresponding numbers of patients (in parentheses); ** Referring to minor alleles (G allele for rs10455872, C allele for rs3798220); For SNPs, "+" indicates positive association, while "-" no significant association (except for Ozkan et al. where there is an inverse association); For KIV_2 repeats, "+" indicates inverse association (fewer repeats associated with higher risk), while "-" no significant association; 95% CI, 95% Confidence interval; AGES-RS, Age, Gene/Environment Susceptibility–Reykjavik Study; AV, Aortic valve; AVC, AV calcification; AVS, AV stenosis; CAD, Coronary artery disease; CAVD, Calcific aortic valve disease; CAVS-France 1/2/3, CAVS-France 1, 2 or 3 cohorts; CCHS, Copenhagen City Heart Study; CGPS, Copenhagen General Population Survey; CHIP, Cardiovascular Health Improvement Project; EPIC-Norfolk, European Prospective Investigation of Cancer–Norfolk study; FHS, Framingham Heart Study; GeneCAST, Genetics of Calcific Aortic Stenosis cohort; GERA, Genetic Epidemiology Research on Aging cohort; HNR, Heinz Nixdorf Recall Study; HR, Hazard ratio; HUNT, Norwegian Nord-Trøndelag Health Study; IQR, Interquartile range; KIV_2, Kringle IV-2 repeat; Lp(a), Lipoprotein(a); LPA, Lipoprotein(a) protein coding gene; MDCS, Malmö Diet and Cancer Study; MESA, Multi-Ethnic Study of Atherosclerosis; MHI Biobank, Montreal Heart Institute Biobank; OR, Odds ratio; pts, Patients; QUEBEC-CAVS, Calcific Aortic Valve Stenosis in Quebec study; RR, Risk ratio; SD, Standard deviation; SNPs, Single nucleotide polymorphisms; UK Biobank, United Kingdom Biobank

Appendix **S5**. Detailed outcomes of the meta-analysis

Lp(a) difference between AVS and non-AVS individuals

A total of 12 studies were initially included (n=26,343 individuals; 8.7% with AVS; age$_{\text{weighted-mean}}$: 58.7 years; females$_{\text{weighted-mean}}$: 50.4%). Initial analysis showed no difference in the Lp(a) levels between AVS and non-AVS patients, with an SMD of 2.77 (95% CI: -2.69 to 8.22), along with high heterogeneity among studies (I^2=96.8%).

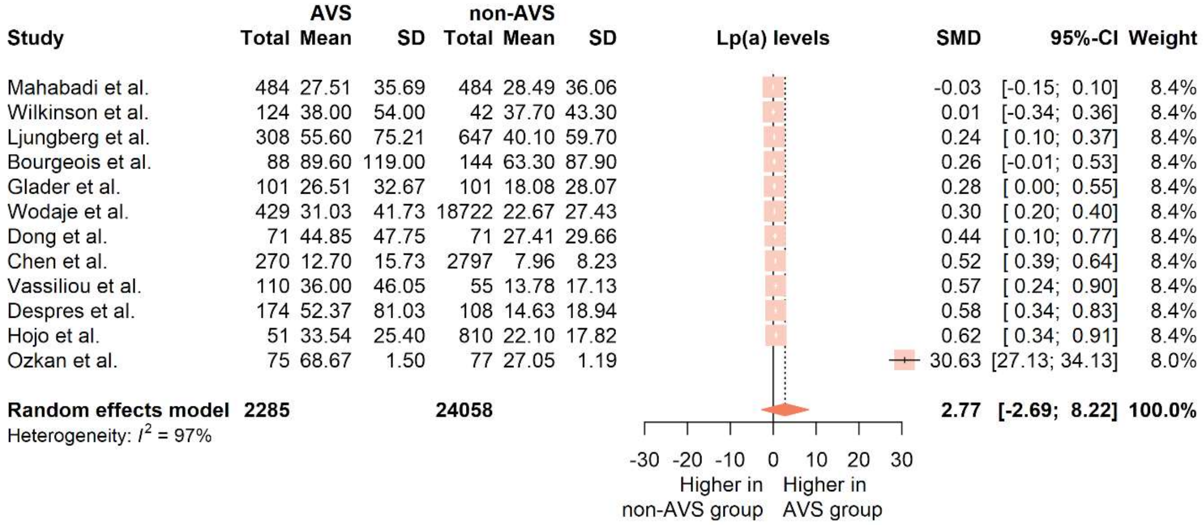

After excluding one outlying and influential study (Ozkan et al.[46]), the updated meta-analysis (11 studies[17,23–26,29,41,42,50,54,55]; n=26,191 individuals) showed significantly higher Lp(a) levels for the AVS group, with an SMD of 0.34 (95% CI: 0.19 – 0.48; p<0.001).

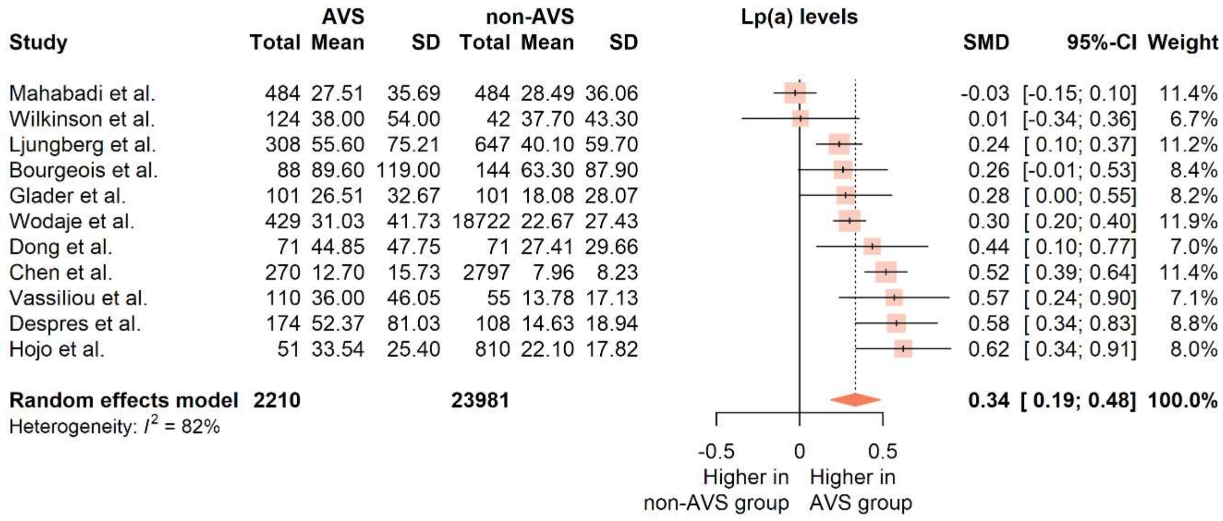

Egger's test did not point out significant asymmetry (p=0.49), indicating a low risk of publication bias. The following contour-enhanced funnel plot for the initial group of studies (outliers excluded) visualises potential asymmetry:

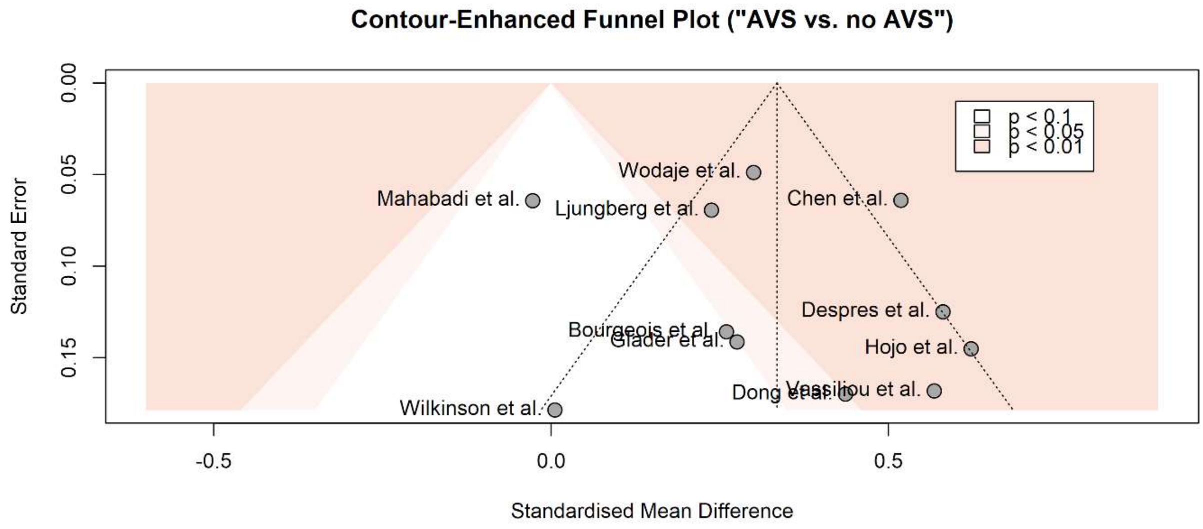

This result was also confirmed by the sensitivity analysis of studies reporting Lp(a) levels in nmol/L (5 studies; n=5,858 individuals).[17,24,25,41,55] AVS patients were found with significantly higher Lp(a) levels, by a mean difference of 22.63 nmol/L (95% CI: 9.98 – 35.27; p=0.008; I^2=53.2%), as compared with non-AVS ones.

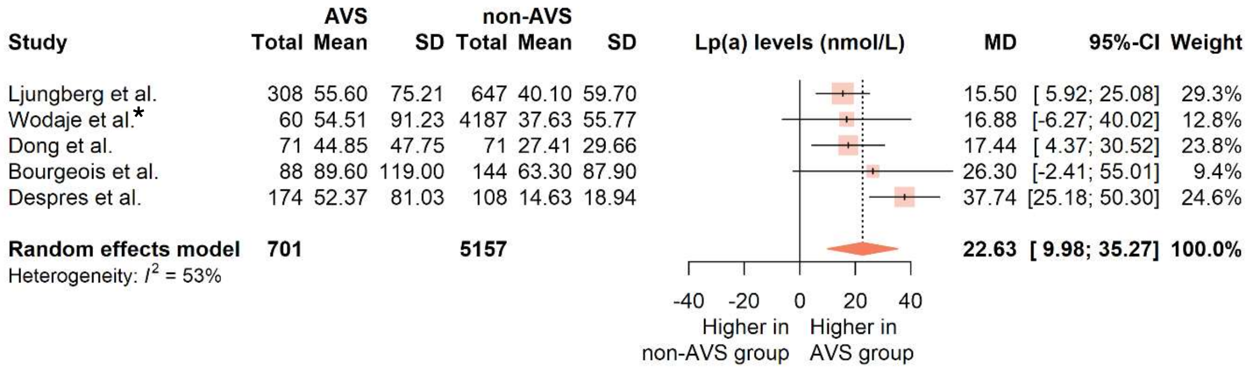

* Only the subcohort of indiduals with measurements in nmol/L (n=4,247) was leveraged from the study by Wodaje et al.[55]

Lp(a) difference and AVS severity

Three studies reported severity-wise data,[46,50,55] with one additional research team offering relevant information after contacting them.[41] A total of 898 patients were included (57.2% with severe AVS; $age_{weighted\text{-}mean}$: 63 years; $females_{weighted\text{-}mean}$: 47.8%). The meta-analysis showed no significant difference in Lp(a) levels between patients with mild or moderate AVS and those with severe (SMD 0.21; 95% CI: -0.12 to 0.54; p=0.130).

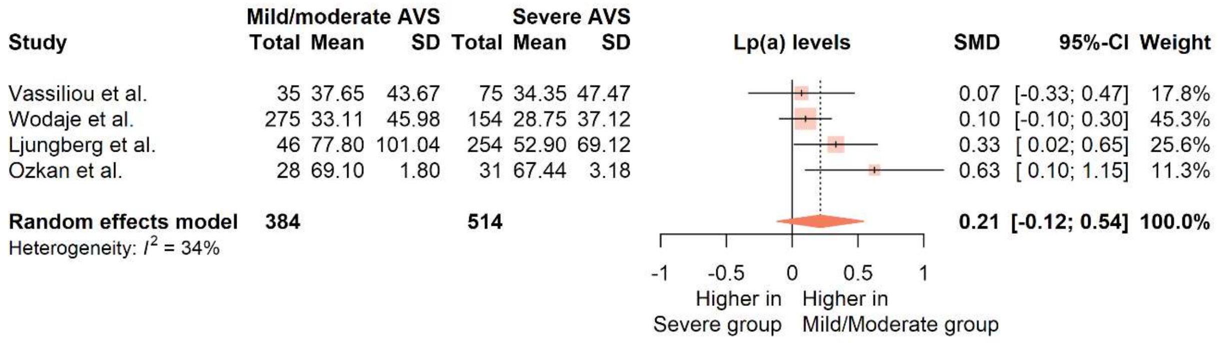

Similarly, the analysis of patients with mild AVS, against those with severe, yielded non-significant results (SMD 0.54; 95% CI: -0.24 to 1.31; I^2=75.8%).

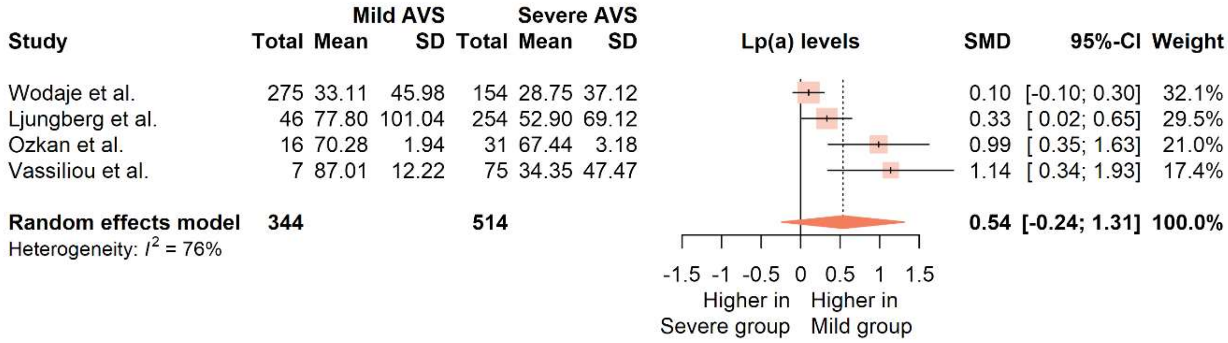

This result was retained after omitting the (identified as heavily influential) study by Wodaje et al.[55] (SMD 0.73; 95% CI: -0.38 to 1.84; p=0.105).

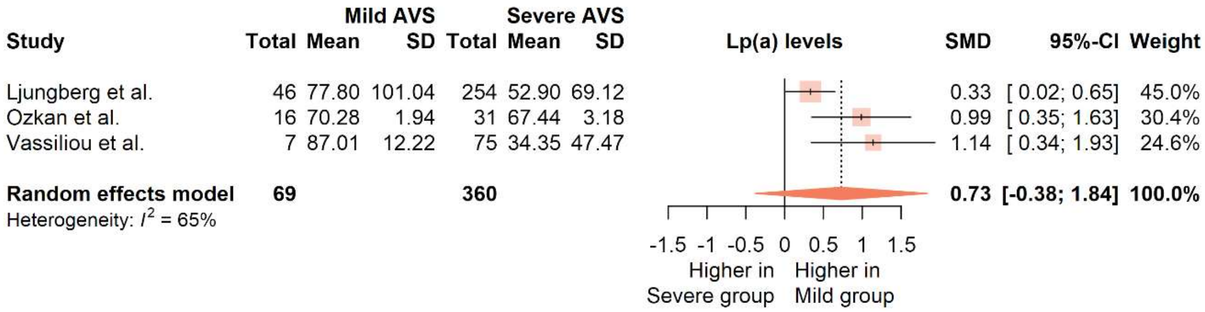

Meta-regression of age and sex effects on Lp(a) level difference

Meta-regressing 11 studies, after excluding Ozkan et al. as an outlier,[46] both age and sex were found significant predictors of the Lp(a) difference between AVS and non-AVS patients, explaining R^2: 72.14% of heterogeneity. Younger groups were found with a higher Lp(a) difference ($\beta_{mean-age}$: -0.02; 95% CI: -0.035 to -0.006; p=0.012), while studies with more female subjects presented with lower Lp(a) differences ($\beta_{female-\%}$: -0.017; 95% CI: -0.03 to -0.004; p=0.017).

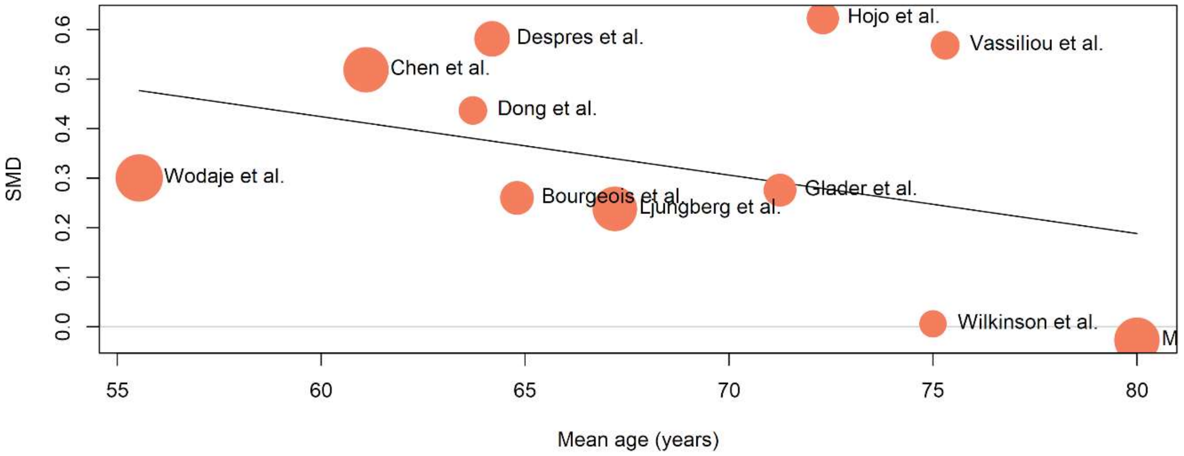

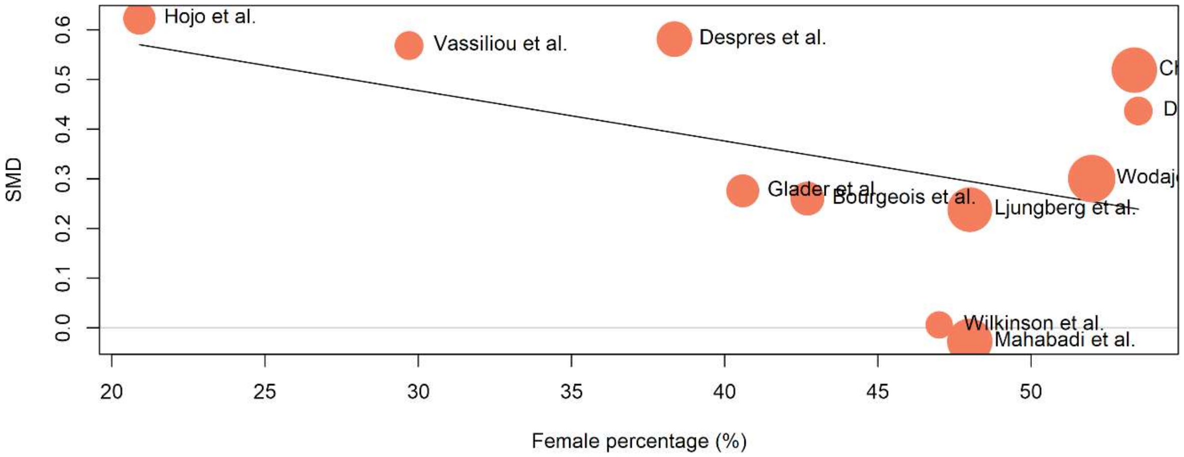

Annualised peak aortic velocity change difference between low- and high-Lp(a) individuals

A total of two studies were included (n=349 individuals; 32.7% with elevated Lp(a), defined as values above the top tertile; baseline age$_{\text{weighted-mean}}$: 62.6 years; females$_{\text{weighted-mean}}$: 36.9%).[20,57] Patients with elevated Lp(a) showed higher annualised peak aortic velocity change, than those with low Lp(a) levels, by a pooled MD of 0.09 m/s/year (95% CI: 0.09 - 0.09; p<0.001; I^2=0%).

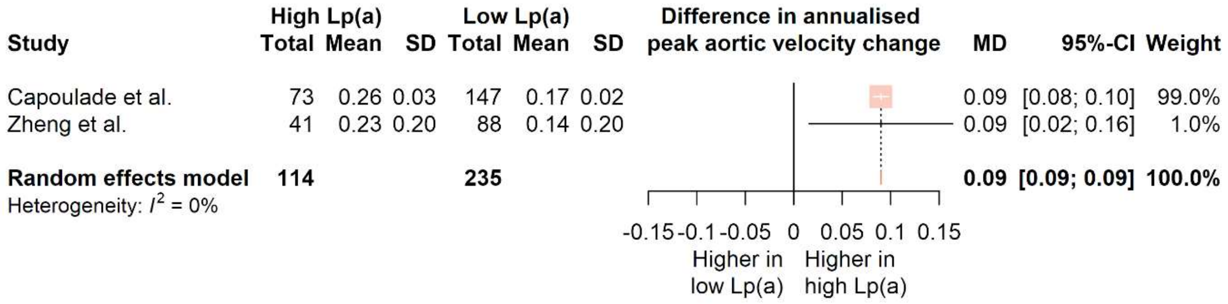

The following contour-enhanced funnel plot shows absence of asymmetry:

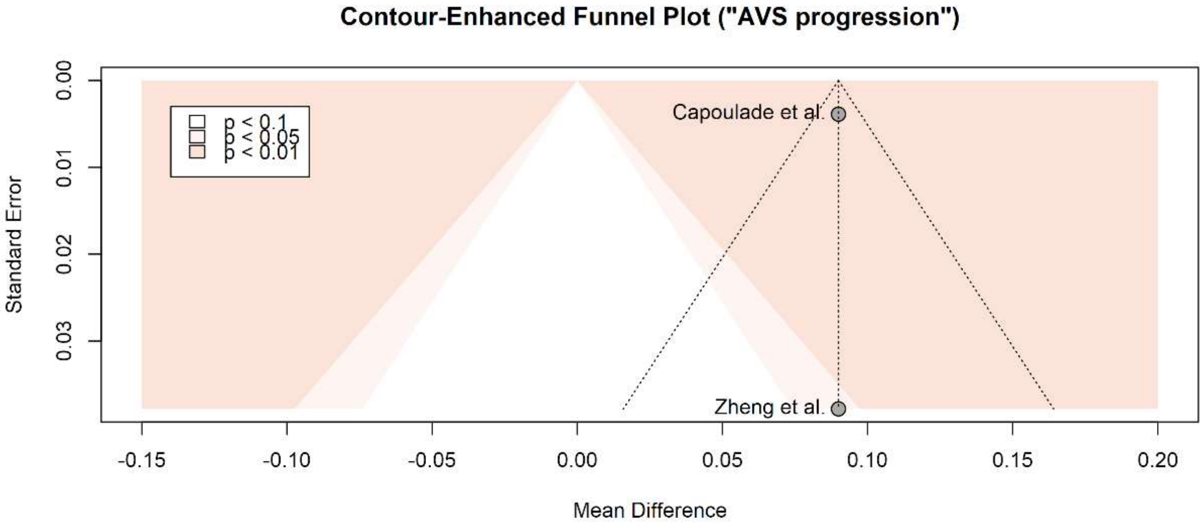

Risk of adverse outcomes between low- and high-Lp(a) individuals

A total of five studies were initially included (n=30,411 individuals; baseline age$_{weighted\text{-}mean}$: 57.9 years; females$_{weighted\text{-}mean}$: 53.4%),[20,40,55–57] showing an increased risk of adverse outcomes (death, aortic valve replacement, AVS-related hospitalisation) for high-Lp(a) individuals (HR: 1.39; 95% CI: 1.01 – 1.90; p=0.042; I^2=63%).

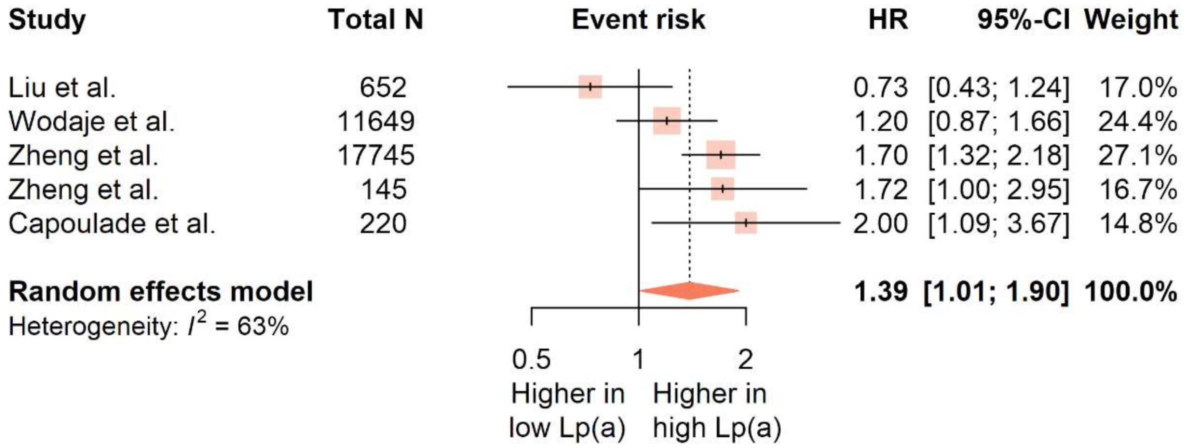

After excluding influential studies (Liu et al.[40]), the updated meta-analysis (four studies[20,55–57]; n=29,759 patients) yielded a similar result, with a pooled HR of 1.56 (95% CI: 1.11 – 2.18; p=0.025; I^2=20.6%).

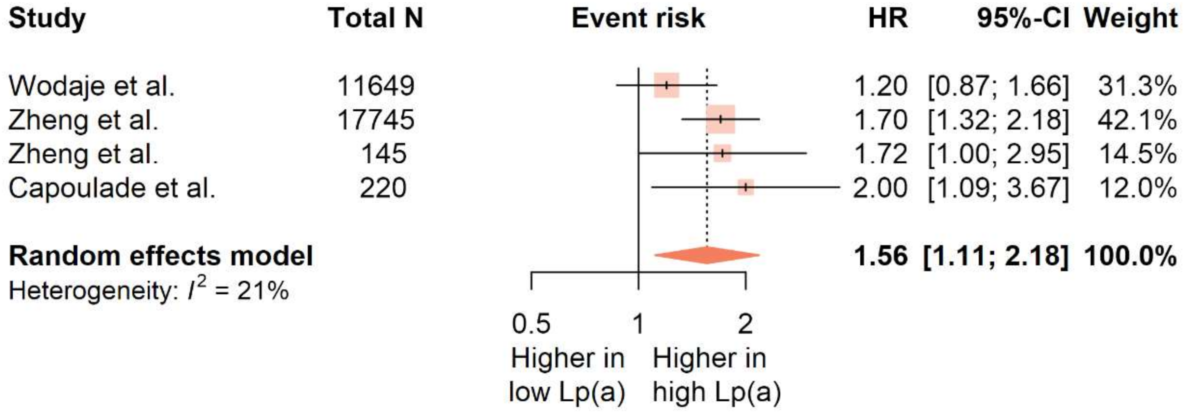

Egger's test did not point out significant asymmetry (p=0.7), indicating a low risk of publication bias. The following contour-enhanced funnel plot visualises potential asymmetry:

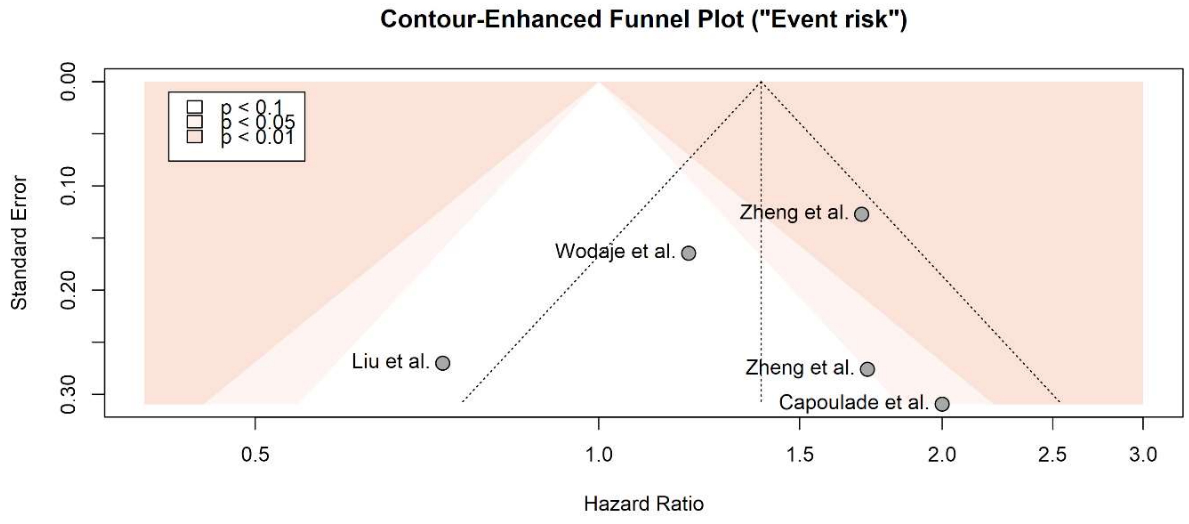

Meta-regression of age and sex effects on adverse event risk

Age and sex were not found to significantly affect the outcome (HR for serious adverse outcomes) between low- and high-Lp(a) individuals (p=0.84 and 0.86, respectively).

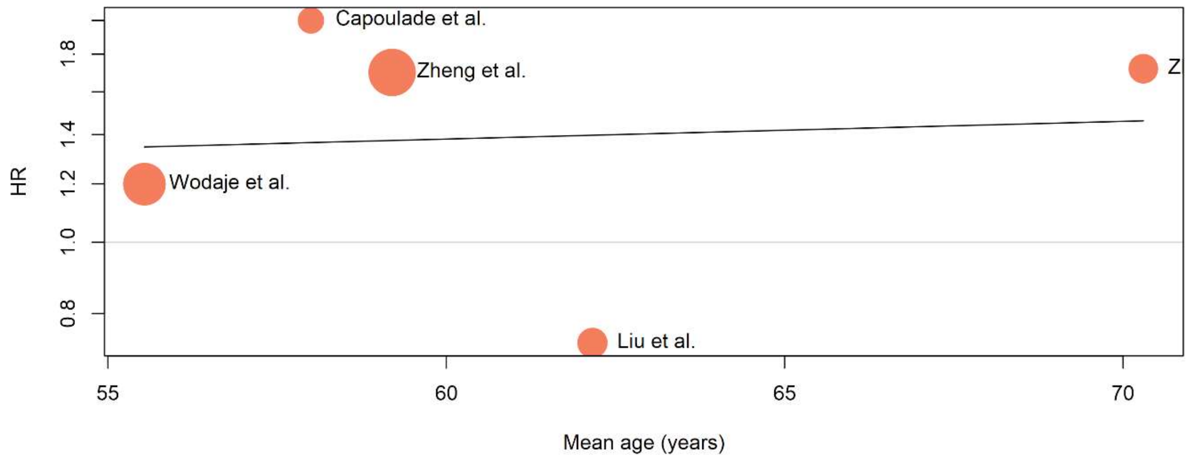

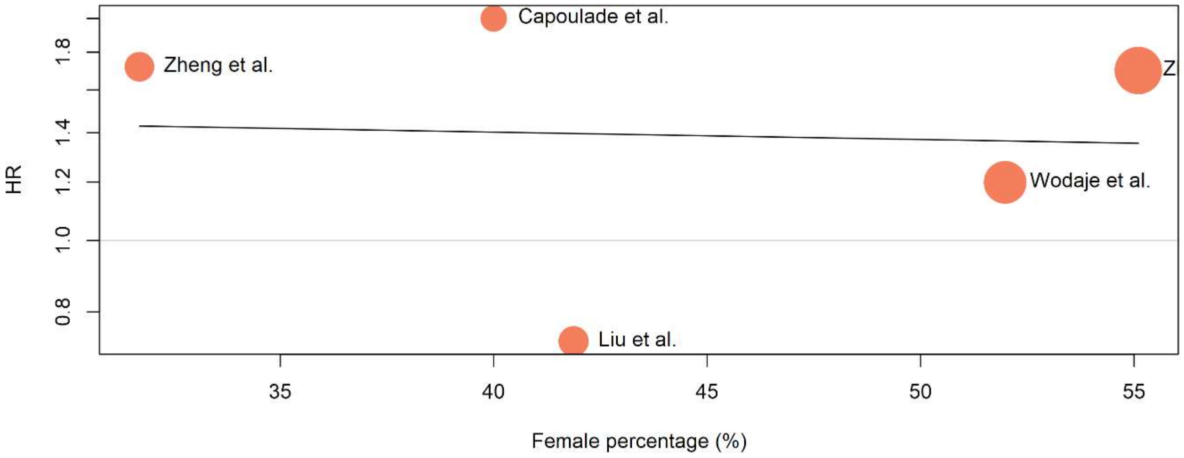

Risk of AVS and rs10455872 (effect allele G)

A total of 13 cohorts, included in eight studies,[14,46,58,60,62,63,65,67] were initially considered (n=967,175 individuals; baseline age$_{weighted\text{-}mean}$: 58.4 years; females$_{weighted\text{-}mean}$: 52.8%), with a non-significant pooled OR for rs10455872 effect (minor) G allele (OR: 1.07; 95% CI: 0.58 – 1.96; p=0.825; I^2=82.8%).

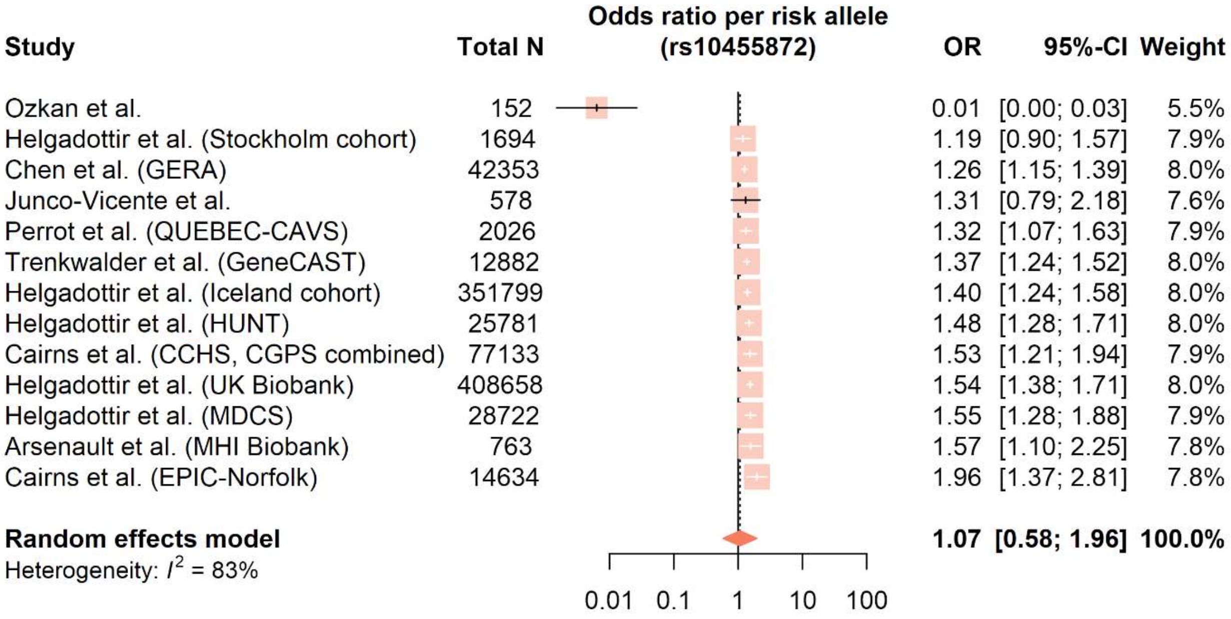

After excluding outliers / influential studies (Ozkan et al.[46]), the remaining seven studies with 12 cohorts; n=967,023 subjects) produced a significantly higher risk of AVS for patients with rs10455872 G allele (OR: 1.42; 95% CI: 1.34 - 1.50; p<0.001; I^2=28.8%).

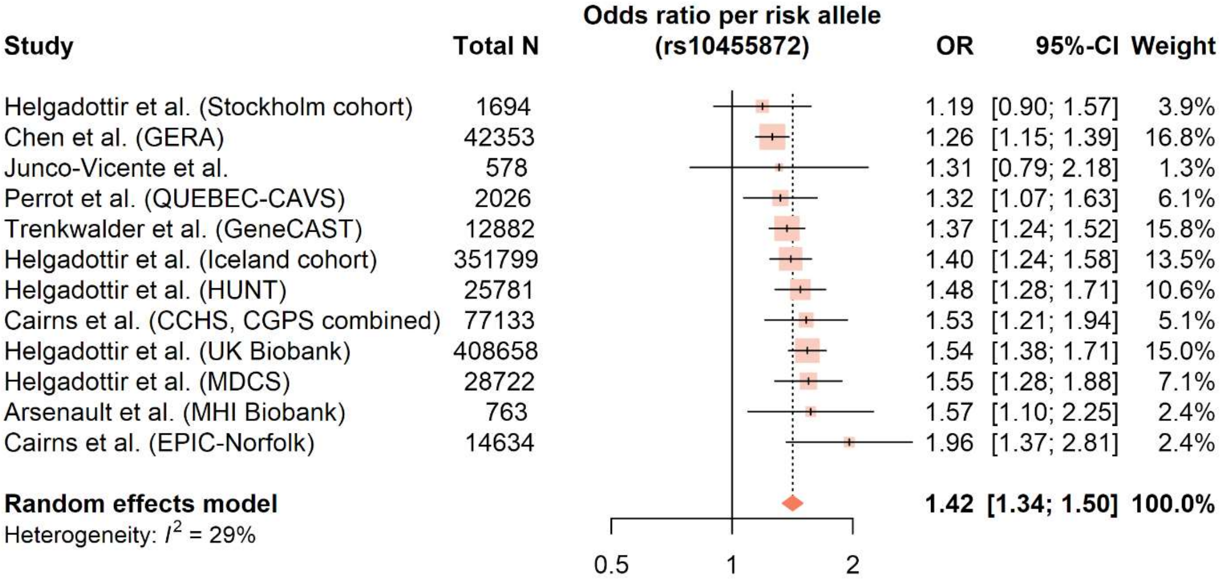

Egger's test did not show significant asymmetry (p=0.28), with the following contour-enhanced funnel plot:

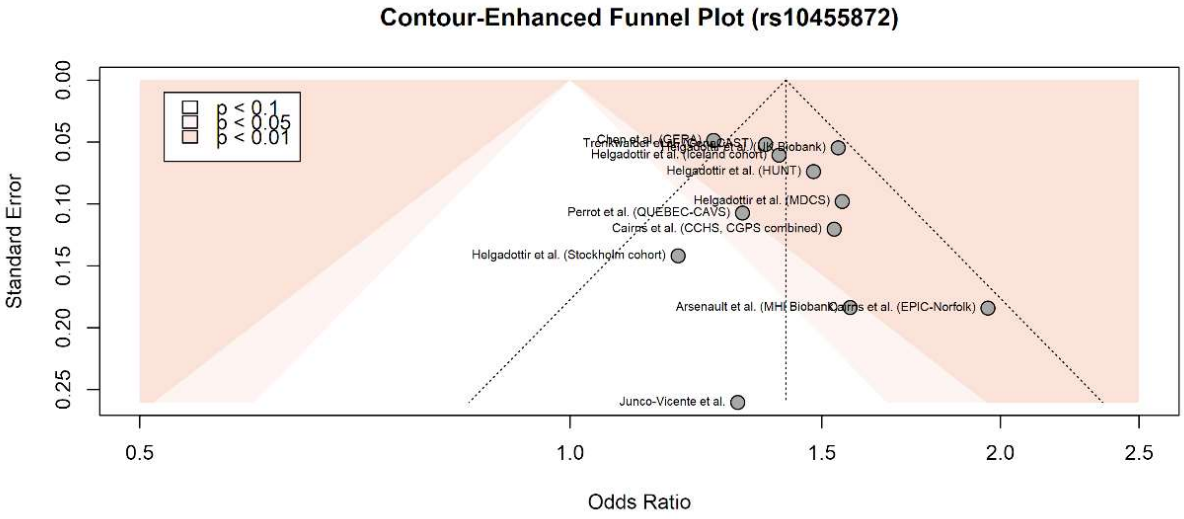

Meta-regression of age and sex impact on rs10455872 (allele G) effect size

Age was inversely correlated with the effect of rs10455872 minor G allele on AVS, with a smaller effect size in older ages ($\beta_{mean-age}$: -0.013; 95% CI: -0.021 to -0.005; p=0.008).

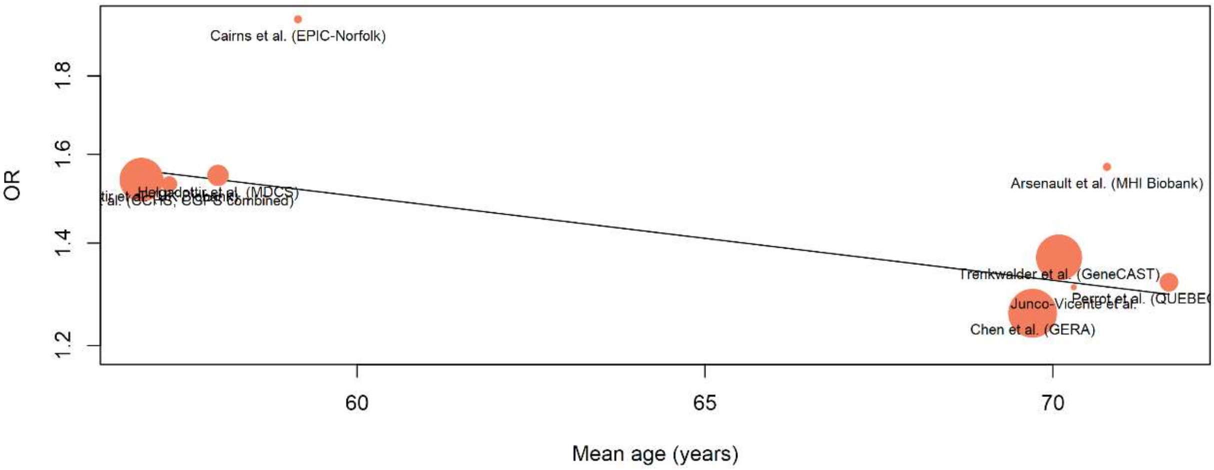

No similar association was found for sex (p=0.217).

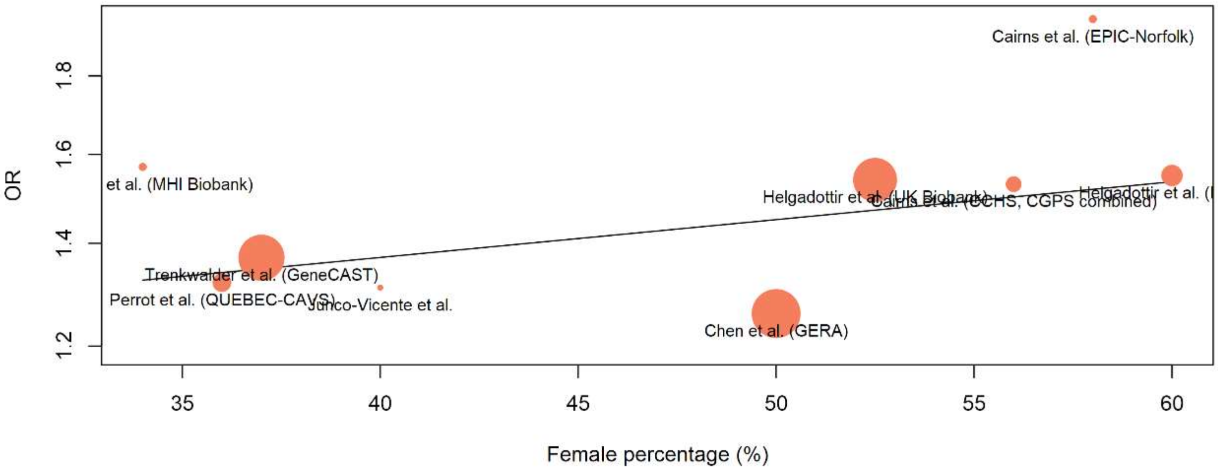

Risk of AVS and rs3798220 (effect allele C)

A total of four cohorts, from three studies,[58,60,65] were included (n=132,689 individuals; baseline $age_{weighted\text{-}mean}$: 61.4 years; $females_{weighted\text{-}mean}$: 53.5%), yielding a significant pooled OR for rs3798220 effect (minor) C allele (OR: 1.27; 95% CI: 1.09 - 1.48; p=0.002; I^2=0%).

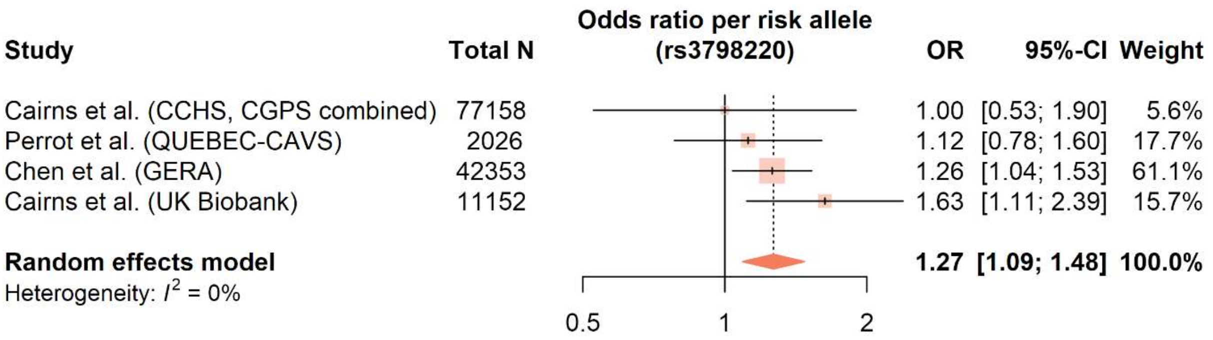

Egger's test did not reveal significant asymmetry (p=0.86), with the following contour-enhanced funnel plot:

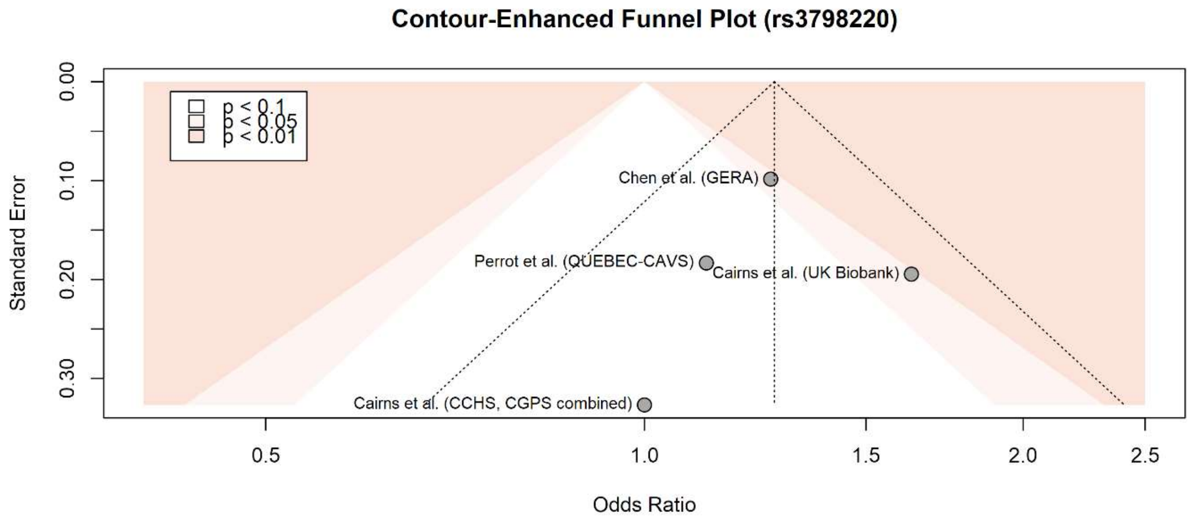

Meta-regression of age and sex impact on rs3798220 (allele C) effect size

No significant association was observed between age or sex and the effect size of rs3798220 C allele on AVS (p=0.438 for age and p=0.557 for sex).

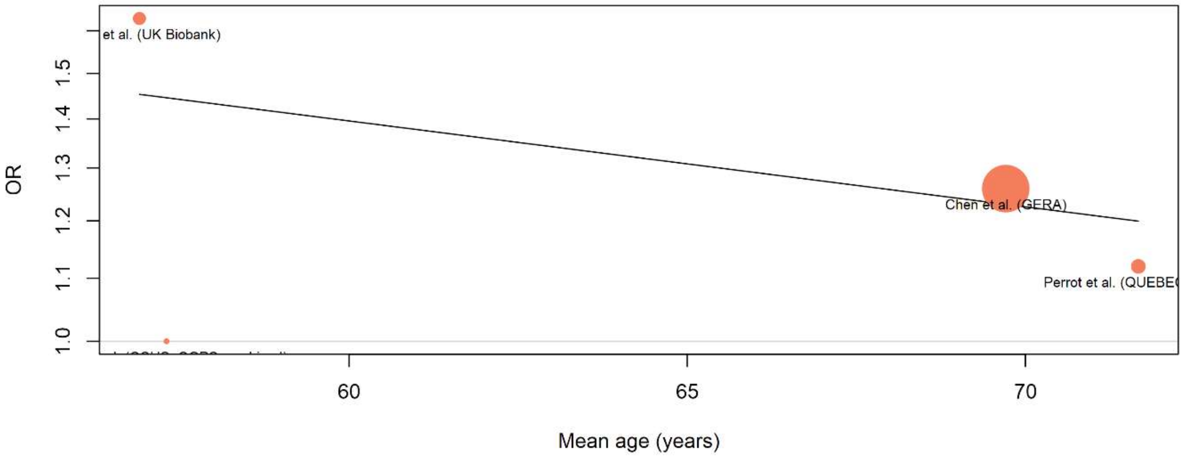

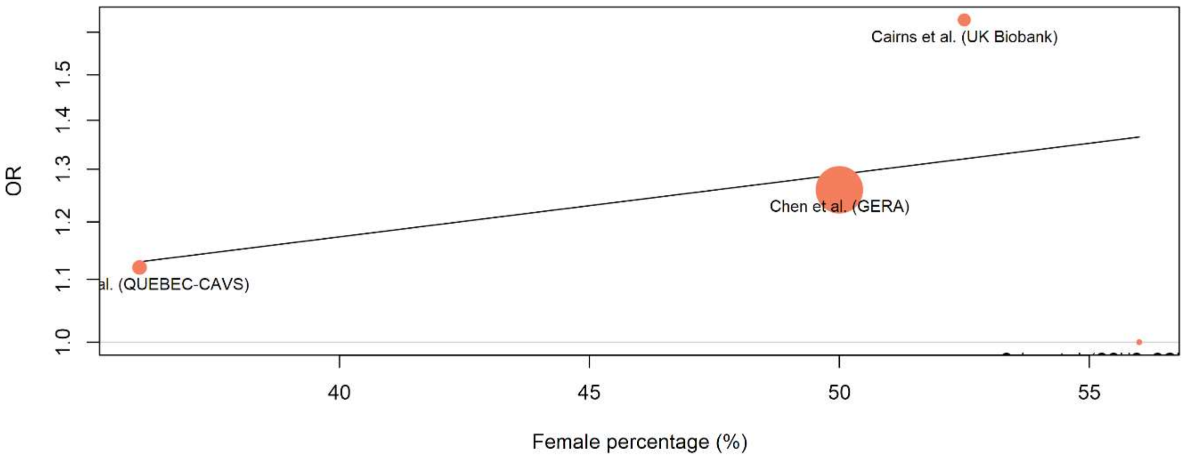

References

1. Wells GA, Shea B, O'Connell D al, Peterson J, Welch V, Losos M, Tugwell P. The Newcastle-Ottawa Scale (NOS) for Assessing the Quality of Nonrandomised Studies in Meta-Analyses

2. Herzog R, Álvarez-Pasquin MJ, Díaz C, Del Barrio JL, Estrada JM, Gil Á. Are healthcare workers' intentions to vaccinate related to their knowledge, beliefs and attitudes? a systematic review. *BMC Public Health* 2013;**13**:154.

3. Luo D, Wan X, Liu J, Tong T. Optimally estimating the sample mean from the sample size, median, mid-range, and/or mid-quartile range. *Stat Methods Med Res* 2018;**27**:1785–1805.

4. Wan X, Wang W, Liu J, Tong T. Estimating the sample mean and standard deviation from the sample size, median, range and/or interquartile range. *BMC Med Res Methodol* 2014;**14**:135.

5. Higgins J, Thomas J, Chandler J, Cumpston M, Li T, Page M, Welch V. *Cochrane Handbook for Systematic Reviews of Interventions*. 6th edition. Cochrane; 2022.

6. Knapp G, Hartung J. Improved tests for a random effects meta-regression with a single covariate. *Stat Med* 2003;**22**:2693–2710.

7. Higgins JPT, Thompson SG. Quantifying heterogeneity in a meta-analysis. *Stat Med* 2002;**21**:1539–1558.

8. Viechtbauer W, Cheung MW-L. Outlier and influence diagnostics for meta-analysis. *Res Synth Methods* 2010;**1**:112–125.

9. Egger M, Smith GD, Schneider M, Minder C. Bias in Meta-Analysis Detected by a Simple, Graphical Test. *BMJ* 1997;**315**:629–634.

10. R Core Team. *R: A Language and Environment for Statistical Computing*. Vienna, Austria: R Foundation for Statistical Computing; 2013.

11. Harrer M, Cuijpers P, Furukawa T, Ebert DD. *dmetar: Companion R Package For The Guide 'Doing Meta-Analysis in R'*. 2019.

12. Balduzzi S, Rücker G, Schwarzer G. How to perform a meta-analysis with R: a practical tutorial. *Evid Based Ment Health* 2019;**22**:153–160.

13. Viechtbauer W. Conducting Meta-Analyses in R with the metafor Package. *J Stat Softw* 2010;**36**.

14. Arsenault BJ, Boekholdt SM, Dubé M-P, Rhéaume E, Wareham NJ, Khaw K-T, Sandhu MS, Tardif J-C. Lipoprotein(a) levels, genotype, and incident aortic valve stenosis: a prospective Mendelian randomization study and replication in a case-control cohort. *Circ Cardiovasc Genet* 2014;**7**:304–310.

15. Boakye E, Dardari Z, Obisesan OH, Osei AD, Wang FM, Honda Y, Dzaye O, Osuji N, Carr JJ, Howard-Claudio CM, Wagenknecht L, Konety S, Coresh J, Matsushita K, Blaha MJ, Whelton SP. Sex-and race-specific burden of aortic valve calcification among older adults without overt coronary heart disease: The Atherosclerosis Risk in Communities Study. *Atherosclerosis* 2022;**355**:68–75.

16. Bortnick AE, Bartz TM, Ix JH, Chonchol M, Reiner A, Cushman M, Owens D, Barasch E, Siscovick DS, Gottdiener JS, Kizer JR. Association of inflammatory, lipid and mineral markers with cardiac calcification in older adults. *Heart Br Card Soc* 2016;**102**:1826–1834.

17. Bourgeois R, Devillers R, Perrot N, Després A-A, Boulanger M-C, Mitchell PL, Guertin J, Couture P, Boffa MB, Scipione CA, Pibarot P, Koschinsky ML, Mathieu P, Arsenault BJ. Interaction of Autotaxin With Lipoprotein(a) in Patients With Calcific Aortic Valve Stenosis. *JACC Basic Transl Sci* 2020;**5**:888–897.

18. Bozbas H, Yildirir A, Atar I, Pirat B, Eroglu S, Aydinalp A, Ozin B, Muderrisoglu H. Effects of serum levels of novel atherosclerotic risk factors on aortic valve calcification. *J Heart Valve Dis* 2007;**16**:387–393.

19. Cao J, Steffen BT, Guan W, Budoff M, Michos ED, Kizer JR, Post WS, Tsai MY. Evaluation of Lipoprotein(a) Electrophoretic and Immunoassay Methods in Discriminating Risk of Calcific Aortic Valve Disease and Incident Coronary Heart Disease: The Multi-Ethnic Study of Atherosclerosis. *Clin Chem* 2017;**63**:1705–1713.

20. Capoulade R, Chan KL, Yeang C, Mathieu P, Bossé Y, Dumesnil JG, Tam JW, Teo KK, Mahmut A, Yang X, Witztum JL, Arsenault BJ, Després J-P, Pibarot P, Tsimikas S. Oxidized Phospholipids, Lipoprotein(a), and Progression of Calcific Aortic Valve Stenosis. *J Am Coll Cardiol* 2015;**66**:1236–1246.

21. Capoulade R, Yeang C, Chan KL, Pibarot P, Tsimikas S. Association of Mild to Moderate Aortic Valve Stenosis Progression With Higher Lipoprotein(a) and Oxidized Phospholipid Levels: Secondary Analysis of a Randomized Clinical Trial. *JAMA Cardiol* 2018;**3**:1212.

22. Capoulade R, Torzewski M, Mayr M, Chan K-L, Mathieu P, Bossé Y, Dumesnil JG, Tam J, Teo KK, Burnap SA, Schmid J, Gobel N, Franke UFW, Sanchez A, Witztum JL, Yang X, Yeang C, Arsenault B, Després J-P, Pibarot P, Tsimikas S. ApoCIII-Lp(a) complexes in conjunction with Lp(a)-OxPL predict rapid progression of aortic stenosis. *Heart* 2020;**106**:738–745.

23. Chen J, Lyu L, Shen J, Pan Y, Jing J, Wang Y-J, Wei T. Epidemiological study of calcified aortic valve stenosis in a Chinese community population. *Postgrad Med J* 2022:postgradmedj-2022-141721.

24. Després A-A, Perrot N, Poulin A, Tastet L, Shen M, Chen HY, Bourgeois R, Trottier M, Tessier M, Guimond J, Nadeau M, Engert JC, Thériault S, Bossé Y, Witztum JL, Couture P, Mathieu P, Dweck MR, Tsimikas S, Thanassoulis G, Pibarot P, Clavel M-A, Arsenault BJ. Lipoprotein(a), Oxidized Phospholipids, and Aortic Valve Microcalcification Assessed by 18F-Sodium Fluoride Positron Emission Tomography and Computed Tomography. *CJC Open* 2019;**1**:131–140.

25. Dong H, Cong H, Wang J, Jiang Y, Liu C, Zhang Y, Zhu Y, Wang Q. Correlations between lipoprotein(a) gene polymorphisms and calcific aortic valve disease and coronary heart disease in Han Chinese. *J Int Med Res* 2020;**48**:300060520965353.

26. Glader CA, Birgander LS, Söderberg S, Ildgruben HP, Saikku P, Waldenström A, Dahlén GH. Lipoprotein(a), Chlamydia pneumoniae, leptin and tissue plasminogen activator as risk markers for valvular aortic stenosis. *Eur Heart J* 2003;**24**:198–208.

27. Gotoh T, Kuroda T, Yamasawa M, Nishinaga M, Mitsuhashi T, Seino Y, Nagoh N, Kayaba K, Yamada S, Matsuo H. Correlation between lipoprotein(a) and aortic valve sclerosis assessed by echocardiography (the JMS Cardiac Echo and Cohort Study). *Am J Cardiol* 1995;**76**:928–932.

28. Gudbjartsson DF, Thorgeirsson G, Sulem P, Helgadottir A, Gylfason A, Saemundsdottir J, Bjornsson E, Norddahl GL, Jonasdottir A, Jonasdottir A, Eggertsson HP, Gretarsdottir S, Thorleifsson G, Indridason OS, Palsson R, Jonasson F, Jonsdottir I, Eyjolfsson GI, Sigurdardottir O, Olafsson I, Danielsen R, Matthiasson SE, Kristmundsdottir S, Halldorsson BV, Hreidarsson AB, Valdimarsson EM, Gudnason T, Benediktsson R, Steinthorsdottir V, Thorsteinsdottir U, Holm H, Stefansson K. Lipoprotein(a) Concentration and Risks of Cardiovascular Disease and Diabetes. *J Am Coll Cardiol* 2019;**74**:2982–2994.

29. Hojo Y, Kumakura H, Kanai H, Iwasaki T, Ichikawa S, Kurabayashi M. Lipoprotein(a) is a risk factor for aortic and mitral valvular stenosis in peripheral arterial disease. *Eur Heart J Cardiovasc Imaging* 2016;**17**:492–497.

30. Hovland A, Narverud I, Lie Øyri LK, Bogsrud MP, Aagnes I, Ueland T, Mulder M, Leijten F, Langslet G, Wium C, Svilaas A, Arnesen KE, Roeters van Lennep J, Aukrust P, Halvorsen B, Retterstøl K, Holven KB. Subjects with familial hypercholesterolemia have lower aortic valve area and higher levels of inflammatory biomarkers. *J Clin Lipidol* 2021;**15**:134–141.

31. Kaiser Y, Nurmohamed NS, Kroon J, Verberne HJ, Tzolos E, Dweck MR, Somsen AG, Arsenault BJ, Stroes ESG, Zheng KH, Boekholdt SM. Lipoprotein(a) has no major impact on calcification activity in patients with mild to moderate aortic valve stenosis. *Heart Br Card Soc* 2022;**108**:61–66.

32. Kaiser Y, Singh SS, Zheng KH, Verbeek R, Kavousi M, Pinto S-J, Vernooij MW, Sijbrands EJG, Boekholdt SM, Rijke YB de, Stroes ESG, Bos D. Lipoprotein(a) is robustly associated with aortic valve calcium. *Heart Br Card Soc* 2021;**107**:1422–1428.

33. Kaiser Y, Toorn JE van der, Singh SS, Zheng KH, Kavousi M, Sijbrands EJG, Stroes ESG, Vernooij MW, Rijke YB de, Boekholdt SM, Bos D. Lipoprotein(a) is associated with the onset but not the progression of aortic valve calcification. *Eur Heart J* 2022;**43**:3960–3967.

34. Kaltoft M, Sigvardsen PE, Afzal S, Langsted A, Fuchs A, Kühl JT, Køber L, Kamstrup PR, Kofoed KF, Nordestgaard BG. Elevated lipoprotein(a) in mitral and aortic valve calcification and disease: The Copenhagen General Population Study. *Atherosclerosis* 2022;**349**:166–174.

35. Kaltoft M, Langsted A, Afzal S, Kamstrup PR, Nordestgaard BG. Lipoprotein(a) and Body Mass Compound the Risk of Calcific Aortic Valve Disease. *J Am Coll Cardiol* 2022;**79**:545–558.

36. Kamstrup PR, Tybjærg-Hansen A, Nordestgaard BG. Elevated lipoprotein(a) and risk of aortic valve stenosis in the general population. *J Am Coll Cardiol* 2014;**63**:470–477.

37. Kamstrup PR, Hung M-Y, Witztum JL, Tsimikas S, Nordestgaard BG. Oxidized Phospholipids and Risk of Calcific Aortic Valve Disease: The Copenhagen General Population Study. *Arterioscler Thromb Vasc Biol* 2017;**37**:1570–1578.

38. Langsted A, Varbo A, Kamstrup PR, Nordestgaard BG. Elevated Lipoprotein(a) Does Not Cause Low-Grade Inflammation Despite Causal Association With Aortic Valve Stenosis and Myocardial Infarction: A Study of 100,578 Individuals from the General Population. *J Clin Endocrinol Metab* 2015;**100**:2690–2699.

39. Littmann K, Wodaje T, Alvarsson M, Bottai M, Eriksson M, Parini P, Brinck J. The Association of Lipoprotein(a) Plasma Levels With Prevalence of Cardiovascular Disease and Metabolic Control Status in Patients With Type 1 Diabetes. *Diabetes Care* 2020;**43**:1851–1858.

40. Liu S-L, Rozi R, Shi H-W, Gao Y, Guo Y-L, Tang Y-D, Li J-J, Wu N-Q. Association of serum lipoprotein(a) level with the severity and prognosis of calcific aortic valve stenosis: a Chinese cohort study. *J Geriatr Cardiol JGC* 2020;**17**:133–140.

41. Ljungberg J, Holmgren A, Bergdahl IA, Hultdin J, Norberg M, Näslund U, Johansson B, Söderberg S. Lipoprotein(a) and the Apolipoprotein B/A1 Ratio Independently Associate With Surgery for Aortic Stenosis Only in Patients With Concomitant Coronary Artery Disease. *J Am Heart Assoc* 2017;**6**.

42. Mahabadi AA, Kahlert P, Kahlert HA, Dykun I, Balcer B, Forsting M, Heusch G, Rassaf T. Comparison of Lipoprotein(a)-Levels in Patients ≥70 Years of Age With Versus Without Aortic Valve Stenosis. *Am J Cardiol* 2018;**122**:645–649.

43. Makshood M, Joshi PH, Kanaya AM, Ayers C, Budoff M, Tsai MY, Blaha M, Michos ED, Post WS. Lipoprotein (a) and aortic valve calcium in South Asians compared to other race/ethnic groups. *Atherosclerosis* 2020;**313**:14–19.

44. Nsaibia MJ, Mahmut A, Boulanger M-C, Arsenault BJ, Bouchareb R, Simard S, Witztum JL, Clavel M-A, Pibarot P, Bossé Y, Tsimikas S, Mathieu P. Autotaxin interacts with lipoprotein(a) and oxidized phospholipids in predicting the risk of calcific aortic valve stenosis in patients with coronary artery disease. *J Intern Med* 2016;**280**:509–517.

45. Obisesan OH, Kou M, Wang FM, Boakye E, Honda Y, Uddin SMI, Dzaye O, Osei AD, Orimoloye OA, Howard-Claudio CM, Coresh J, Blumenthal RS, Hoogeveen RC, Budoff MJ, Matsushita K, Ballantyne CM, Blaha MJ. Lipoprotein(a) and Subclinical Vascular and Valvular Calcification on Cardiac Computed Tomography: The Atherosclerosis Risk in Communities Study. *J Am Heart Assoc* 2022;**11**:e024870.

46. Ozkan, Ozcelik, Yildiz, Budak. Lipoprotein(a) Gene Polymorphism Increases a Risk Factor for Aortic Valve Calcification. *J Cardiovasc Dev Dis* 2019;**6**:31.

47. Simony SB, Mortensen MB, Langsted A, Afzal S, Kamstrup PR, Nordestgaard BG. Sex differences of lipoprotein(a) levels and associated risk of morbidity and mortality by age: The Copenhagen General Population Study. *Atherosclerosis* 2022;**355**:76–82.

48. Stewart BF, Siscovick D, Lind BK, Gardin JM, Gottdiener JS, Smith VE, Kitzman DW, Otto CM. Clinical factors associated with calcific aortic valve disease. Cardiovascular Health Study. *J Am Coll Cardiol* 1997;**29**:630–634.

49. Sticchi E, Giusti B, Cordisco A, Gori AM, Sereni A, Sofi F, Mori F, Colonna S, Fugazzaro MP, Pepe G, Nistri S, Marcucci R. Role of lipoprotein (a) and LPA KIV2 repeat polymorphism in bicuspid aortic valve stenosis and calcification: a proof of concept study. *Intern Emerg Med* 2019;**14**:45–50.

50. Vassiliou VS, Flynn PD, Raphael CE, Newsome S, Khan T, Ali A, Halliday B, Studer Bruengger A, Malley T, Sharma P, Selvendran S, Aggarwal N, Sri A, Berry H, Donovan J, Lam W, Auger D, Cook SA, Pennell DJ, Prasad SK. Lipoprotein(a) in patients with aortic stenosis: Insights from cardiovascular magnetic resonance. *PloS One* 2017;**12**:e0181077.

51. Vongpromek R, Bos S, Ten Kate G-JR, Yahya R, Verhoeven AJM, Feyter PJ de, Kronenberg F, Roeters van Lennep JE, Sijbrands EJG, Mulder MT. Lipoprotein(a) levels are associated with aortic valve calcification in asymptomatic patients with familial hypercholesterolaemia. *J Intern Med* 2015;**278**:166–173.

52. Wang W-G, He Y-F, Chen Y-L, Zhao F-M, Song Y-Q, Zhang H, Ma Y-H, Guan X, Zhang W-Y, Chen X-L, Liu C, Cong H-L. Proprotein convertase subtilisin/kexin type 9 levels and aortic valve calcification: A prospective, cross sectional study. *J Int Med Res* 2016;**44**:865–874.

53. Wang Z, Li M, Liu N. The nonlinear correlation between lipoprotein (a) and the prevalence of aortic valve calcification in patients with new-onset acute myocardial infarction. *Acta Cardiol* 2022;**77**:950–959.

54. Wilkinson MJ, Ma GS, Yeang C, Ang L, Strachan M, DeMaria AN, Tsimikas S, Cotter B. The Prevalence of Lipoprotein(a) Measurement and Degree of Elevation Among 2710 Patients With Calcific Aortic Valve Stenosis in an Academic Echocardiography Laboratory Setting. *Angiology* 2017;**68**:795–798.

55. Wodaje T, Littmann K, Häbel H, Bottai M, Bäck M, Parini P, Brinck J. Plasma Lipoprotein(a) measured in routine clinical care and the association with incident calcified aortic valve stenosis during a 14-year observational period. *Atherosclerosis* 2022;**349**:175–182.

56. Zheng KH, Arsenault BJ, Kaiser Y, Khaw K-T, Wareham NJ, Stroes ESG, Boekholdt SM. apoB/apoA-I Ratio and Lp(a) Associations With Aortic Valve Stenosis Incidence: Insights From the EPIC-Norfolk Prospective Population Study. *J Am Heart Assoc* 2019;**8**:e013020.

57. Zheng KH, Tsimikas S, Pawade T, Kroon J, Jenkins WSA, Doris MK, White AC, Timmers NKLM, Hjortnaes J, Rogers MA, Aikawa E, Arsenault BJ, Witztum JL, Newby DE, Koschinsky ML, Fayad ZA, Stroes ESG, Boekholdt SM, Dweck MR. Lipoprotein(a) and Oxidized Phospholipids Promote Valve Calcification in Patients With Aortic Stenosis. *J Am Coll Cardiol* 2019;**73**:2150–2162.

58. Cairns BJ, Coffey S, Travis RC, Prendergast B, Green J, Engert JC, Lathrop M, Thanassoulis G, Clarke R. A Replicated, Genome-Wide Significant Association of Aortic Stenosis With a Genetic Variant for Lipoprotein(a): Meta-Analysis of Published and Novel Data. *Circulation* 2017;**135**:1181–1183.

59. Cardoso-Saldaña G, Fragoso JM, Lale-Farjat S, Torres-Tamayo M, Posadas-Romero C, Vargas-Alarcón G, Posadas-Sánchez R. The rs10455872-G allele of the LPA gene is associated with high lipoprotein(a) levels and increased aortic valve calcium in a Mexican adult population. *Genet Mol Biol* 2019;**42**:519–525.

60. Chen HY, Dufresne L, Burr H, Ambikkumar A, Yasui N, Luk K, Ranatunga DK, Whitmer RA, Lathrop M, Engert JC, Thanassoulis G. Association of LPA Variants With Aortic Stenosis: A Large-Scale Study Using Diagnostic and Procedural Codes From Electronic Health Records. *JAMA Cardiol* 2018;**3**:18–23.

61. Emdin CA, Khera AV, Natarajan P, Klarin D, Won H-H, Peloso GM, Stitziel NO, Nomura A, Zekavat SM, Bick AG, Gupta N, Asselta R, Duga S, Merlini PA, Correa A, Kessler T, Wilson JG, Bown MJ, Hall AS, Braund PS, Samani NJ, Schunkert H, Marrugat J, Elosua R, McPherson R, Farrall M, Watkins H, Willer C, Abecasis GR, Felix JF, Vasan RS, Lander E, Rader DJ, Danesh J, Ardissino D, Gabriel S, Saleheen D, Kathiresan S. Phenotypic Characterization of Genetically Lowered Human Lipoprotein(a) Levels. *J Am Coll Cardiol* 2016;**68**:2761–2772.

62. Helgadottir A, Thorleifsson G, Gretarsdottir S, Stefansson OA, Tragante V, Thorolfsdottir RB, Jonsdottir I, Bjornsson T, Steinthorsdottir V, Verweij N, Nielsen JB, Zhou W, Folkersen L, Martinsson A, Heydarpour M, Prakash S, Oskarsson G, Gudbjartsson T, Geirsson A, Olafsson I, Sigurdsson EL, Almgren P, Melander O, Franco-Cereceda A, Hamsten A, Fritsche L, Lin M, Yang B, Hornsby W, Guo D, Brummett CM, Abecasis G, Mathis M, Milewicz D, Body SC, Eriksson P, Willer CJ, Hveem K, Newton-Cheh C, Smith JG, Danielsen R, Thorgeirsson G, Thorsteinsdottir U, Gudbjartsson DF, Holm H, Stefansson K. Genome-wide analysis yields new loci associating with aortic valve stenosis. *Nat Commun* 2018;**9**:987.

63. Junco-Vicente A, Solache-Berrocal G, Del Río-García Á, Rolle-Sóñora V, Areces S, Morís C, Martín M, Rodríguez I. IL6 gene polymorphism association with calcific aortic valve stenosis and influence on serum levels of interleukin-6. *Front Cardiovasc Med* 2022;**9**:989539.

64. Ozkan U, Ozcelik F, Yildiz M, Budak M. Lipoprotein(a) Gene Polymorphism Increases a Risk Factor for Aortic Valve Calcification. *J Cardiovasc Dev Dis* 2019;**6**.

65. Perrot N, Thériault S, Dina C, Chen HY, Boekholdt SM, Rigade S, Després A-A, Poulin A, Capoulade R, Le Tourneau T, Messika-Zeitoun D, Trottier M, Tessier M, Guimond J, Nadeau M, Engert JC, Khaw K-T, Wareham NJ, Dweck MR, Mathieu P, Pibarot P, Schott J-J, Thanassoulis G, Clavel M-A, Bossé Y, Arsenault BJ. Genetic Variation in LPA, Calcific Aortic Valve Stenosis in Patients Undergoing Cardiac Surgery, and Familial Risk of Aortic Valve Microcalcification. *JAMA Cardiol* 2019;**4**:620–627.

66. Thanassoulis G, Campbell CY, Owens DS, Smith JG, Smith AV, Peloso GM, Kerr KF, Pechlivanis S, Budoff MJ, Harris TB, Malhotra R, O'Brien KD, Kamstrup PR, Nordestgaard BG, Tybjaerg-Hansen A, Allison MA, Aspelund T, Criqui MH, Heckbert SR, Hwang S-J, Liu Y, Sjogren M, Pals J van der, Kälsch H, Mühleisen TW, Nöthen MM, Cupples LA, Caslake M, Di Angelantonio E, Danesh J, Rotter

JI, Sigurdsson S, Wong Q, Erbel R, Kathiresan S, Melander O, Gudnason V, O'Donnell CJ, Post WS, CHARGE Extracoronary Calcium Working Group. Genetic associations with valvular calcification and aortic stenosis. *N Engl J Med* 2013;**368**:503–512.

67. Trenkwalder T, Nelson CP, Musameh MD, Mordi IR, Kessler T, Pellegrini C, Debiec R, Rheude T, Lazovic V, Zeng L, Martinsson A, Gustav Smith J, Gådin JR, Franco-Cereceda A, Eriksson P, Nielsen JB, Graham SE, Willer CJ, Hveem K, Kastrati A, Braund PS, Palmer CNA, Aracil A, Husser O, Koenig W, Schunkert H, Lang CC, Hengstenberg C, Samani NJ. Effects of the coronary artery disease associated LPA and 9p21 loci on risk of aortic valve stenosis. *Int J Cardiol* 2019;**276**:212–217.
